# Comprehensive evaluation of smoking exposures and their interactions on DNA methylation

**DOI:** 10.1016/j.ebiom.2023.104956

**Published:** 2024-01-09

**Authors:** Thanh T. Hoang, Yunsung Lee, Daniel L. McCartney, Elin T.G. Kersten, Christian M. Page, Paige M. Hulls, Mikyeong Lee, Rosie M. Walker, Charles E. Breeze, Brian D. Bennett, Adam B. Burkholder, James Ward, Anne Lise Brantsæter, Ida H. Caspersen, Alison A. Motsinger-Reif, Marie Richards, Julie D. White, Shanshan Zhao, Rebecca C. Richmond, Maria C. Magnus, BIOS Consortium, Bastiaan Heijmans, Peter ’t Hoen, Joyce van Meurs, Rick Jansen, Lude Franke, Dorret Boomsma, René Pool, Jenny van Dongen, Jouke Hottenga, Marleen van Greevenbroek, Coen Stehouwer, Carla van der Kallen, Casper Schalkwijk, Cisca Wijmenga, Sasha Zhernakova, Ettje Tigchelaar, P. Eline Slagboom, Marian Beekman, Joris Deelen, Diana Van Heemst, Jan Veldink, Leonard van den Berg, Cornelia van Duijn, Bert Hofman, Aaron Isaacs, André Uitterlinden, P. Mila Jhamai, Michael Verbiest, H. Eka Suchiman, Marijn Verkerk, Ruud van der Breggen, Jeroen van Rooij, Nico Lakenberg, Hailiang Mei, Maarten van Iterson, Michiel van Galen, Jan Bot, Dasha Zhernakova, Peter van ‘t Hof, Patrick Deelen, Irene Nooren, Matthijs Moed, Martijn Vermaat, René Luijk, Marc Bonder, Freerk van Dijk, Wibowo Arindrarto, Szymon Kielbasa, Morris Swertz, Erik van Zwet, Gerard H. Koppelman, Kathryn L. Evans, Riccardo E. Marioni, Siri E. Håberg, Stephanie J. London

**Affiliations:** a Epidemiology Branch, National Institute of Environmental Health Sciences, National Institutes of Health, Research Triangle Park, NC, USA; b Department of Pediatrics, Division of Hematology-Oncology, Department of Pediatrics, Baylor College of Medicine, Houston, TX, USA; c Dan L. Duncan Comprehensive Cancer Center, Baylor College of Medicine, Houston, TX, USA; d Cancer and Hematology Center, Texas Children’s Hospital, Houston, TX, USA; e Centre for Fertility and Health, Norwegian Institute of Public Health, Oslo, Norway; f Centre for Genomic and Experimental Medicine, Institute of Genetics and Cancer, University of Edinburgh, Crewe Road South, Edinburgh EH4 2XU, UK; g University of Groningen, University Medical Center Groningen, Beatrix Children’s Hospital, Dept. of Pediatric Pulmonology and Pediatric Allergy, Groningen, the Netherlands; h University of Groningen, University Medical Center Groningen, GRIAC Research Institute, Groningen, the Netherlands; i Department of Physical Health and Ageing, Division for Physical and Mental Health, Norwegian Institute of Public Health, Oslo, Norway; j Population Health Sciences, Bristol Medical School, University of Bristol, BS8 2BN, UK; k MRC Integrative Epidemiology Unit at University of Bristol, BS8 2BN, UK; l Centre for Clinical Brain Sciences, University of Edinburgh, Chancellor’s Building, 49 Little France Crescent, Edinburgh EH16 4SB, UK; m School of Psychology, University of Exeter, Perry Road, Exeter, UK; n UCL Cancer Institute, University College London, Paul O’Gorman Building, London, UK; o Altius Institute for Biomedical Sciences, Seattle, WA, USA; p Department of Health and Human Services, Integrative Bioinformatics Support Group, National Institutes of Health, Research Triangle Park, NC, USA; q Department of Health and Human Services, Office of Environmental Science Cyberinfrastructure, National Institute of Environmental Health Sciences, National Institutes of Health, Research Triangle Park, NC, USA; r Department of Food Safety, Division of Climate and Environmental Health, Norwegian Institute of Public Health, Oslo, Norway; s Department of Health and Human Services, Biostatistics and Computational Biology Branch, National Institute of Environmental Health Sciences, National Institutes of Health, Research Triangle Park, NC, USA; t Westat, Durham, NC, USA; u GenOmics and Translational Research Center, Analytics Practice Area, RTI International, Research Triangle Park, NC, USA

**Keywords:** Illumina EPIC array, Epigenomics, Secondhand smoke exposure, Dietary intake, Sex difference, Smoking cessation

## Abstract

**Background:**

Smoking impacts DNA methylation, but data are lacking on smoking-related differential methylation by sex or dietary intake, recent smoking cessation (<1 year), persistence of differential methylation from *in utero* smoking exposure, and effects of environmental tobacco smoke (ETS).

**Methods:**

We meta-analysed data from up to 15,014 adults across 5 cohorts with DNA methylation measured in blood using Illumina's EPIC array for current smoking (2560 exposed), quit < 1 year (500 exposed), *in utero* (286 exposed), and ETS exposure (676 exposed). We also evaluated the interaction of current smoking with sex or diet (fibre, folate, and vitamin C).

**Findings:**

Using false discovery rate (FDR < 0.05), 65,857 CpGs were differentially methylated in relation to current smoking, 4025 with recent quitting, 594 with *in utero* exposure, and 6 with ETS. Most current smoking CpGs attenuated within a year of quitting. CpGs related to *in utero* exposure in adults were enriched for those previously observed in newborns. Differential methylation by current smoking at 4–71 CpGs may be modified by sex or dietary intake. Nearly half (35–50%) of differentially methylated CpGs on the 450 K array were associated with blood gene expression. Current smoking and *in utero* smoking CpGs implicated 3049 and 1067 druggable targets, including chemotherapy drugs.

**Interpretation:**

Many smoking-related methylation sites were identified with Illumina’s EPIC array. Most signals revert to levels observed in never smokers within a year of cessation. Many *in utero* smoking CpGs persist into adulthood. Smoking-related druggable targets may provide insights into cancer treatment response and shared mechanisms across smoking-related diseases.

**Funding:**

Intramural Research Program of the 10.13039/100000002National Institutes of Health, Norwegian Ministry of Health and Care Services and the Ministry of Education and Research, Chief Scientist Office of the 10.13039/100012095Scottish Government Health Directorates and the 10.13039/501100000360Scottish Funding Council, 10.13039/501100000265Medical Research Council UK and the 10.13039/100010269Wellcome Trust.

**Research in context**

**Evidence before this study**

While it is well established that smoking leads to changes in DNA methylation at specific CpG sites, several important research gaps that remain. First, it is unknown how recent quitting (quit less than 1 year) may impact DNA methylation. Second, there are few data examining whether smoking effects on DNA methylation may differ by sex and certain dietary intakes. Third, while there is evidence that changes in DNA methylation from *in utero* smoking exposure can persist to adolescence, no studies have examined whether these methylation signatures can be observed in adults. Fourth, exposure to environmental tobacco smoke (ETS) can lead to adverse health outcomes, but it is not well studied whether ETS alters DNA methylation at specific CpG sites.

**Added value of this study**

This large genome-wide methylation meta-analysis addresses these four major research gaps. We identified several thousand CpGs that smoking may impact that had not been previously reported. We also provide evidence that quit smoking less than a year ago can reverse the effects of smoking on DNA methylation. Smoking-related methylation at some CpG sites may differ by sex or dietary factors, though the small number of findings suggest that healthy dietary intakes may exert minimal protection against the epigenetic effects of smoking. We found evidence that exposure to *in utero* smoking alters DNA methylation with persistence into adulthood.

**Implications of all the available evidence**

The results from our study contribute to improving our understanding of the health effects of smoking and can be used in the future to create more robust biomarkers of smoking and *in utero* smoking exposure in adults. Pathway analyses across smoking exposures provide insights into smoking-related health outcomes that persist after quitting. Drug targets of implicated genes provide insights into treatment response and how smoking-related health outcomes are correlated.

## Introduction

Smoking causes adverse health outcomes throughout life.^1^ Alterations in DNA methylation could contribute to smoking-related disease mechanisms. In a large meta-analysis of adults, current smoking was associated with widespread differential methylation in blood using the Illumina 450 K methylation array.^2^ The Illumina 450 K array has been superseded by the more comprehensive EPIC array (∼850 K CpGs, ∼3% of all CpG sites in the human genome), which was designed to improve coverage of functionally important sites like enhancers. Despite smaller sample sizes, the few previous studies examining the impacts of current smoking on DNA methylation using this more comprehensive EPIC array identified additional cytosine-phosphate-guanine sites (CpGs) that were differentially methylated.^3^^,^^4^

Among former smokers, methylation at many, but not all, of these smoking-related CpGs reverted to levels observed in non-smokers.^2^^,^^3^^,^^5^, ^6^, ^7^, ^8^ However, data remain insufficient on how quickly signals observed in current smokers degrade in the months following quitting. In long-term cohort studies, former smokers are typically defined as not smoking in the past 12 months and many formers smokers studied quit many years ago.^2^^,^^3^^,^^5^^,^^6^ We are not aware of studies that have examined smokers who quit smoking more recently (i.e., quitting within a year of sampling).

Other aspects of smoking exposure not well studied in relation to blood DNA methylation include whether smoking-related methylation differs by sex, whether changes in DNA methylation from *in utero* smoking exposure persist into adulthood, whether environmental tobacco smoke (ETS) alters DNA methylation at specific CpG sites, and whether smoking-related methylation is modified by dietary factors.

Sex differences have been identified in some smoking related health outcomes.^9^, ^10^, ^11^, ^12^ Some studies of maternal smoking during pregnancy have reported differential methylation by infant sex,^13^^,^^14^ but studies examining sex interactions for differential methylation in adults are lacking.

Limited evidence suggests that differential methylation from *in utero* exposure to maternal smoking persists into adulthood at certain CpGs,^14^^,^^15^ and epigenome-wide association studies (EWAS) using the EPIC array to examine the persistence into adulthood of differential DNA methylation related to *in utero* exposure to smoking are sparse. The ability to reliably detect signals of prenatal exposure to smoking in adults that do not reflect their own smoking history would aid the detection of long-term effects of *in utero* exposure.

ETS can lead to lung cancer and cardiovascular disease in non-smokers, as well as non-malignant respiratory illness.^16^ Whether postnatal exposure to ETS leads to differential methylation remains inconclusive. One study examined only one CpG,^17^ and another did not include a replication study.^18^

Evidence exists that maternal dietary factors (i.e., intake of folate, vitamin C) may modify newborn methylation at some smoking CpGs in either placenta^19^ or blood,^13^^,^^20^ but whether these dietary factors modify methylation differences related to current smoking in adults is unknown. Studies have reported that higher dietary fibre intake reduces adverse health outcomes among smokers,^21^^,^^22^ but we are not aware of studies that have examined whether dietary fibre may modify methylation at smoking CpGs.

Because of the importance of replication and large sample size for epigenome-wide association studies, we conducted an EWAS meta-analysis of 5 cohorts (N = 15,014) to identify blood-based differentially methylated CpGs in relation to current smoking, recency of quitting smoking, persistence of effects of *in utero* smoking exposure, and ETS using the EPIC array. We also evaluated possible differences in effects of smoking on methylation by sex. Additionally, we conducted exploratory analyses of interactions between current smoking and dietary intakes of fibre, folate, and vitamin C.

## Methods

### Study populations

We analysed data from the following studies: 1) a sub-study of the Norwegian Mother, Father, and Child Cohort Study (MoBa) called STudy of Assisted Reproductive Technology (START), 2) the Agricultural Lung Health Study (ALHS), 3 and 4) Generation Scotland (GS) which included two sub-studies GS1 and GS2, and 5) the Strong Heart Study.

Details of MoBa have been described in previous publications.^23^, ^24^, ^25^, ^26^ Briefly, START selected 978 complete MoBa mother-father-newborn trios who conceived using assisted reproductive technology and 1017 randomly selected complete mother-father-newborn trios who conceived naturally between 2000 and 2008. Blood was collected from mothers (henceforth “women”) and from fathers (henceforth, “men”) at gestational week 18. This study is focused on DNA methylation measured from whole blood and smoking exposures reported by the parents in the MoBa baseline questionnaire answered around gestational week 17.

The Agricultural Lung Health Study (ALHS) is a case–control study of asthma nested within the Agricultural Health Study (AHS) (data version P3REL201209.00) cohort of farmers from Iowa and North Carolina and their spouses. The ALHS enrolled 3301 participants from the AHS between 2009 and 2013. Details of the AHS and ALHS have been previously described.^27^^,^^28^ Methylation was measured on a subset of 2391 individuals of European ancestry based on genotyping and available in 2286 after applying quality control procedures.

GS is a family-based study comprising approximately 24,000 individuals (>99% self-reported as white Scottish) in 7000 family groups, aged 18–99 years at baseline (2006–2011).^29^^,^^30^ This study includes two sub-studies that measured DNA methylation in approximately 10,000 individuals (GS1 and GS2). GS1 comprises of 5087 related individuals. For this study, a subset of 2578 unrelated individuals was analysed. GS2 comprises of 4450 individuals unrelated to each other and unrelated to GS1 participants.

The Strong Heart Study (henceforth “Strong Heart”) is a study of 4549 American Indian adults recruited from Arizona, Oklahoma, North Dakota, and South Dakota. Strong Heart previously published epigenome-wide analyses of current smoking using EPIC array.^4^ To increase power for discovery, we included their results in our meta-analysis of current smoking.

### Ethics

START was approved by the Regional Committees for Medical and Health Research Ethics (REK) South-East (2017/1362) in Norway. The establishment of MoBa and initial data collection were based on a license from the Norwegian Data Protection Agency and an approval from the Regional Committees for Medical and Health Research Ethics. The MoBa cohort is now regulated by the Norwegian Health Registry Act. The ALHS was approved by the Institutional Review Board at the National Institutes of Health (08EN136) and its contractors. All components of GS received ethical approval from the NHS Tayside Committee on Medical Research Ethics (REC Reference Number: 05/S1401/89). All participants in MoBa, ALHS, and GS provided informed consent.

### Smoking assessment

In each study, participants were categorized, based on questionnaire data, as never smokers, former smokers, or current smokers. Information on whether former smokers quit within the past year was available in START and GS. Information on *in utero* smoking exposure was available in women in START and participants of both sexes in the ALHS. Data on ETS were available in women in START and participants of both sexes in ALHS and GS. Participants were considered exposed to ETS if they reported being exposed to passive smoke at home or exposed for at least an hour a day on average.

### DNA methylation pre-processing, quality control, and cell type proportion estimation

DNA methylation was measured in blood using Illumina's EPIC array. Each cohort applied study-specific quality control procedures and normalization on their methylation data. Details of the pre-processing and quality control of the methylation data have previously been described^5^^,^^31^^,^^32^ and are available in Additional File S1: Methods. Briefly, studies corrected for batch effects by either using random effects modeling, ComBat^33^ or adjusting for processing batch in the model. To reduce the impact of extreme outliers in the methylation data, all studies, except Strong Heart, replaced extreme outliers with winsorized values (winsorize.pct = 0.005).^34^ CpGs on the sex chromosomes were excluded. Six cell type proportions (monocyte, CD4T, CD8T, B cell, NK, and neutrophil) were estimated using the Houseman method with a reference panel.^35^, ^36^, ^37^

### Cohort-specific analyses

Epigenome-wide analyses were conducted with current smoking (versus never smoked) as the exposure and DNA methylation as the outcome, using linear regression with robust sandwich estimators. The two datasets from GS were analysed separately. START used a mixed linear regression (“nlme” package in R) to account for batch effects. Assumptions for linear regression were met.

**Fig. 1:**
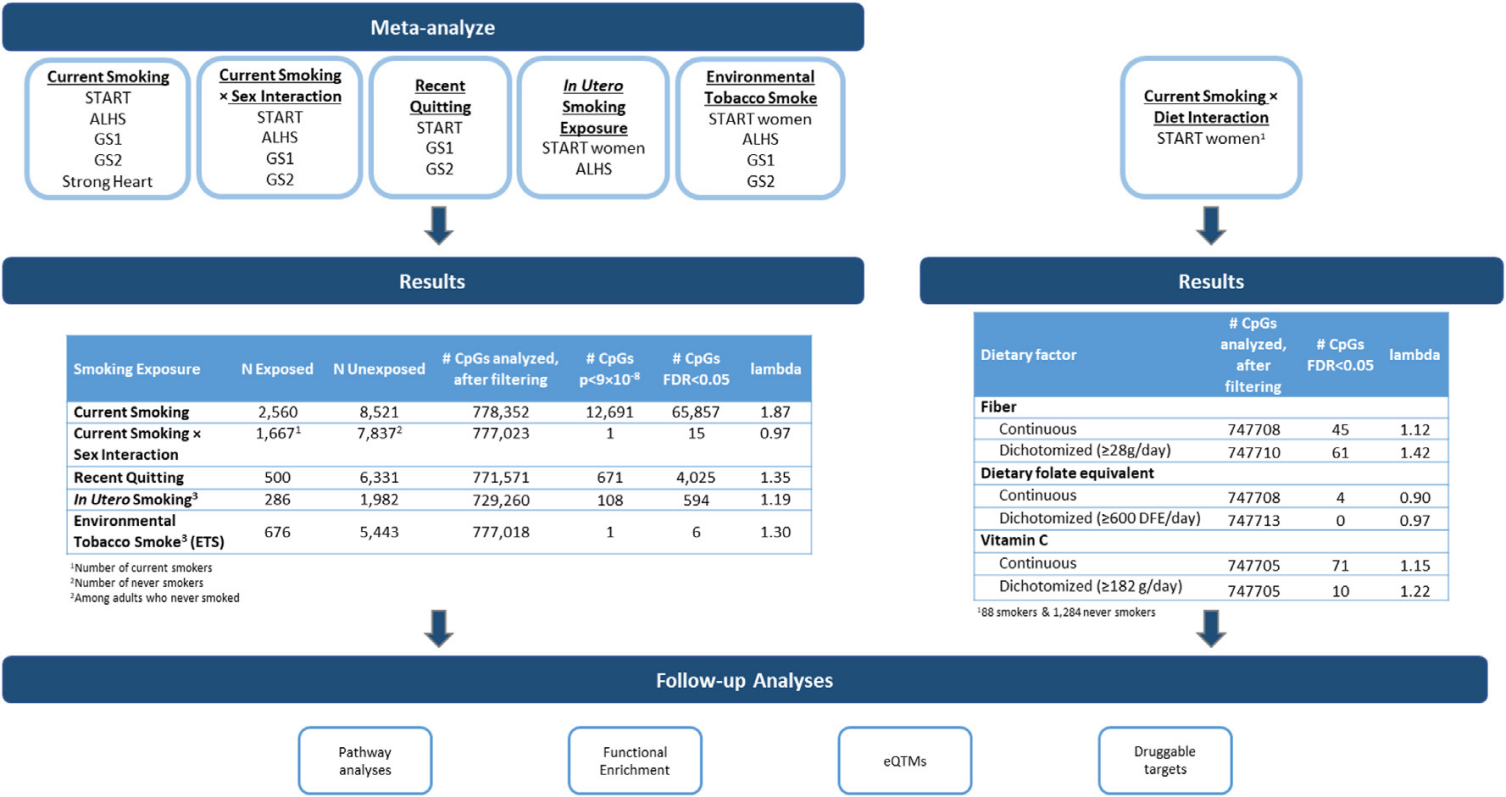
**Schematic of analyses and results.** Depicts the analyses conducted, the studies that contributed to each analysis, the results, and downstream analyses that were conducted.

Models were adjusted for age at enrolment, body mass index, sex, estimated cell type proportions (monocyte, CD4T, CD8T, B cell, NK, and neutrophil), and study-specific covariates (e.g., processing batch, phenotype for which recruitment was selected on (i.e., case/control status), highest level of completed education, state of residence at enrolment). To explore whether current smoking signals differ by sex, we repeated the analyses including an interaction product term for current smoking and sex. Analyses of differential methylation in relation to *in utero* smoking exposure (yes versus no) and ETS (yes versus no) were restricted to never smokers (Fig. 1).

### Meta-analysis and additional filtering

For current smoking, we meta-analysed 863,435 CpGs from START, ALHS, GS, and Strong Heart. The recent quitting meta-analysis included 862,776 CpGs from START and GS. The *in utero* smoking exposure meta-analysis included 836,401 CpGs from START and ALHS. The remaining meta-analyses (ETS and current smoking × sex interaction) included 863,046 CpGs from START, ALHS, and GS. Meta-analyses were conducted using fixed-effects with inverse-variance weighting in METAL.^38^^,^^39^

After meta-analysis, we removed 66,353 probes previously reported to be potentially problematic (i.e., “ch” probes, probes with a SNP in the extension base that can cause color channel switch, probes with extension base inconsistencies, and cross-reactive probes).^40^ The removal of potentially problematic probes at this stage does not affect the cohort-specific quality control procedures that were applied. We also excluded CpGs available only in one study. Significance was assessed using both a family-wise error rate (FWER) (p < 9 × 10^−8^) and Benjamini-Hochberg false discovery rate (FDR<0.05),^41^ applying a similar approach recommended for genome-wide association studies.^42^ We visualized meta-analysed results using Miami plots^43^ and study-specific results using forest plots.

### Smoking and diet interaction

Dietary information was available only for START women who completed a validated food frequency questionnaire administered in mid-pregnancy.^44^ Exploratory analyses were conducted to examine whether intake of dietary fibre, total dietary folate (from food and supplements), or vitamin C may modify the effects of smoking on DNA methylation (Fig. 1). Our focus on these three dietary factors was based on previous findings in the literature (i.e., folate and vitamin C modifying prenatal smoking exposure on infant DNA methylation^13^^,^^20^ and dietary fibre reducing adverse effects of smoking^21^^,^^22^). We excluded 35 women with estimated improbable total energy intake (KJ < 4500 or KJ > 20,000).^45^ In these analyses, we included smoking, the dietary intake, and the interaction of smoking and dietary intake and adjusted for the same covariates as previously stated, in addition to total energy. Blood draw occurred around the 18th week of gestation for all women, therefore we did not adjust for week of gestation. Because these analyses have not been conducted before, we modelled the dietary factors both continuously on the log-scale to increase power and dichotomized for clinical relevance using Institute of Medicine guidelines for pregnant women^46^: fibre ≥28 g/day and total dietary folate equivalent (DFE) ≥600 DFE/day. For vitamin C, most women consumed more than the recommended daily intake of 85 mg/day,^46^ so the median (≥182 mg/day) was used. We considered that there was evidence supporting interaction if the p-value of the interaction was smaller than the corrected p-value using FDR < 0.05.^41^

### Pathway analyses

CpGs were annotated to genes using Illumina's manifest. Pathway analyses were conducted using the “methylGSA” package in R, which accounts for probe number bias.

### Enrichment of genomic features

We used eFORGE v2.0 to identify tissue- and cell type-specific enrichment for DNase I hotspots, 15 chromatin states, and five histone marks.^47^, ^48^, ^49^ Features were compared to their distribution across the EPIC array (i.e., background”). eFORGE utilizes data from NIH Roadmap Epigenomics Mapping Consortium, which is the most comprehensive source available and measured histone marks across different tissues. The top 1000 differentially methylated CpGs (default maximum) were inputted. Transcription factor motif enrichment analyses were performed using AME, a component of the MEME Suite 5.0.5^50^ using the HOCOMOCOv11 database.^51^

### Expression quantitative trait methylation (eQTM)

We examined whether methylation at CpGs related to smoking exposures in this study correlated with gene expression. Because there are no large studies with methylation data from the EPIC array and gene expression data, we ran analyses for our FDR significant CpGs that overlapped in the 450 K array in 3075 individuals of European ancestry in the Biobank-based integrative omics study (BIOS) consortium.^52^ DNA methylation and gene expression were measured in blood. Cis-eQTM analyses were conducted for gene expression transcripts within ±250 kb of each CpG site using linear regression.

### Smoking-related druggable targets

Genes annotated to significant CpGs were linked to Uniprot IDs to search for approved or experimental (i.e., Phase 3 or 4) druggable targets in the ChEMBL database.^53^

### Role of funders

The funders did not have any role in study design, data collection, data analyses, interpretation, or writing of report.

## Results

There were 15,014 participants with eligible data: 3513 in START, 2286 in the ALHS, 6890 in GS (2501 in GS1 and 4389 in GS2), and 2325 in Strong Heart. Median ages ranged from 32.7 years in START to 62 in ALHS (Table 1). Median age was not available for Strong Heart, but 71% of participants were at least 50 years old.^4^ The proportion of current smokers ranged from 4% in ALHS to 38% reported for Strong Heart where 29% were never smokers.^4^ Additional characteristics of START, ALHS, and GS can be found in Table 1.

**Table 1:** Characteristics of the study population in START, ALHS, and GS.

	START (n = 3513)	ALHS (n = 2286)	GS1 (n = 2501)	GS2 (4389)
	N (%)	N (%)	N (%)	N (%)
Adult smoking				
Current smoker	429 (12.2)	96 (4.2)	456 (18.2)	686 (15.6)
Quit smoking	319 (9.1)	667 (29.2)	780 (31.2)	1419 (32.3)
Quit < 1 year	319 (9.1)	–	66 (2.6)	115 (2.6)
Quit ≥ 1 year	–	667 (29.2)	714 (28.5)	1304 (29.7)
Never smoked	2765 (78.7)	1523 (66.6)	1265 (50.6)	2284 (52.0)
*In Utero* smoking exposure^a^				
Yes	174	112	–	–
No	641	1341	–	–
ETS exposure^a^				
Yes	242	267	65	102
No	1120	1255	1087	1981
Fibre				
≥28 g/day	1920 (54.7)	–	–	–
<28 g/day	1412 (40.2)	–	–	–
Missing	181 (5.2)			
Folate				
≥600 DFE/day	1741 (49.6)	–	–	–
<600 DFE/day	1553 (44.2)	–	–	–
Missing	219 (6.2)			
Vitamin C				
≥182 g/day	1655 (47.1)			
<182 g/day	1639 (46.7)			
Missing	219 (6.2)			
Selection factor^b^				
Yes	1656 (47.1)	944 (41.3)	–	–
No	1857 (52.9)	1342 (58.7)	–	–
Sex				
Male	1862 (53.0)	1173 (51.3)	960 (38.4)	1924 (43.8)
Female	1651 (47.0)	1113 (48.7)	1541 (61.6)	2465 (56.2)
Education				
Less than high school	254 (7.2)	–	175 (8.0)	265 (6.8)
High school	1050 (29.9)	–	289 (13.1)	517 (13.3)
Some college	1221 (34.8)	–	421 (19.1)	770 (19.8)
College and higher	844 (24)	–	738 (33.6)	1369 (35.1)
Other	144 (4.1)	–	576 (26.2)	974 (25.0)
Missing	–	–	302	494
State				
Iowa	–	1634 (71.5)	–	–
North Carolina	–	652 (28.5)	–	–
	Median (IQR)	Median (IQR)	Median (IQR)	Median (IQR)

Age	32.7 (29.5–36.1)	62 (54–71)	52.3 (43.1–58.3)	52.9 (42.9–61.3)
Body mass index (kg/m^2^)	24.8 (22.6–27.4)	29.4 (26.0–33.6)	26.4 (23.5–30.0)	26.0 (23.3–29.3)
Cell type				
Monocyte	0.07 (0.05–0.08)	0.08 (0.06–0.10)	0.09 (0.08–0.11)	0.09 (0.08–0.11)
CD4T	0.15 (0.11–0.20)	0.16 (0.12–0.21)	0.15 (0.12–0.18)	0.15 (0.12–0.19)
CD8T	0.10 (0.07–0.13)	0.06 (0.03–0.10)	0.01 (0–0.04)	0.03 (0.01–0.06)
B cell	0.05 (0.04–0.07)	0.05 (0.03–0.07)	0.05 (0.04–0.06)	0.04 (0.03–0.06)
NK	0.06 (0.04–0.08)	0.06 (0.03–0.09)	0.08 (0.06–0.10)	0.08 (0.06–0.10)
Neutrophil	0.60 (0.49–0.71)	0.58 (0.50–0.65)	0.61 (0.56–0.65)	0.59 (0.54–0.63)
#CpGs before filtering	770,577	817,235	860,926	773,845

a Restricted to individuals who identified as never smokers. START *in utero* smoking exposure also restricted to women.

b START’s selection factor is assisted reproductive technology status; ALHS’s selection factor is asthma status.

### Meta-analysis of current smoking and DNA methylation

**Fig. 2:**
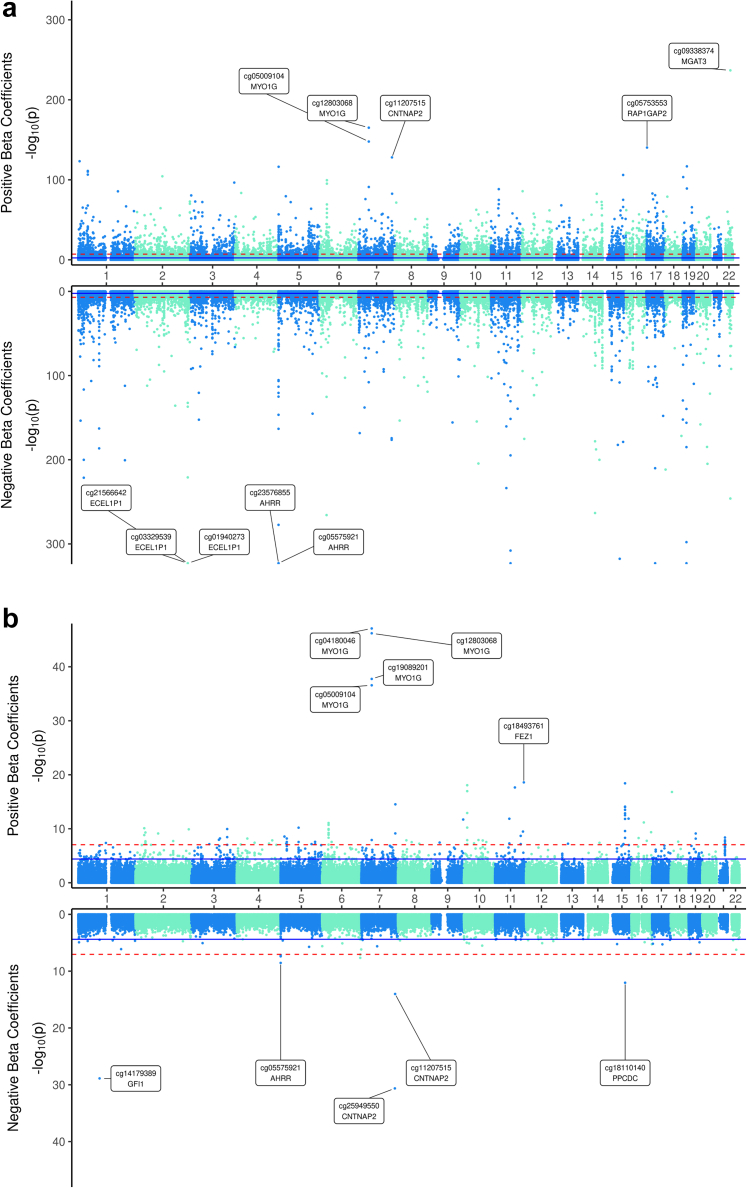
**Miami plot of meta-analysed results for A) current smoking (2560 exposed vs 8521 unexposed) and B) *in utero* smoking exposure (286 exposed vs 1982 unexposed).** In each Miami plot, the top portion of the graph shows the −log_10_ p-value of all CpGs with a positive effect estimate. The bottom portion of the graph shows the −log_10_ p-value of all CpGs with an inverse effect estimate. The top five CpGs with higher (top) or lower (bottom) differential methylation are annotated. Blue horizontal line is the FWER threshold (p = 9E-08) and the dashed line is the FDR threshold.

Meta-analysing results from START, ALHS, GS, and Strong Heart, we compared 2560 current smokers to 8521 never smokers. There were 12,691 CpGs significant at FWER (p < 9 × 10^−8^) and 65,857 CpGs significant at FDR < 0.05 (Figs. 1 and 2A, Additional File 2: Supplementary Table S1). The 12,691 FWER significant CpGs implicated 4673 unique genes; current smoking was associated with lower methylation at 7045 (55.5%) CpGs compared to never smokers (median absolute difference of 0.005 between smokers and nonsmokers, IQR: 0.004–0.008). Of the FWER significant CpGs, 5394 overlapped with CpGs on the 450 K, implicating 2557 unique genes; the remaining 7297 CpGs (57.5%) were unique to the EPIC array, implicating an additional 2115 genes. Compared to the largest meta-analysed EWAS of current smoking using the 450 K (2433 current smokers)^2^ and a smaller meta-analysis using the EPIC array (269 current smokers),^3^ our meta-analysis identified 1405 smoking-related genes not previously reported. The top 25 CpGs unique to the EPIC array are presented in Table 2. In keeping with the large number of expected findings, the genomic inflation factor (ʎ) was 1.87 (Additional File 1: Supplementary Fig. S1), which is within range of previous epigenome-wide association studies of smoking.^54^^,^^55^

**Table 2:** Top 25 CpGs differentially methylated in relation to current smoking unique to EPIC array.^a^

CpG	Chr	Position	Beta	SE	p	FDR	Direction^b^	Gene name
cg14391737	11	86,513,429	−0.062	0.0016	<1.14E-305	<7.39E-301	−−−−−	*PRSS23*
cg17739917	17	38,477,572	−0.067	0.0012	<1.14E-305	<7.39E-301	−−−−−	*RARA*
cg00475490	11	86,517,110	−0.025	0.0007	1.14E-305	7.39E-301	−−−−−	*PRSS23*
cg21911711	19	16,998,668	−0.034	0.0009	1.24E-298	7.42E-293	−−−−−	*F2RL3*
cg18110140	15	75,350,380	−0.049	0.0013	2.07E-293	1.46E-293	−−−−−	
cg02738868	14	74,221,164	−0.014	0.0004	4.94E-264	2.40E-259	−−−−−	*ELMSAN1*
cg00045592	1	160,714,299	−0.036	0.001	4.32E-257	1.98E-252	−−−−−	*SLAMF7*
cg05086879	22	39,861,490	−0.037	0.0011	8.06E-247	3.30E-242	−−−−−	*MGAT3*
cg09338374	22	39,888,390	0.028	0.0008	1.49E-237	5.80E-233	+++++	
cg19885130	11	68,146,832	−0.044	0.0013	2.09E-234	7.75E-230	−−−−−	*LRP5*
cg27215690	1	25,344,157	−0.023	0.0007	4.00E-222	1.42E-217	−−−−−	
cg22675726	18	3,179,889	−0.041	0.0013	2.16E-212	7.01E-208	−−−−−	*MYOM1*
cg25001882	14	78,619,077	−0.019	0.0006	1.97E-188	4.79E-184	−−−−−	
cg10765427	19	17,005,225	−0.019	0.0007	9.76E-186	2.23E-181	−−−−−	*CPAMD8*
cg24797066	20	48,407,084	−0.017	0.0006	1.66E-185	3.69E-181	−−−−−	
cg17738628	15	67,155,520	−0.018	0.0006	4.28E-183	9.25E-179	−−−−−	
cg25845814	14	74,224,613	−0.014	0.0005	8.16E-179	1.67E-174	−−−−−	*MIR4505*
cg07390844	18	72,935,911	−0.029	0.001	1.97E-172	3.65E-168	−−−−−	*TSHZ1*
cg05157376	1	92,781,750	−0.032	0.0012	1.35E-163	2.28E-159	−−−−−	*RPAP2*
cg06421013	20	19,194,143	−0.031	0.0012	9.64E-159	1.56E-154	−−−−−	*SLC24A3*
cg25197654	8	21,914,006	−0.016	0.0006	3.28E-154	4.82E-150	−−−−−	*DMTN*
cg09834951	19	1,265,877	−0.017	0.0006	5.03E-153	7.12E-149	−−−−−	
cg05009104	7	45,002,980	0.036	0.0014	1.47E-148	1.97E-144	+++++	*MYO1G*
cg05753553	17	2,689,486	0.024	0.0009	4.79E-141	6.11E-137	+++++	
cg10041129	11	117,685,550	−0.012	0.0005	4.04E-140	4.99E-136	−−−−−	

a Top 25 based on p-value. Model adjusted for age at enrolment, body mass index, sex, six estimated cell type proportions (monocyte, CD4T, CD8T, B cell, NK, and neutrophil), cohort-specific selection factors, as well as education in START and state in the ALHS. The meta-analysis included 2560 current smokers who were compared to 8521 never smokers.

b Direction of beta estimate in ALHS, START, GS1, GS2, and Strong Heart, respectively.

Because smoking might have different effects in pregnant women, we compared the epigenome-wide association analyses restricted to START women to the overall meta-analysis. In the START women, 2024 CpGs had an FDR < 0.05 in EWAS of current smoking, of which 81% had the same direction of effect and FDR < 0.05 in the overall meta-analysis.

### Meta-analysis of current smoking by sex interaction in relation to DNA methylation

Meta-analysis of the smoking × sex interaction term identified 15 autosomal CpGs with FDR < 0.05 (Fig. 1; Table 3; Additional File S3). Ten of these CpGs were also identified in the current smoking meta-analysis without the interaction term. There was no evidence of genomic inflation (λ = 0.97, Additional File 1: Supplementary Fig. S2).

**Table 3:** 15 FDR significant CpGs in meta-analysis of current smoking × sex interaction term.^a^

CpG	Chr	Position	Beta^b^	SE	p	FDR	Direction^c^	Gene name	In 450 K array
cg23256579	12	11,002,403	−0.028	0.0033	1.16E-17	8.98E-12	−−−−	*PRR4*	Yes
cg08035323	2	9,843,525	−0.019	0.0028	1.32E-11	5.12E-06	−−−−		Yes
cg27615582	12	11,002,411	−0.012	0.0019	9.12E-11	2.36E-05	−−−−	*PRR4*	Yes
cg24035363	17	34,906,848	−0.011	0.0019	5.09E-09	0.0008	−−−−	*GGNBP2*	
cg04513422	13	111,522,314	−0.016	0.0027	5.18E-09	0.0008	+−−−	*C13orf29*	Yes
cg09932507	17	47,643,410	−0.012	0.0021	1.07E-08	0.0012	−−−−	*LOC100288866*	
cg01212120	7	123,397,715	−0.005	0.0009	1.17E-08	0.0012	−−−−		
cg18842174	6	147,996,557	−0.021	0.0036	1.20E-08	0.0012	−−−−		
cg09653610	20	46,631,362	−0.007	0.0012	3.96E-08	0.0032	−−−−		
cg16032841	13	111,522,222	−0.014	0.0026	4.18E-08	0.0032	+−−−	*C13orf29*	Yes
cg11603447	3	150,446,034	−0.008	0.0014	4.59E-08	0.0032	−−−−		
cg25057461	10	130,507,759	0.004	0.0008	1.89E-07	0.0112	++++		Yes
cg26582982	3	194,742,712	0.002	0.0005	2.00E-07	0.0112	++−−		
cg03482123	16	54,964,029	−0.001	0.0002	2.03E-07	0.0112	−−+−	*IRX5*	Yes
cg18560003	1	108,577,346	0.002	0.0003	5.53E-07	0.0286	++−+		

a Model included current smoking, current smoking × sex, age at enrolment, body mass index, sex, six estimated cell type proportions (monocyte, CD4T, CD8T, B cell, NK, and neutrophil), cohort-specific selection factors, as well as education in START and state in the ALHS. Meta-analysis of 764 female smokers, 903 male smokers, 4378 females never smokers, and 3449 male never smokers.

b Current smoking was coded yes = 1, no = 0, and sex was coded female = 1, male = 0. The beta reflects the effect estimate for female smokers compared to non-smokers and male smokers.

c Direction of beta estimate in ALHS, START, GS1, and GS2, respectively.

### Meta-analysis of recently quitting smoking and DNA methylation

Meta-analysis of results for 500 individuals who quit smoking less than 12 months prior to methylation measurement compared to 6331 never smokers identified 671 CpGs significant at FWER (p < 9 × 10^−8^) and 4025 at FDR < 0.05 (Fig. 1; Additional File 1: Supplementary Figs. S3 and S4; Additional File 2: Supplementary Table S2). The 4025 CpGs annotated to 1918 genes, of which 1790 (93.3%) genes overlapped with the genes implicated with current smoking. The genomic inflation factor was 1.35.

Of the 12,691 CpGs FWER significant in the current smoking meta-analysis, 12,673 were available in the meta-analysis of quit smoking within a year (Additional File 2: Supplementary Table S3), and 1191 (9.4%) met significance at look-up replication level (p < 3.95 × 10^−6^ [0.05/12,673]), including 661 (5.2%) meeting FWER significance. Furthermore, 7283 CpGs (57.4%) replicated at a nominal level of p < 0.05 and same direction of association (p_enrichment_ < 1 × 10^−323^). Among these 7283 CpGs, the effect estimates in 75% of these probes attenuated by a median of 25.6% (IQR: 14.6%–37.6%) in those who quit within a year compared to current smokers (Additional File 2: Supplementary Table S3). Of the 1825 CpGs without attenuation, 1061 (58.1%) were unique to the EPIC array.

### Meta-analysis of *in utero* smoking exposure and DNA methylation

Restricting to never smokers, we compared 286 adults with exposure *in utero* to 1982 unexposed and found 108 CpGs significant at FWER (p < 9 × 10^−8^) and 594 at FDR < 0.05 (Figs. 1 and 2B; Additional File 2: Supplementary Table S4; Additional File S4). Methylation was higher in those exposed to *in utero* smoking at 545 (91.8%) CpGs. The median absolute difference was 0.015 (IQR: 0.01–0.02). There was minimal evidence of genomic inflation (λ = 1.19, Additional File 1: Supplementary Fig. S5). The 594 FDR CpGs annotated to 280 genes including 42 that do not overlap with genes implicated in our meta-analysis of current smoking, such as *NKAPL* (9 CpGs), *GABRG1* (5 CpGs), and *HIST1H1A* (5 CpGs). The top 25 CpGs (based on p-value) unique to the EPIC array are presented in Table 4.

**Table 4:** Top 25 CpGs differentially methylated in relation to *in utero* smoke exposure among never smokers unique to EPIC array.^a^

CpG	Chr	Position	Beta	SE	p	FDR	Direction^b^	Gene Name
cg05009104	7	45,002,980	0.0475	0.0037	2.67E-37	4.87E-32	++	*MYO1G*
cg14391737	11	86,513,429	0.0327	0.0037	2.26E-18	1.65E-13	++	*PRSS23*
cg02858514	18	5,488,972	0.0237	0.0028	1.54E-17	9.38E-13	++	*EPB41L3*
cg05640346	7	148,038,174	0.0192	0.0024	2.98E-15	1.67E-10	++	*CNTNAP2*
cg18110140	15	75,350,380	−0.0282	0.0039	9.12E-13	3.17E-08	−	
cg18979916	6	28,226,941	0.0341	0.005	8.60E-12	2.32E-07	++	*ZKSCAN4*
cg14630801	10	14,372,155	0.0277	0.0042	6.14E-11	1.54E-06	++	*FRMD4A*
cg04198471	2	38,325,317	0.0326	0.005	7.97E-11	1.88E-06	++	
cg13997680	6	28,226,980	0.0284	0.0044	1.13E-10	2.50E-06	++	*ZKSCAN4*
cg21189356	19	30,864,709	0.0502	0.0082	7.54E-10	1.28E-05	++	*ZNF536*
cg18163683	2	38,324,984	0.0122	0.002	1.48E-09	2.35E-05	++	
cg13480228	5	16,807,017	0.0243	0.0041	2.77E-09	4.21E-05	++	*MYO10*
cg26974661	21	36,258,596	0.0606	0.0103	4.21E-09	6.03E-05	++	*RUNX1*
cg04340894	16	31,500,246	0.0189	0.0032	5.21E-09	7.03E-05	++	*SLC5A2*
cg17673841	7	45,001,924	0.0066	0.0012	1.21E-08	0.000145	++	
cg17538881	10	14,372,108	0.0054	9.00E-04	1.28E-08	0.000151	++	*FRMD4A*
cg12305845	8	36,957,694	0.0196	0.0035	1.38E-08	0.000157	++	
cg25660691	10	81,967,281	0.0264	0.0047	1.48E-08	0.000163	++	*LINC00857*
cg10037994	18	32,556,108	0.0124	0.0022	2.47E-08	0.00025	++	*MAPRE2*
cg26486466	5	150,284,616	0.0126	0.0023	2.65E-08	0.000264	++	*ZNF300*
cg08063306	9	35,406,604	0.0211	0.0038	3.61E-08	0.000329	++	*ATP8B5P*
cg26842454	1	120,439,125	0.0197	0.0036	4.25E-08	0.000369	++	*ADAM30*
cg24432832	5	24,645,212	0.0089	0.0016	5.52E-08	0.000458	++	*CDH10*
cg18630503	8	93,031,570	0.0158	0.0029	5.84E-08	0.000471	++	*RUNX1T1*
cg00881696	2	43,328,003	0.0222	0.0041	6.02E-08	0.000477	++	

a Top 25 based on p-value. Model adjusted for age at enrolment, BMI, sex, six estimated cell type proportions (monocyte, CD4T, CD8T, B cell, NK, and neutrophil), cohort-specific selection factors, as well as education in START and state in the ALHS. Meta-analysis of the 2268 never smokers included 286 exposed *in utero* and 1982 unexposed.

b Direction of beta estimates in START and ALHS, respectively.

### Meta-analysis of environmental tobacco smoke (ETS) and DNA methylation

Restricting to never smoking adults, we compared 509 with ETS exposure to 2375 unexposed. Six CpGs were significant at FWER (p < 9 × 10^−8^) or FDR < 0.05 (λ = 1.30, Additional File 1: Supplementary Fig. S6), and methylation was lower with exposure to ETS (Fig. 1, Table 5, Additional File S5, Miami plot in Additional File 1: Supplementary Fig. S7).

**Table 5:** Six FDR significant CpGs associated with environmental tobacco smoke exposure, among never smokers.^a^

CpG	Chr	Position	Beta	SE	p	FDR	Direction^b^	Gene name	In 450 K array
cg26697320	7	26,437,681	−0.0065	0.0011	2.88E-09	0.0022	−		Yes
cg20562586	2	182,269,967	−0.002	0.0004	1.01E-07	0.0301	−−−+		Yes
cg27647038	13	78,520,478	−0.0016	0.0003	1.16E-07	0.0301	−−+−	*EDNRB*	
cg06987255	3	142,935,287	−0.0012	0.0002	2.03E-07	0.0394	−		
cg17669497	10	89,945,974	−0.002	0.0004	3.50E-07	0.0475	−		Yes
cg01678383	3	187,903,164	−0.0012	0.0002	3.67E-07	0.0475	−	*LPP*	Yes

a Analyses restricted to participants who never smoked. Model adjusted for age at enrolment, BMI, sex, six estimated cell type proportions (monocyte, CD4T, CD8T, B cell, NK, and neutrophil), cohort-specific selection factors, as well as education in START and state in the ALHS. Meta-analysis of 2884 never smokers included 509 exposed to ETS compared to 2375 not exposed.

b Direction of beta estimate in ALHS, START, GS1, and GS2, respectively.

### Interaction between current smoking and diet in relation to DNA methylation in pregnant women

We conducted exploratory interaction analysis of current smoking with dietary factors in 1372 START women (Fig. 1; Additional File 2: Supplementary Table S5). Dietary fibre was weakly correlated with dietary folate equivalent (DFE) (r_Pearson_ = 0.25) and moderately correlated with vitamin C (r_Pearson_ = 0.49). DFE was moderately correlated with vitamin C (r_Pearson_ = 0.43). For dietary fibre (g/day), 99 unique CpGs had an interaction term with FDR < 0.05 (45 from the continuous variable, λ = 1.12; 61 from the dichotomized variable, λ = 1.42). The lowest number of significant interaction terms was observed with DFE – four CpGs with an interaction term FDR < 0.05 (4 from the continuous variable, λ = 0.90; 0 from the dichotomized variable, λ = 0.97). For vitamin C, 77 unique CpGs had an interaction term FDR < 0.05 (71 from the continuous variable, λ = 1.15; 10 from the dichotomized, λ = 1.22).

### Pathway analysis

**Fig. 3:**
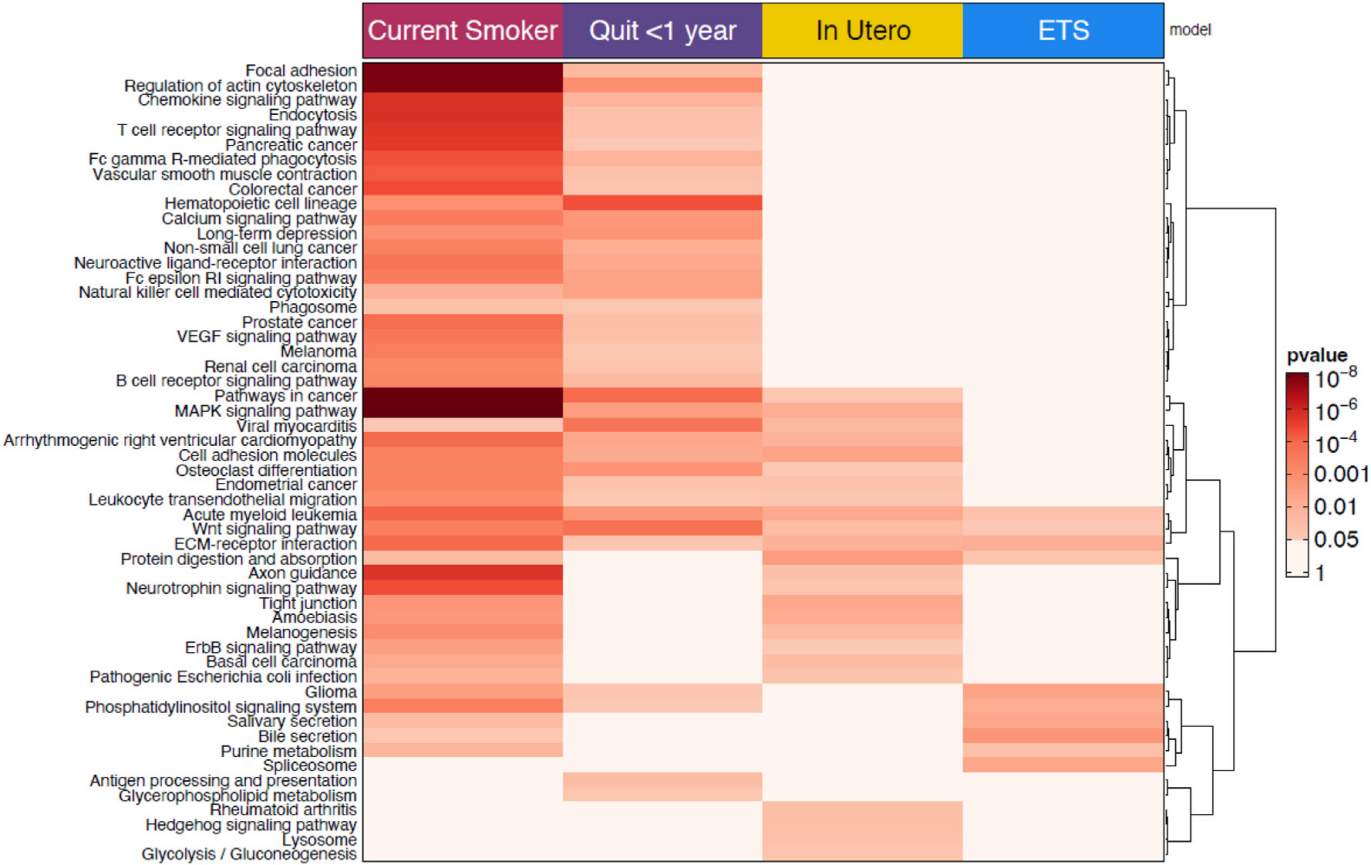
**Heatmap of enriched pathways for implicated genes from current smoking, recent quitting, in utero smoking exposure, and environmental tobacco smoke exposure models.** Column indicates the smoking model. Rows are the specific pathways. Darker shade of red means more significant enrichment.

Seventy-seven pathways were enriched for current smoking implicated genes (FDR < 0.05). Given the better power of the current smoking analysis, many more pathways were significantly enriched than for other smoking exposures. Highly enriched pathways for current smoking include MAPK signalling, pathways in cancer, focal adhesion, regulation of actin cytoskeleton, and chemokine signalling pathway (Additional File 2: Supplementary Table S6). Despite the lower power of the recent quitting analysis, hematopoietic cell lineage was more highly enriched than for current smoking (Additional File 2: Supplementary Table S7). For *in utero* smoking exposure and ETS, findings were not enriched for any pathways at FDR < 0.05 (Additional File 2: Supplementary Tables S8 and S9). Because the meta-analyses contained different sample sizes and thus power, we created a heatmap using the nominal p-value for comparison (Fig. 3). Most of the pathways with a nominal p < 0.05 for *in utero* smoking and ETS exposures were enriched in current smoking.

In pathway analyses for the dietary interaction models (Additional File 1: Supplementary Fig. S8), dietary fibre results were enriched for 37 pathways when modelled continuously and 17 when modelled dichotomously. The results for DFE were enriched for 25 and 16 pathways for the continuous and dichotomous models, respectively. For vitamin C, 16 pathways were enriched based on results from the continuous model and 12 pathways enriched in the dichotomized model.

### Enrichment of genomic features

No enrichment analyses were conducted for ETS and smoking × diet interactions due to ≤100 FDR significant CpGs. Because the epigenome-wide results for recent quitting were largely similar to current smoking but attenuated, we focused on the findings with current smoking. For current smoking (Additional File 6: Supplementary Fig. A), eFORGE identified enrichment for DNase I hotspots in blood, including hematopoietic progenitor cells. Examination of the 15 chromatin states showed significant enrichment for enhancers and transcription start sites in blood (Additional File 6: Supplementary Fig. B). In embryonic stem cells and induced pluripotent stem cells, eFORGE identified enrichment for enhancers but not transcription start sites. The top 1000 current smoking CpGs were also enriched in blood histone marks H3K4me1 (enhancer-associated) and H3K4me3 (promoter-associated). H3K4me1 but not H3K4me3 was also enriched in embryonic stem cells and induced pluripotent stem cells (Additional File 6: Supplementary Fig. C). Across DNase I hotspots, chromatin states, and histone marks, the most significant enrichments were observed in hematopoietic progenitor cells.

For *in utero* smoking exposure, eFORGE identified enrichment for DNase I hotspots in several tissues, including embryonic stem cell and induced pluripotent stem cells, but not blood (Additional File 7: Supplementary Fig. A). There was also enrichment for several tissue-specific chromatin states, including flanking active transcription start site in embryonic stem cells, mesenchymal cells, and epithelial cells (Additional File 7: Supplementary Fig. B). With histone marks, we identified enrichment for H3K27me3, H3K4me1, and H3K4me3 in embryonic stem cells, foetal lung, and induced pluripotent stem cells (Additional File 7: Supplementary Fig. C). Generally, H3K27me3 and H3K4me1 were enriched in blood but not H3K4me3.

Because there was enrichment of transcription factor binding sites for current smoking, recent quitting, and *in utero* exposure, we used the HOCOMOCOv11 database to identify enriched transcription factor motifs. For current smoking and recent quitting, many of the top motifs implicated the Erythroblast Transformation Specific (e.g., *ETV6*, *ETV4*, *ETV7*) and RUNX (e.g., *RUNX1*, *RUNX3*) family of transcription factors (Additional File 2: Supplementary Table S10). The FDR significant CpGs with *in utero* exposure were enriched for transcription factor motifs associated with TFDP1 and the E2F family (e.g., *E2F4*, *E2F1*) (Additional File 2: Supplementary Table S11).

### Expression quantitative trait methylation (eQTM)

We conducted cis-eQTM analyses in the BIOS consortium for CpGs that overlapped with the 450 K array and thus are available in BIOS (Additional File 2, Supplementary Table S12). For current smoking, of the 30,894 overlapping CpGs, 14,667 (47%) were associated with nearby gene expression (Additional File 2, Supplementary Table S13). Of the 1657 CpGs from the recent quitting model available in BIOS, 46% were associated with nearby gene expression (Additional File 2, Supplementary Table S14). From the current smoking with sex interaction model, seven CpGs overlapped in BIOS and three were significant cis-eQTMs (Additional File 2, Supplementary Table S15). For *in utero* smoking exposure, 140 (35%) of the 399 CpGs available in BIOS were associated with nearby gene expression (Additional File 2, Supplementary Table S16). Two of the four CpGs associated with ETS were associated with nearby gene expression (Additional File 2, Supplementary Table S17).

### Smoking-related druggable targets

Current smoking FDR significant CpGs implicated 3049 unique drug compounds (Additional File 2, Supplementary Table S18). The most common drug compounds were imatinib and dasatinib, chemotherapeutic agents used to treat leukaemia. CpGs associated with *in utero* smoking exposure implicated 1067 unique drug compounds (Additional File 2, Supplementary Table S19). Again, the most frequent drug compounds were used to treat cancers (i.e., sorafenib, palbociclib).

## Discussion

We conducted a large epigenome-wide meta-analysis of smoking using the EPIC array. We identified several thousand CpGs related to current smoking in adults, implicating an additional 1405 genes from CpGs unique to the EPIC array. Although the question of whether smoking-related differential methylation differs by sex has been raised in the literature, we found limited evidence for sex interaction. While methylation at most smoking CpGs reverted to levels observed in non-smokers within less than one year after cessation, 25% of CpGs did not attenuate within one year. We identified *in utero* smoking CpGs that appear to persist into adulthood. We also found differential methylation related to ETS. We provide some preliminary evidence that dietary factors modify methylation at some smoking-related CpGs in pregnant women.

Smoking has been associated with a large number of differentially methylated CpGs across the genome.^2^ Joehanes et al. reported 18,760 FDR significant CpGs,^2^ of which 16,602 were available in our study and 9176 (48.9%) were FDR significant and had the same direction of association in our similarly powered meta-analysis. Replication was higher at the gene level. Applying the annotation used in our meta-analysis to the Joehanes et al. results,^2^ the 18,760 CpGs annotated to 8690 unique genes and 7580 (87%) overlapped with the genes implicated in our meta-analysis (Additional File 2, Supplementary Table S20). Using the more comprehensive EPIC array, we identified 34,933 differentially methylated CpGs unique to the array. Some of these CpGs implicated genes not previously identified, including *FILIP1L* (10 CpGs) and *PLA2G6* (3 CpGs). In human lung tissue, smoking has been demonstrated to downregulate *FILIP1L*, which can drive lung adenocarcinoma.^56^ In our epigenome-wide analysis, we observed that smoking was associated with lower DNA methylation in *FILIP1L* (cg15554421). Based on our eQTM results, lower DNA methylation at cg15554421 increases gene expression in blood. Another study measuring DNA methylation in lung tissue reported that smoking pack-years was associated with differential DNA methylation at five CpGs.^57^ We looked up their CpGs in our study, including one that annotated to *PLA2G6*,^57^ but none were significant. The lack of replication between smoking CpGs in lung tissue and our blood-based findings highlights the importance of tissue-specificity in epigenetic studies.^58^

There is epidemiological evidence that sex or dietary factors modify the effect of smoking on lung function, COPD, and coronary artery disease.^9^, ^10^, ^11^, ^12^ We provide preliminary evidence of smoking CpGs that might be modified by sex or diet, which may provide mechanistic insights for future studies. However, given the relatively few significant sex interactions (15 CpGs) and lack of replication between results for dietary factors modelled continuously versus dichotomously, mechanisms other than DNA methylation may better explain the modified effects. Of the 15 CpGs identified in our current smoking by sex interaction model, the top three CpGs (cg23256579 [*PRR4*], cg08035323, and cg27615582 [*PRR4*]) have been previously reported to be differentially methylated by sex^59^^,^^60^ and were associated with gene expression in our eQTM analyses. Sex differences in DNA methylation at cg23256579 and cg27615582 may be driven by testosterone.^61^ A role of *PRR4* in smoking-related disease is unclear. Further research is needed to validate our findings in mechanistic studies.

As previously reported, differentially methylated CpGs related to smoking attenuate with increasing length of cessation.^2^^,^^5^^,^^62^ Focusing on recent quitting, most smoking-related CpGs attenuate within a year of cessation. In downstream analyses, results with recent quitting were less enriched for most pathways, including pathways in cancer and non-small cell lung cancer, than current smoking. Pathway analysis also suggests that smoking might have lingering effects on hematopoietic stem cell differentiation, as CpGs identified with quitting within a year were more enriched for genes involved in hematopoietic cell lineage than those with current smoking. Because the EPIC array contains almost double the number of probes as the 450 K array, our findings implicated genes that were not identified in Joehanes et al. smoking meta-analysis,^2^ including *ADGRG1*. In mice, knockout of *ADGRG1* partially alters hematopoietic cell development and differentiation, biasing cells towards myeloid.^63^ In GWAS, a SNP annotating to *ADGRG1* has been reported to be associated with red blood cell count.^64^ Despite quitting, those who have ever smoked are at an increased risk of developing haematological malignancies^65^^,^^66^ and persistence of signals could be related to activation of cells in bone marrow by smoking.^67^

Our findings with *in utero* smoking contribute to the literature that epigenetic effects of *in utero* smoking exposure might persist into adulthood.^14^^,^^15^ An earlier meta-analysis in PACE examined the 6074 CpGs differentially methylated in newborns in relation to maternal prenatal smoking^54^ and reported that 69 CpGs were significant (p < 1 × 10^−7^) in adults. Of the 67 of these 69 CpGs available in our study, 48 were FDR significant. The 399 FDR significant CpGs from our meta-analysis of *in utero* exposure that are present on the 450 K array were highly enriched for those previously identified in newborns for maternal sustained smoking during pregnancy (180 CpGs (45%), p_enrichment_ = 2.46 × 10^−219^) (Additional File 2, Supplementary Table S21).^54^ These findings further reinforce the importance of reducing *in utero* smoking exposure.

For ETS, our study (500 exposed) identified six CpGs differentially methylated. We are only aware of one other EWAS of ETS in adults (120 exposed) which reported 7 CpGs at p < 1 × 10^−5^.^18^ Of the six CpGs identified in our study, cg26697320 replicated in the previous study (β = −0.008, p = 0.01)^18^ and was also associated with gene expression in our eQTM analyses (Additional File 2, Supplementary Table S15). Five of the 7 CpGs identified in the previous study were available in our meta-analysis only cg26874015 had a p < 0.05 in our meta-analysis and same direction of association (Additional File 2, Supplementary Table S22). These two studies highlight the relative paucity of differential methylation identified for this much weaker exposure compared to active smoking. Most likely, detection of reliable signals for differential methylation by ETS exposure would require much larger sample sizes and objective exposure assessment, like high sensitivity cotinine measurements or environmental nicotine monitors.

For current smoking, recent quitting, and *in utero* smoking exposure, the FDR significant CpGs were significantly enriched for enhancers, highlighting that the EPIC array contains functionally significant differential methylation over and above its predecessors. Comparison of functional enrichments across the different smoking exposures examined is hampered by the greater power and thus larger number of differentially methylated CpGs in the current smoking analyses. However, notably, the *in utero* smoking CpGs were enriched for DNase I hypersensitive sites in foetal brain, embryonic stem cells, and induced pluripotent stem cells but not hematopoietic progenitor cells. Conversely, the current smoking CpGs were enriched for DNase I hypersensitive sites in hematopoietic progenitor cells and mesenchymal stem cells but not induced pluripotent stem cells. The CpGs related to *in utero* smoking exposure were enriched for the E2F and TFDP1 family transcription factor motifs and were not enriched in the CpGs related to current smoking. Together, there is evidence that current and *in utero* smoking have both overlapping and different functional impacts.

Identification of genes differentially methylated by smoking can shed light on mechanisms underlying the myriad of health effects from smoking, with potential implications for treatment. Impaired pulmonary function and thus chronic obstructive pulmonary disease (COPD) was one of the earliest recognized consequences of smoking. Diabetes has been causally linked to smoking^68^ and also correlates with lower lung function,^69^ but mechanisms underlying these relationships are unknown. Using GTEx,^70^ we examined which of our smoking associated CpGs are significantly related to gene expression (eQTMs) in lung and then investigated drug targets. Two genes are targets of drugs approved for treatment of hyperglycemia: alpha glucosidase (*GAA*), targeted by miglitol, and *SLC5A2* (aka *SGLT2*), targeted by dapagliflozin. This suggests shared causal pathways in the deleterious impacts of smoking on both glucose control and lung function and the potential role for these medications in low lung function.

Imatinib and dasatinib are chemotherapy drugs to treat patients with chronic myeloid leukaemia (CML). While smoking is not an established risk factor of CML, there is evidence that CML patients who smoke have higher mortality than those who do not smoke.^71^ The implicated current smoking genes that overlap with the genes that imatinib and dasatinib target may provide mechanistic insights into why smokers with CML have poorer treatment outcomes.

We used the EWAS Toolkit^72^ to evaluate overlap of our top 1000 current smoking CpGs with epigenome-wide results of traits in the EWAS Atlas.^73^ We found enrichment for 73 health conditions and traits, including mortality, lung cancers, lung function, reduced birthweight and various metabolic traits (Additional File 2, Supplementary Table S23). Whether the extensive methylation signals for smoking mediate the causal pathway to smoking-related disease is of great interest, but from an epidemiological standpoint, the current state of the field is unable to answer this question. Because DNA methylation at smoking CpGs captures smoking exposure better than questionnaire data, mediation analyses tend to overestimate the mediated effect by smoking CpGs.^74^^,^^75^ Mechanisms underlying the reproducible, site-specific differential methylation for smoking remain unknown. Mechanistic studies are needed to robustly examine the role of DNA methylation in overlapping CpGs between smoking and disease in relevant tissues, as have been noted in prior EWAS.^76^

This study has some limitations. Other than Strong Heart, our populations are largely of European ancestry. We had meta-analysis results for 281/288 CpGs reported in Strong Heart^4^ (Additional File 2, Supplementary Table S24), and 94% were FDR significant in our meta-analysis. Joehanes et al. reported that the effect estimates were highly correlated between individuals of European versus African ancestry (Spearman ρ = 0.89).^2^ Together, this suggests that the effects of smoking are largely similar across ancestry. We dichotomized ETS at 1 h/day given the low frequency of longer exposure in our populations. As previously observed with one CpG,^17^ it is possible that differential methylation observed for ETS might occur in more highly exposed populations than are now common. We uncovered evidence that intake of fibre, DFE, and vitamin C may modify the effects of current smoking but found no data for replication. Additionally, the dietary analyses we conducted in pregnant women may not be generalizable. Finally, given our focus on less well-examined aspects of the smoking-methylation association, we did not examine amount of smoking. Despite these limitations, this study is the most comprehensive meta-analysis of different smoking exposures to date using the newer EPIC array.

Our study enhances the literature on epigenetic impacts of smoking in several ways. A major strength is substantial power in our meta-analysis to identify smoking signatures using the newer EPIC methylation array. Further, we addressed several questions that have been raised on the impacts of smoking on methylation that have not been well explored. START is unique in having a population with a relatively high proportion of smokers who recently quit—mothers and fathers during the mother's pregnancy. Pregnancy is a strong motivator for parents to quit. In most long-term cohort studies, follow-up intervals for smoking cessation often examine multiple years since quitting. In addition, the issue of possible sex differences in effects of smoking has been raised,^77^ but rarely evaluated. We did not find strong evidence that sex modifies the impact of smoking on methylation genome-wide. This provides some reassurance for interpretating the bulk of the literature that did not consider sex interaction and is informative for future analyses. Another question raised in smaller prior studies, which we examined in our meta-analysis, was whether the impacts of maternal smoking during pregnancy on offspring are modified by dietary intakes. Further, by including studies with data on smoking during the pregnancy of the mother, we were able to examine persistence of signals of prenatal exposure into adulthood.

### Conclusions

In this large meta-analysis of current smoking, recent quitting, *in utero* smoking exposure, and ETS, using Illumina's EPIC array, we identified many CpGs and genes related to current smoking and provided evidence that most differences in DNA methylation levels attenuate within less than a year of cessation. Smoking differences in DNA methylation levels among those who quit within the past year may impact haematological developmental processes. We provide further evidence of the persistence of maternal smoking CpGs into older adulthood, demonstrating that pregnancy is a vulnerable window of susceptibility that can alter DNA methylation throughout the life. Analysis of druggable targets of implicated genes provides insights into correlated health effects of smoking with potential implications for treatment.

## Contributors

TTH, SEH, and SJL designed this study. The authors who developed the cohorts included in this manuscript are provided within parenthesis: The Understanding Society (RCR), START (SEH), ALHS (SJL), Generation Scotland (KLE, REM), and BIOS Consortium (BIOS Consortium). The underlying data were verified by co-authors of each study: The Understanding Society (RCR, PMH), START (SEH, MCM, CMP, YL), ALHS (SJL, ML), Generation Scotland (KLE, REM, DLM), and BIOS (ETGK, GHK). CMP, ML, and RMW applied standard quality control procedures on the DNA methylation data in START and ALHS, respectively. ALB, IDC, MR, JDW and MCM generated data utilized in the analyses. TTH, YL, DLM, ETGK, PMH, CEB, BB, AB, and JW performed statistical analyses or generated figures. AMR, SZ, and GHK provided statistical advice. TTH and SJL wrote the initial draft of the manuscript. All authors contributed to the data interpretation and provided comments on the manuscript. All authors read and approved the final manuscript.

## Data sharing statement

Data from the Norwegian Mother, Father and Child Cohort Study and the Medical Birth Registry of Norway used in this study are managed by the national health register holders in Norway (Norwegian Institute of public health) and can be made available to researchers, provided approval from the Regional Committees for Medical and Health Research Ethics (REC), compliance with the EU General Data Protection Regulation (GDPR) and approval from the data owners. The consent given by the participants does not open for storage of data on an individual level in repositories or journals. Researchers who want access to data sets for replication should apply through helsedata.no. Access to data sets requires approval from The Regional Committee for Medical and Health Research Ethics in Norway and an agreement with MoBa. Access to the START data is available upon application to the Norwegian Institute of Public Health (NIPH). An application form in English can be found at the NIPH website (http://www.fhi.no/en/). Questions regarding the START cohort can be directed to Siri Håberg (SiriEldevik.Haberg@fhi.no). Access to the ALHS is available upon request through the Agricultural Health Study Executive committee. Interested parties will need to complete a data transfer agreement with NIEHS. Questions about the ALHS can be directed to Stephanie London (london2@niehs.nih.gov).

According to the terms of consent for GS participants, access to individual-level data (omics and phenotypes) must be reviewed by the GS Access Committee. Applications should be made to access@generationscotland.org. Guidance on the Generation Scotland Access Process and Policy can be found here: https://www.ed.ac.uk/generation-scotland/using-resources/access-to-resources.

Understanding Society data are available through the UK Data Service (https://ukdataservice.ac.uk/).

Access to the Biobank-Based Integrative Omics Studies (BIOS) is available upon request. RNA-seq, DNA methylation, sex, age and cell count data can be requested and downloaded from the European Genome-phenome Archive (EGA), accession EGAS00001001077. An application form in English can be found at the BBMRI website: https://www.bbmri.nl/acquisition-use-analyze/bios.

## Declaration of interests

DLM is a part-time employee of Optima Partners Ltd. ETGK received a grant from the Netherlands Lung Foundation. GHK received grants or contracts from ZON-MW, Vertex, Netherlands Lung Foundation, GSK, TEVA the Netherlands, and European Union; consulting fees from Astra Zeneca (money to institution); honoraria from Sanofi, Boehringer Ingelheim; and chairs the exquAlro Foundation. MCM received grants from the Research Council of Norway and European Research Council. REM is a scientific advisor to the Epigenetic Clock Development Foundation and Optima Partners. All other authors have nothing to disclose.

## Appendix A Supplementary data

Additional File S7

Supplemental Consortium Authors

## References

[1] Sherman C.B. (1991). Health effects of cigarette smoking. Clin Chest Med.

[2] Joehanes R., Just A.C., Marioni R.E. (2016). Epigenetic signatures of cigarette smoking. Circ Cardiovasc Genet.

[3] Christiansen C., Castillo-Fernandez J.E., Domingo-Relloso A. (2021). Novel DNA methylation signatures of tobacco smoking with trans-ethnic effects. Clin Epigenet.

[4] Domingo-Relloso A., Riffo-Campos A.L., Haack K. (2020). Cadmium, smoking, and human blood DNA methylation profiles in adults from the strong Heart study. Environ Health Perspect.

[5] McCartney D.L., Stevenson A.J., Hillary R.F. (2018). Epigenetic signatures of starting and stopping smoking. EBioMedicine.

[6] Guida F., Sandanger T.M., Castagne R. (2015). Dynamics of smoking-induced genome-wide methylation changes with time since smoking cessation. Hum Mol Genet.

[7] Tsaprouni L.G., Yang T.P., Bell J. (2014). Cigarette smoking reduces DNA methylation levels at multiple genomic loci but the effect is partially reversible upon cessation. Epigenetics.

[8] Wan E.S., Qiu W., Baccarelli A. (2012). Cigarette smoking behaviors and time since quitting are associated with differential DNA methylation across the human genome. Hum Mol Genet.

[9] Manfrini O., Yoon J., van der Schaar M. (2020). Sex differences in modifiable risk factors and severity of coronary artery disease. J Am Heart Assoc.

[10] Shaheen S.O., Jameson K.A., Syddall H.E. (2010). The relationship of dietary patterns with adult lung function and COPD. Eur Respir J.

[11] Sorheim I.C., Johannessen A., Gulsvik A., Bakke P.S., Silverman E.K., DeMeo D.L. (2010). Gender differences in COPD: are women more susceptible to smoking effects than men?. Thorax.

[12] Prescott E., Bjerg A.M., Andersen P.K., Lange P., Vestbo J. (1997). Gender difference in smoking effects on lung function and risk of hospitalization for COPD: results from a Danish longitudinal population study. Eur Respir J.

[13] Zhang B., Hong X., Ji H. (2018). Maternal smoking during pregnancy and cord blood DNA methylation: new insight on sex differences and effect modification by maternal folate levels. Epigenetics.

[14] Richmond R.C., Simpkin A.J., Woodward G. (2015). Prenatal exposure to maternal smoking and offspring DNA methylation across the lifecourse: findings from the Avon Longitudinal Study of Parents and Children (ALSPAC). Hum Mol Genet.

[15] Wiklund P., Karhunen V., Richmond R.C. (2019). DNA methylation links prenatal smoking exposure to later life health outcomes in offspring. Clin Epigenet.

[16] Brownson R.C., Eriksen M.P., Davis R.M., Warner K.E. (1997). Environmental tobacco smoke: health effects and policies to reduce exposure. Annu Rev Public Health.

[17] Reynolds L.M., Magid H.S., Chi G.C. (2017). Secondhand tobacco smoke exposure associations with DNA methylation of the aryl hydrocarbon receptor repressor. Nicotine Tob Res.

[18] Hulls P.M., de Vocht F., Bao Y., Relton C.L., Martin R.M., Richmond R.C. (2020). DNA methylation signature of passive smoke exposure is less pronounced than active smoking: the Understanding Society study. Environ Res.

[19] Shorey-Kendrick L.E., McEvoy C.T., O'Sullivan S.M. (2021). Impact of vitamin C supplementation on placental DNA methylation changes related to maternal smoking: association with gene expression and respiratory outcomes. Clin Epigenet.

[20] Shorey-Kendrick L.E., McEvoy C.T., Ferguson B. (2017). Vitamin C prevents offspring DNA methylation changes associated with maternal smoking in pregnancy. Am J Respir Crit Care Med.

[21] Fu Z., Shrubsole M.J., Smalley W.E., Ness R.M., Zheng W. (2014). Associations between dietary fiber and colorectal polyp risk differ by polyp type and smoking status. J Nutr.

[22] Larsson S.C., Mannisto S., Virtanen M.J., Kontto J., Albanes D., Virtamo J. (2009). Dietary fiber and fiber-rich food intake in relation to risk of stroke in male smokers. Eur J Clin Nutr.

[23] Magnus P., Birke C., Vejrup K. (2016). Cohort profile update: the Norwegian mother and Child cohort study (MoBa). Int J Epidemiol.

[24] Magnus P., Irgens L.M., Haug K. (2006). Cohort profile: the Norwegian mother and Child cohort study (MoBa). Int J Epidemiol.

[25] Paltiel L., Ronningen K.S., Meltzer H.M., Baker S.V., Hoppin J.A. (2008). Evaluation of freeze thaw cycles on stored plasma in the biobank of the Norwegian mother and Child cohort study. Cell Preserv Technol.

[26] Ronningen K.S., Paltiel L., Meltzer H.M. (2006). The biobank of the Norwegian Mother and Child Cohort Study: a resource for the next 100 years. Eur J Epidemiol.

[27] House J.S., Wyss A.B., Hoppin J.A. (2017). Early-life farm exposures and adult asthma and atopy in the Agricultural Lung Health Study. J Allergy Clin Immunol.

[28] Alavanja M.C., Sandler D.P., McMaster S.B. (1996). The agricultural health study. Environ Health Perspect.

[29] Smith B.H., Campbell A., Linksted P. (2013). Cohort Profile: Generation Scotland: Scottish Family Health Study (GS:SFHS). The study, its participants and their potential for genetic research on health and illness. Int J Epidemiol.

[30] Smith B.H., Campbell H., Blackwood D. (2006). Generation Scotland: the scottish family health study; a new resource for researching genes and heritability. BMC Med Genet.

[31] Hoang T.T., Sikdar S., Xu C.J. (2020). Epigenome-wide association study of DNA methylation and adult asthma in the Agricultural Lung Health Study. Eur Respir J.

[32] Lee Y., Haftorn K.L., Denault W.R.P. (2020). Blood-based epigenetic estimators of chronological age in human adults using DNA methylation data from the Illumina MethylationEPIC array. BMC Genom.

[33] Johnson W.E., Li C., Rabinovic A. (2007). Adjusting batch effects in microarray expression data using empirical Bayes methods. Biostatistics.

[34] Suderman M., Sharp G., Yousefi P., Kupers L. (2019). Efficient and flexible EWAS. https://rdrr.io/github/perishky/ewaff/2019.

[35] Houseman E.A., Accomando W.P., Koestler D.C. (2012). DNA methylation arrays as surrogate measures of cell mixture distribution. BMC Bioinf.

[36] Reinius L.E., Acevedo N., Joerink M. (2012). Differential DNA methylation in purified human blood cells: implications for cell lineage and studies on disease susceptibility. PLoS One.

[37] Salas L.A., Koestler D.C., Butler R.A. (2018). An optimized library for reference-based deconvolution of whole-blood biospecimens assayed using the Illumina HumanMethylationEPIC BeadArray. Genome Biol.

[38] Willer C.J., Li Y., Abecasis G.R. (2010). METAL: fast and efficient meta-analysis of genomewide association scans. Bioinformatics.

[39] Rice Jpth K., Lumley T. (2018). A re-evaluation of fixed effect(s) meta-analysis. J Roy Stat Soc.

[40] Zhou W., Laird P.W., Shen H. (2017). Comprehensive characterization, annotation and innovative use of Infinium DNA methylation BeadChip probes. Nucleic Acids Res.

[41] Benjamini Y., Hochberg Y. (1995). Controlling the false discovery rate - a practical and powerful approach to multiple testing. J Roy Stat Soc B Stat Methodol.

[42] Chen Z., Boehnke M., Wen X., Mukherjee B. (2021). Revisiting the genome-wide significance threshold for common variant GWAS. G3 (Bethesda).

[43] White J.D. (2020). Miamiplot: an R package for creating ggplot2 based miami plots. https://github.com/juliedwhite/miamiplot.

[44] Brantsaeter A.L., Haugen M., Alexander J., Meltzer H.M. (2008). Validity of a new food frequency questionnaire for pregnant women in the Norwegian Mother and Child Cohort Study (MoBa). Matern Child Nutr.

[45] Meltzer H.M., Brantsaeter A.L., Ydersbond T.A., Alexander J., Haugen M. (2008). Methodological challenges when monitoring the diet of pregnant women in a large study: experiences from the Norwegian Mother and Child Cohort Study (MoBa). Matern Child Nutr.

[46] Institute of Medicine (2006).

[47] Breeze C.E., Reynolds A.P., van Dongen J. (2019). eFORGE v2.0: updated analysis of cell type-specific signal in epigenomic data. Bioinformatics.

[48] Breeze C.E. (2022). Cell type-specific signal analysis in epigenome-wide association studies. Methods Mol Biol.

[49] Breeze C.E., Paul D.S., van Dongen J. (2016). eFORGE: a tool for identifying cell type-specific signal in epigenomic data. Cell Rep.

[50] McLeay R.C., Bailey T.L. (2010). Motif Enrichment Analysis: a unified framework and an evaluation on ChIP data. BMC Bioinf.

[51] Kulakovskiy I.V., Vorontsov I.E., Yevshin I.S. (2018). HOCOMOCO: towards a complete collection of transcription factor binding models for human and mouse via large-scale ChIP-Seq analysis. Nucleic Acids Res.

[52] Bonder M.J., Luijk R., Zhernakova D.V. (2017). Disease variants alter transcription factor levels and methylation of their binding sites. Nat Genet.

[53] Mendez D., Gaulton A., Bento A.P. (2019). ChEMBL: towards direct deposition of bioassay data. Nucleic Acids Res.

[54] Joubert B.R., Felix J.F., Yousefi P. (2016). DNA methylation in newborns and maternal smoking in pregnancy: genome-wide consortium meta-analysis. Am J Hum Genet.

[55] Everson T.M., Vives-Usano M., Seyve E. (2021). Placental DNA methylation signatures of maternal smoking during pregnancy and potential impacts on fetal growth. Nat Commun.

[56] Kwon M., Rubio G., Wang H. (2022). Smoking-associated downregulation of FILIP1L enhances lung adenocarcinoma progression through mucin production, inflammation, and fibrosis. Cancer Res Commun.

[57] Freeman J.R., Chu S., Hsu T., Huang Y.T. (2016). Epigenome-wide association study of smoking and DNA methylation in non-small cell lung neoplasms. Oncotarget.

[58] Jin Z., Liu Y. (2018). DNA methylation in human diseases. Genes Dis.

[59] Singmann P., Shem-Tov D., Wahl S. (2015). Characterization of whole-genome autosomal differences of DNA methylation between men and women. Epigenet Chromatin.

[60] Grant O.A., Wang Y., Kumari M., Zabet N.R., Schalkwyk L. (2022). Characterising sex differences of autosomal DNA methylation in whole blood using the Illumina EPIC array. Clin Epigenet.

[61] Shepherd R., Bretherton I., Pang K. (2022). Gender-affirming hormone therapy induces specific DNA methylation changes in blood. Clin Epigenet.

[62] Zeilinger S., Kuhnel B., Klopp N. (2013). Tobacco smoking leads to extensive genome-wide changes in DNA methylation. PLoS One.

[63] Maglitto A., Mariani S.A., de Pater E. (2021). Unexpected redundancy of Gpr56 and Gpr97 during hematopoietic cell development and differentiation. Blood Adv.

[64] Sakaue S., Kanai M., Tanigawa Y. (2021). A cross-population atlas of genetic associations for 220 human phenotypes. Nat Genet.

[65] Fircanis S., Merriam P., Khan N., Castillo J.J. (2014). The relation between cigarette smoking and risk of acute myeloid leukemia: an updated meta-analysis of epidemiological studies. Am J Hematol.

[66] Tong H., Hu C., Yin X., Yu M., Yang J., Jin J. (2013). A meta-analysis of the relationship between cigarette smoking and incidence of myelodysplastic syndromes. PLoS One.

[67] Bergens M.A., Pittman G.S., Thompson I.J.B. (2019). Smoking-associated AHRR demethylation in cord blood DNA: impact of CD235a+ nucleated red blood cells. Clin Epigenet.

[68] U.S. Department of Health and Human Services (2014). A report of the surgeon general.

[69] Klein O.L., Krishnan J.A., Glick S., Smith L.J. (2010). Systematic review of the association between lung function and Type 2 diabetes mellitus. Diabet Med.

[70] Oliva M., Demanelis K., Lu Y. (2023). DNA methylation QTL mapping across diverse human tissues provides molecular links between genetic variation and complex traits. Nat Genet.

[71] Lauseker M., Hasford J., Saussele S. (2017). Smokers with chronic myeloid leukemia are at a higher risk of disease progression and premature death. Cancer.

[72] Xiong Z., Yang F., Li M. (2022). EWAS Open Platform: integrated data, knowledge and toolkit for epigenome-wide association study. Nucleic Acids Res.

[73] Li M., Zou D., Li Z. (2019). EWAS Atlas: a curated knowledgebase of epigenome-wide association studies. Nucleic Acids Res.

[74] London S.J. (2019). Methylation, smoking, and reduced lung function. Eur Respir J.

[75] Valeri L., Reese S.L., Zhao S. (2017). Misclassified exposure in epigenetic mediation analyses. Does DNA methylation mediate effects of smoking on birthweight?. Epigenomics.

[76] Lee M., Huan T., McCartney D.L. (2022). Pulmonary function and blood DNA methylation: a multiancestry epigenome-wide association meta-analysis. Am J Respir Crit Care Med.

[77] Arnegard M.E., Whitten L.A., Hunter C., Clayton J.A. (2020). Sex as a biological variable: a 5-year progress report and call to action. J Womens Health (Larchmt).

