# Supplementary material for: Comprehensive evaluation of smoking exposures and their interactions on DNA methylation
Source: eBioMedicine. 2024 Jan 9;100:104956. doi: 10.1016/j.ebiom.2023.104956 (PMC10825325; doi:10.1016/j.ebiom.2023.104956)

**Methods**

**Smoking Assessment in START, ALHS, and GS**
START, ALHS, and GS collected detailed self-reported smoking information via questionnaires. In START, women also reported the father’s smoking history, and it was used to supplement the father’s own report when missing. In Strong Heart, trained nurses and medical technicians collected participants’ information on smoking.^1^

**START**

Details of MoBa have been described in previous publications.^2-5^ Briefly, MoBa is a population-based pregnancy cohort study conducted by the Norwegian Institute of Public Health, recruiting pregnant women and their partners from 1999 to 2008 throughout Norway. The women consented to participation in 41% of pregnancies. The cohort includes approximately 114,500 children, 95,200 mothers, and 75,200 fathers, of whom 95% are of European ancestry based on genotyping.^6^ The current study is based on version 12 of the quality-assured data files released for research in 2019. START selected 978 complete MoBa mother-father-newborn trios who conceived using assisted reproductive technology and 1,017 randomly selected complete mother-father-newborn trios who conceived naturally between 2000 and 2008. Blood was collected from mothers (henceforth “women”) and from fathers (henceforth, “men”) at gestational week 18. This study is focused on DNA methylation measured from whole blood and smoking exposures reported by the parents in the MoBa baseline questionnaire answered around gestational week 17.

**Definition of Environmental Tobacco Smoke (ETS)**

Women and men completed different questionnaires. Information on in utero smoking exposure (i.e., exposed to mother’s smoking when she was pregnant with the adult) and exposure to environmental tobacco smoke (ETS) was only available in women. Women in START were considered exposed to ETS if they were exposed to passive smoke at home or work for at least an hour or if the father smoked and lived with her.

**DNA methylation Pre-Processing, Quality Control, and Cell Type Proportion Estimation**

Peripheral whole blood samples were randomized onto plates. DNA was bisulfite converted using the EZ-96 DNA methylation Lightining™ MagPrep kit (Zymo Research, Irvine, USA). Methylation was assessed using Illumina’s Infinium MethylationEPIC BeadChip in four batches. The iDAT files were processed using RnBeads R package ^7^. The data were processed in four batches.

Probes with cross-hybridization, high detection p-value >0.01, or near single nucleotide polymorphisms (filtering.snp=”3”) were excluded from each batch separately. Probes excluded from one batch were removed from all batches. We excluded 44,210 probes with cross-hybridization ^8^ or high detection p-value >0.01 and 16,117 probes near single nucleotide polymorphisms (filtering.snp=”3”). We used enmix.oob to correct for background noise ^9^. Among 2,034 non-replicated samples, 18 samples with low signal intensities and deviated from principal component analysis were excluded. Data were normalized using BMIQ, using the wateRmelon R package ^10^. Thirty-four parents had a second pregnancy; thus their second samples were removed from this study. After excluding samples that failed quality control (14 women, 21 men) and 80 women with DNA methylation at delivery, there were 1,867 women and 1,940 men included in this study.

Six cell type proportions (monocyte, CD4T, CD8T, B cell, NK, and neutrophil) were estimated using the Houseman method ^11^ with the Identifying Optimal Libraries (IDOL) algorithm and the Salas et al. reference panel ^12^.

START used a mixed linear regression (“nlme” package in R) to account for batch effects and adjusted for age at enrollment, body mass index, sex, maternal education (less than high school, high school, some college, college and higher, or other), ART status, and six estimated cell type proportions (monocyte, CD4T, CD8T, B cell, NK, and neutrophil).

**ALHS**

**Definition of Environmental Tobacco Smoke (ETS)**

Participants who reported having at least one regularly smoker in the household, reported being exposed to other people’s tobacco smoke in any location an average of at least one hour per day, or reported being regularly exposed to other people’s tobacco smoke in the last 12 months were exposed to ETS.

**DNA methylation Pre-Processing, Quality Control, and Cell Type Proportion Estimation**

The DNA was bisulfite converted using the EZ-96 DNA Methylation kit (Zymo Research Corporation, Irvine, CA). Methylation was assessed for 2,391 ALHS participants using the Infinium MethylationEPIC BeadChip, following Illumina’s protocol.

Sample level quality control excluded 102 participants with either >5% of CpGs with detection p-values >1·0×10^-10^ or intensity values less than three standard deviations below the mean bisulfite control intensities and 1 participant with sex mismatch. CpGs were removed if >5% of samples had detection p-values >1·0×10^-10^ (# CpGs = 31,533). Background and dye-bias correction were done using “Relic” in ENmix ^9,13^. Data were normalized using inter-array (quantile) normalization ^9^. Probe-type bias adjustment was conducted using the “Rcp” function in ENmix ^14^. We corrected for batch effect (i.e., plate effect) using “ComBat” in sva ^15^. Extreme methylation outliers were replaced with winsorized values (winsorize.pct = 0.005) using “ewaff.handle.outliers” from the ewaff R package ^16^. A total of 2,286 participants had methylation data that passed quality control and complete information.

We estimated cell type proportions for six estimated cell types (monocytes, neutrophils, natural killer cells, B cells, CD4T+ cells, and CD8T+ cells) using the Houseman method ^11^ with the Reinius reference ^17^.

Analyses were conducted using robust linear regression with robust sandwich estimators, adjusting for age, sex, state of enrollment (Iowa or North Carolina), body mass index, asthma (the selection factor), and 6 estimated cell type proportions.

**Generation Scotland**

**Definition of Environmental Tobacco Smoke (ETS)**

Generation Scotland collected information on exposure to ETS in two versions of the questionnaire. The first version collected weekly hours of exposure as a numeric variable while the second version was an ordinal variable (e.g., 1-4hrs, 5-9hrs). Both versions were harmonized by taking the median value from each ordinal entry. Participants who reported being exposed to ETS at least 7 hours a week (i.e., averaging to one hour a day) were considered exposed.

**DNA methylation Pre-Processing, Quality Control, and Cell Type Proportion Estimation**

DNA was obtained from whole blood at baseline. DNA methylation from whole blood was assessed using the Illumina EPIC array in 5200 related individuals from Set 1, and 4583 genetically unrelated individuals from Set 2. Full details on quality control have been reported previously. Briefly probes were filtered based on three criteria: 1) outliers based on visual inspection of the log median intensity of the methylated versus unmethylated signal per array, using the ShinyMethyl user interface ^18^; 2) a beadcount <3 in more than 5% of samples; and 3) more than 5% of samples have a detection p-value >0.05, using the *pfilter* function in wateRmelon ^10^. Samples were removed if predicted sex (based on DNA methylation at the X and Y chromosomes) did not match reported sex and if >1% of CpGs had a detection p-value >0.05.

We excluded three individuals in Set 1 who answered “yes” to presence of all of 16 self-reported disease conditions in the study’s health questionnaire. Filtering for outliers (N_Set1_=80; N_Set2_=83), sex mismatches (N_Set1_=19; N_Set2_=12), non-blood samples (N_Set1_=13), and poorly detected samples was performed (NSet1=18). Further filtering was then carried out to remove non-autosomal and non-CpG sites (N=22,163), CpGs with missing values and poorly detected CpGs (N_Set1_=5910; N_Set2_=8878). To reduce the effects of unmeasured confounders in the data, 20 principal components were derived from the DNAm data, after pre-correction for age, sex and plate.

We estimated cell type proportions for six estimated cell types (monocytes, neutrophils, natural killer cells, B cells, CD4T+ cells, and CD8T+ cells) using the Houseman method ^11^ with the Reinius reference ^17^.

Analyses were conducted separately for the two Generation Scotland sets. We ran robust linear regression with robust sandwich estimators, adjusting for age, sex, education (less than high school, high school, some college, or college and higher), body mass index, 6 estimated cell type proportions, plate, and 20 methylation-derived PCs.

**Pathway Analyses**

CpGs were annotated to genes using Illumina’s manifest. Pathway analyses were conducted using the “methylGSA” package in R, using the full epigenome-wide results after filtering. We used robust rank aggregation48 to account for the probe number bias (i.e., differing number of CpGs per gene)49 and then applied the Gene Set Enrichment Analysis (GSEA)50 with the Kyoto Encyclopedia of Genes and Genomes (KEGG) database.51,52 We considered pathways with gene sets between 50 (to include the small cell lung cancer pathway) and 500 genes (the default).

**Enrichment of Genomic Features**

To identify enriched transcription factor motifs, a ±250 window was created around each CpG site. To ensure CpGs nearby each other were not overrepresented, CpGs were merged into windows. For CpGs closer than 251 nt, CpGs were merged to form a window. It was possible for a window to overlap with nearby windows, so overlapping windows were truncated. If windows were larger than 750 nt or 1500 nt, they were split into two or three windows, respectively. The HOCOMOCOv11 database (HOmo sapiens COmprehensive MOdel COllection (autosome.org) was used to determine human transcription factor motifs nearby each window ^19^. We compared the frequency of motifs near significant CpGs (i.e., true positives) and an equally sized random set of CpGs with FDR>0.5 (i.e., false positives) using MEME Suite component AME (AME - MEME Suite (meme-suite.org)) that implements a one-tailed Fisher exact test by default.

**eQTM analyses in BIOS**

We used DNA methylation and gene expression data from 3,075 samples in the Biobank-based integrative omics study (BIOS) consortium from the following four cohorts: Leiden Longevity Study, LifeLines Study, Rotterdam Study, and Netherlands Twin Register ^20^.

LifeLines is a multidisciplinary prospective population-based cohort study examining the health and health-related behaviors of 167,729 individuals living in the northern parts of the Netherlands using a unique three-generation design. It employs a broad range of investigative procedures assessing biomedical, sociodemographic, behavioral, physical and psychological factors contributing to health and disease in the general population. A subset of 1,500 LifeLines participants also take part in LLD9. For these participants, additional molecular data are generated, allowing for a more thorough investigation of the association between genetic and phenotypic variation.

Leiden Longevity Study (LLS) aims to identify genetic factors influencing longevity and examine their interaction with the environment as a means to develop interventions to increase health at older ages. To this end, long-lived siblings of European descent were recruited together with their offspring and their offspring's partners, on the condition that at least two long-lived siblings were alive at the time of ascertainment. For men, the age criterion was 89 years or older; for women, the age criterion was 91 years or older. These criteria led to the ascertainment of 944 long-lived siblings from 421 families, together with 1,671 of their offspring and 744 partners.

The Netherlands Twin Register (NTR) was established in 1987 to study the extent to which genetic and environmental influences cause phenotypic differences between individuals. To this end, data from twins and their families (nearly 200,000 participants) from all over the Netherlands are collected, with a focus on health, lifestyle, personality, brain development, cognition, mental health and aging.

The Rotterdam Study is a single-center, prospective population-based cohort study conducted in Rotterdam, the Netherlands. Subjects were included in different phases, with a total of 14,926 men and women aged 45 years and over included as of late 2008. The main objective of the Rotterdam Study is to investigate the prevalence and incidence of and risk factors for chronic diseases to contribute to better prevention and treatment of such diseases in the elderly.

Total RNA from whole blood was depleted of globin transcripts using the Ambion GLOBIN clear kit and subsequently processed for sequencing using the Illumina TruSeq version 2 library preparation kit. Paired-end sequencing of 2 × 50-bp reads was performed using the Illumina HiSeq 2000 platform, pooling ten samples per lane. Finally, read sets were generated for each sample using CASAVA, retaining only reads passing the Illumina Chastity Filter for further processing. RIN scores were assessed during quality control and samples with a low RIN score were excluded. Data were generated by the Human Genotyping facility (HugeF) of ErasmusMC, the Netherlands ^20^. The genes from RNAseq data were annotated based on GRCh 37 using R package biomaRt, which is standard for analyzing RNA-sequencing data. Raw count data were transformed to log2CPM using voom function in the limma package ^21^.

DNA methylation data were also annotated based on hg19 using Illumina annotation file for 450K array, to get the position of the CpG site. cis-eQTMs for significant CpG sites were conducted for gene expression transcripts within ± 250 kb of each CpG site by linear regression model using R package limma.

The final model used in this analysis is: Gene expression (log2CPM) ~ DNA methylation (M values) + age + sex + lymphocyte proportion + monocyte proportion + RNA flow cell number.

The model inflation was corrected using the “bacon” method. We performed cis-eQTM for each cohort and then the results were meta-analyzed using an inverse variance-weighted fixed-effects model in METAL. Correction for multiple testing based on number of CpG sites was performed using Benjamini-Hochberg.

**References**

1. Domingo-Relloso A, Riffo-Campos AL, Haack K, et al. Cadmium, Smoking, and Human Blood DNA Methylation Profiles in Adults from the Strong Heart Study. *Environ Health Perspect* 2020; **128**(6): 67005.

2. Magnus P, Birke C, Vejrup K, et al. Cohort Profile Update: The Norwegian Mother and Child Cohort Study (MoBa). *Int J Epidemiol* 2016; **45**(2): 382-8.

3. Magnus P, Irgens LM, Haug K, et al. Cohort profile: the Norwegian Mother and Child Cohort Study (MoBa). *Int J Epidemiol* 2006; **35**(5): 1146-50.

4. Paltiel L, Ronningen KS, Meltzer HM, Baker SV, Hoppin JA. Evaluation of Freeze Thaw Cycles on stored plasma in the Biobank of the Norwegian Mother and Child Cohort Study. *Cell Preserv Technol* 2008; **6**(3): 223-30.

5. Ronningen KS, Paltiel L, Meltzer HM, et al. The biobank of the Norwegian Mother and Child Cohort Study: a resource for the next 100 years. *Eur J Epidemiol* 2006; **21**(8): 619-25.

6. Corfield EC, Frei O, Shadrin AA, et al. The Norwegian Mother, Father, and Child cohort study (MoBa) genotyping data resource: MoBaPsychGen pipeline v.1. *bioRxiv* 2022.

7. Muller F, Scherer M, Assenov Y, et al. RnBeads 2.0: comprehensive analysis of DNA methylation data. *Genome Biol* 2019; **20**(1): 55.

8. McCartney DL, Walker RM, Morris SW, McIntosh AM, Porteous DJ, Evans KL. Identification of polymorphic and off-target probe binding sites on the Illumina Infinium MethylationEPIC BeadChip. *Genom Data* 2016; **9**: 22-4.

9. Xu Z, Niu L, Li L, Taylor JA. ENmix: a novel background correction method for Illumina HumanMethylation450 BeadChip. *Nucleic Acids Res* 2016; **44**(3): e20.

10. Pidsley R, CC YW, Volta M, Lunnon K, Mill J, Schalkwyk LC. A data-driven approach to preprocessing Illumina 450K methylation array data. *BMC Genomics* 2013; **14**: 293.

11. Houseman EA, Accomando WP, Koestler DC, et al. DNA methylation arrays as surrogate measures of cell mixture distribution. *BMC Bioinformatics* 2012; **13**: 86.

12. Salas LA, Koestler DC, Butler RA, et al. An optimized library for reference-based deconvolution of whole-blood biospecimens assayed using the Illumina HumanMethylationEPIC BeadArray. *Genome Biol* 2018; **19**(1): 64.

13. Xu Z, Langie SA, De Boever P, Taylor JA, Niu L. RELIC: a novel dye-bias correction method for Illumina Methylation BeadChip. *BMC Genomics* 2017; **18**(1): 4.

14. Niu L, Xu Z, Taylor JA. RCP: a novel probe design bias correction method for Illumina Methylation BeadChip. *Bioinformatics* 2016; **32**(17): 2659-63.

15. Johnson WE, Li C, Rabinovic A. Adjusting batch effects in microarray expression data using empirical Bayes methods. *Biostatistics* 2007; **8**(1): 118-27.

16. Suderman M. perishky/ewaff: Efficient and Flexible EWAS. March 22, 2022 2019. <https://rdrr.io/github/perishky/ewaff/2019>).

17. Reinius LE, Acevedo N, Joerink M, et al. Differential DNA methylation in purified human blood cells: implications for cell lineage and studies on disease susceptibility. *PLoS One* 2012; **7**(7): e41361.

18. Fortin JP, Fertig E, Hansen K. shinyMethyl: interactive quality control of Illumina 450k DNA methylation arrays in R. *F1000Res* 2014; **3**: 175.

19. Kulakovskiy IV, Vorontsov IE, Yevshin IS, et al. HOCOMOCO: towards a complete collection of transcription factor binding models for human and mouse via large-scale ChIP-Seq analysis. *Nucleic Acids Res* 2018; **46**(D1): D252-D9.

20. Bonder MJ, Luijk R, Zhernakova DV, et al. Disease variants alter transcription factor levels and methylation of their binding sites. *Nat Genet* 2017; **49**(1): 131-8.

21. Ritchie ME, Phipson B, Wu D, et al. limma powers differential expression analyses for RNA-sequencing and microarray studies. *Nucleic Acids Res* 2015; **43**(7): e47.

**Legends for Supplemental Figures**

**Additional File 1: Figure S1.** **Cohort specific and meta-analysis QQ plots and lambdas for current smoking.** QQ plot show the observed versus expected -log_10_ p-value for START, ALHS, GS1, GS2, Strong Heart, and the meta-analysis results. Lambdas are provided in the lower right quadrant of each graph. The red line has a slope of 1.

**Additional File 1: Figure S2.** **Cohort specific and meta-analysis QQ plots and lambdas for current smoking x sex interaction.** QQ plot show the observed versus expected -log_10_ p-value for START, ALHS, GS1, GS2, and the meta-analysis results. Lambdas are provided in the lower right quadrant of each graph. The red line has a slope of 1.

**Additional File 1: Figure S3. Miami plot of Meta-analysed CpGs in relation to Recent Quitting.** The top portion of the graph shows the -log_10_ p-value of all CpGs with a positive effect estimate with recent quitting (i.e., quit within the past year). The bottom portion of the graph shows the -log_10_ p-value of all CpGs with an inverse effect estimate with recent quitting. The top five CpGs with higher (top) or lower (bottom) differential methylation in relation to recent quitting are annotated. Blue horizontal line is the FWER threshold (p=9E-08) and the dashed line is the FDR threshold.

**Additional File 1: Figure S4. Cohort specific and meta-analysis QQ plots and lambdas for quit smoking less than a year.** QQ plot show the observed versus expected -log_10_ p-value for START males, START females, GS1, GS2, and the meta-analysis results. Lambdas are provided in the lower right quadrant of each graph. The red line has a slope of 1.

**Additional File 1: Figure S5. Cohort specific and meta-analysis QQ plots and lambdas for *in utero* smoking exposure.** QQ plot show the observed versus expected -log_10_ p-value for START, ALHS, and the meta-analysis results. Lambdas are provided in the lower right quadrant of each graph. The red line has a slope of 1.

**Additional File 1: Figure S6. Cohort specific and meta-analysis QQ plots and lambdas for ETS** QQ plot show the observed versus expected -log_10_ p-value for START, ALHS, GS1, GS2, and the meta-analysis results. Lambdas are provided in the lower right quadrant of each graph. The red line has a slope of 1.

**Additional File 1: Figure S7. Miami plot of Meta-analysed CpGs in relation to Environmental Tobacco Smoke.** The top portion of the graph shows the -log_10_ p-value of all CpGs with a positive effect estimate with ETS. The bottom portion of the graph shows the -log_10_ p-value of all CpGs with an inverse effect estimate with ETS. The top five CpGs with higher (top) or lower (bottom) differential methylation in relation to ETS are annotated. Blue horizontal line is the FWER threshold (p=9E-08) and the dashed line is the FDR threshold.

**Additional File 1: Figure S8. Heatmap of enriched pathways in current smoking × diet interaction models.** Column indicates the smoking model. Rows are the specific pathways. Darker shade of red means more significant enrichment. Model name ending in “c” means the dietary variable was modelled continuously. Model name ending in “d” means the dietary variable was modelled dichotomously.

**Additional File 3: Forest plot for the 15 CpGs FDR significant in the current smoking × sex interaction model.**

**Additional File 4: Forest plot for the 108 FWER significant CpGs from the *in utero* smoking exposure model.**

**Additional File 5: Forest plot for the 6 FDR significant CpGs from the ETS model.**

**Additional File 6: eFORGE results inputting the top 1000 CpGs identified from the current smoking meta-analysis.** A) Enrichment of DNase hypersensitivity sites by specific tissues. B) Enrichment of 15 chromatin states by specific tissues. C) Enrichment of histone marks by specific tissues. The specific cell types are provided on the x-axis. The -log_10_ p-value is on the y-axis.

**Additional File 7: eFORGE results inputting the top 1000 CpGs identified from the *in utero* smoking exposure meta-analysis.** A) Enrichment of DNase hypersensitivity sites by specific tissues. B) Enrichment of 15 chromatin states by specific tissues. C) Enrichment of histone marks by specific tissues. The specific cell types are provided on the x-axis. The -log_10_ p-value is on the y-axis.

**Figures**

**Figure S1. Cohort specific and meta-analysis QQ plots and lambdas for current smoking.** **
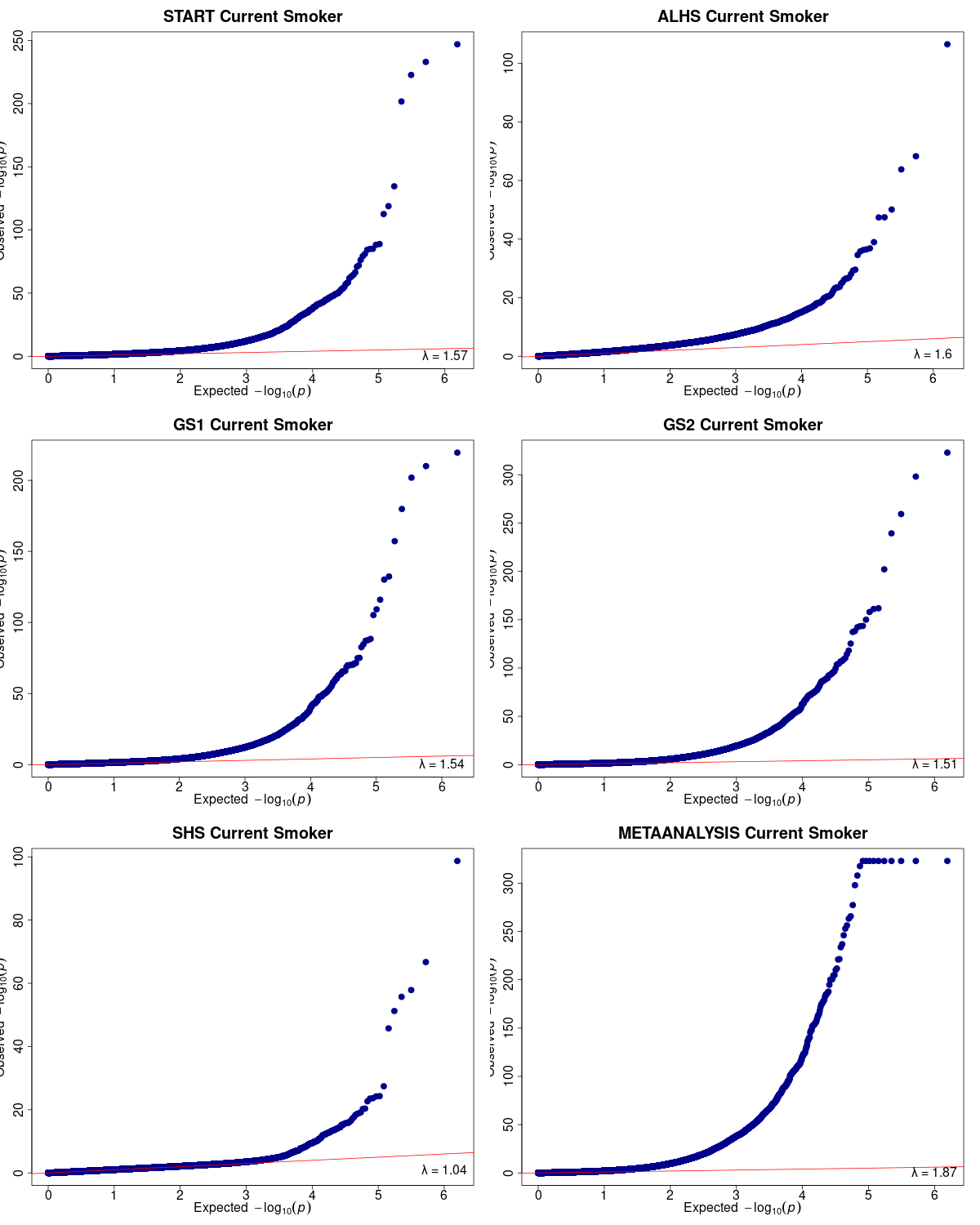
**

**Figure S2. Cohort specific and meta-analysis QQ plots and lambdas for current smoking** **× sex interaction.**

**
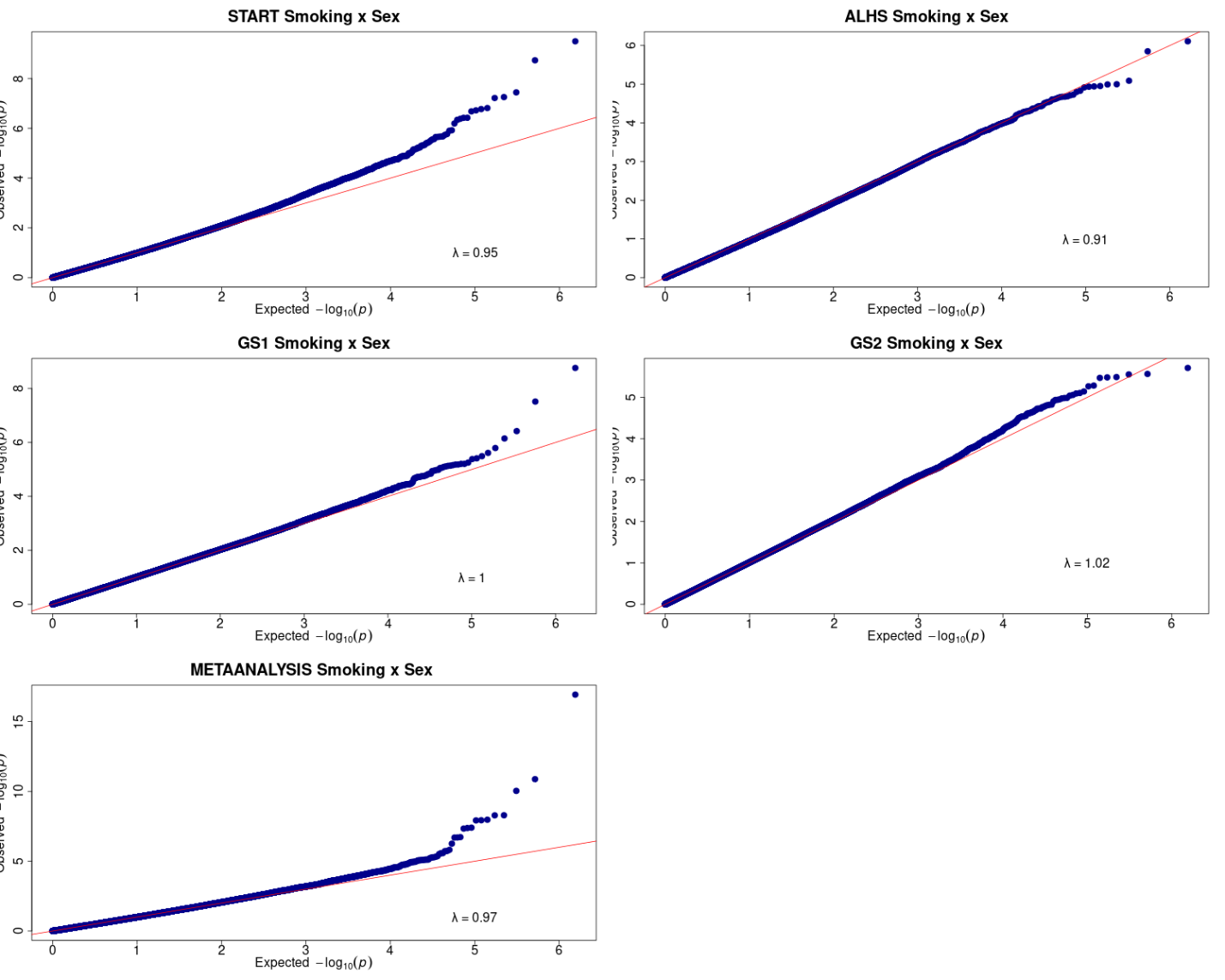
**

**Figure S3. Miami plot of Meta-analyzed CpGs in relation to Recent Quitting**

**
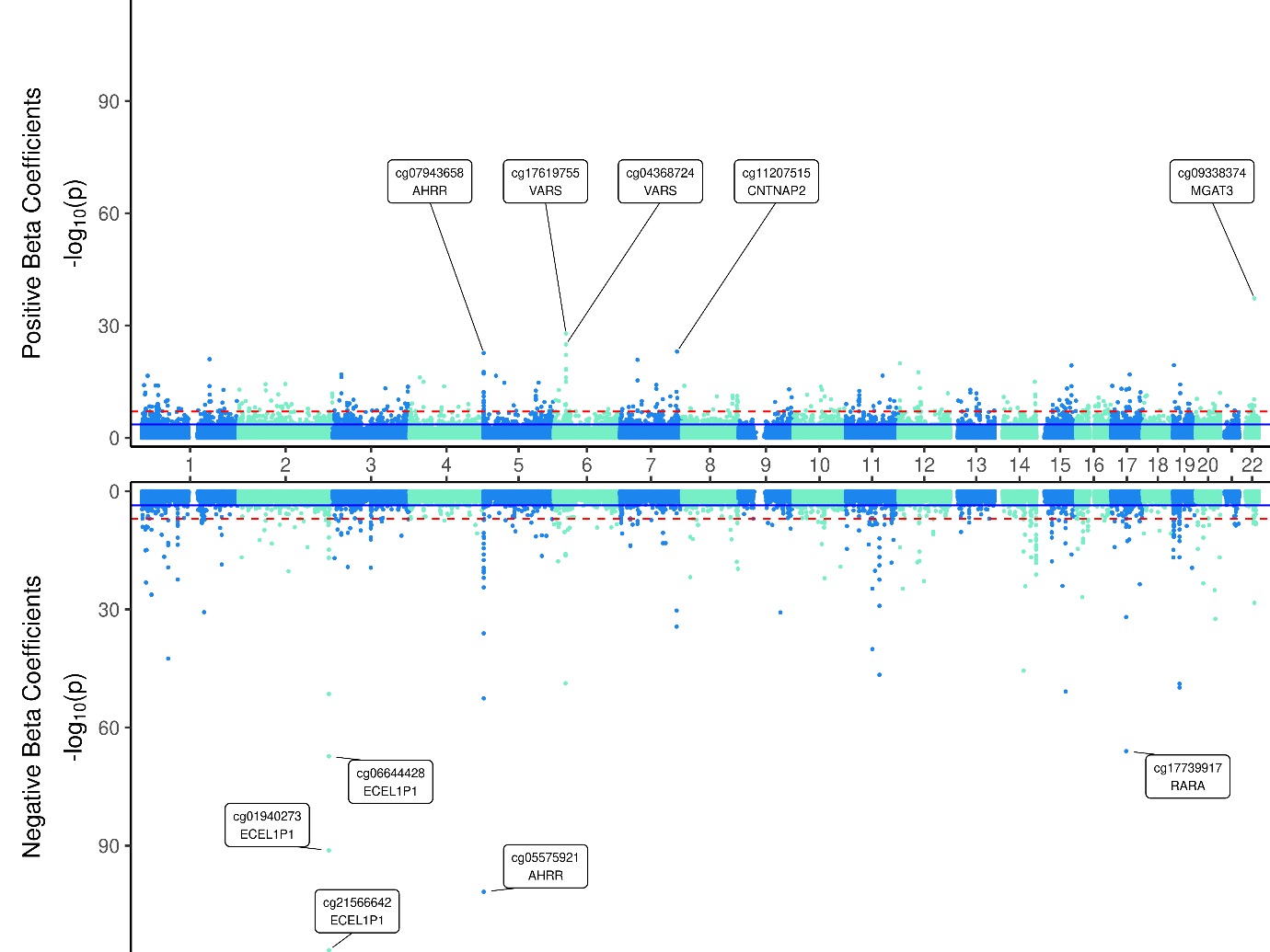
**

**Figure S4. Cohort specific and meta-analysis QQ plots and lambdas for quit smoking less than a year**

**
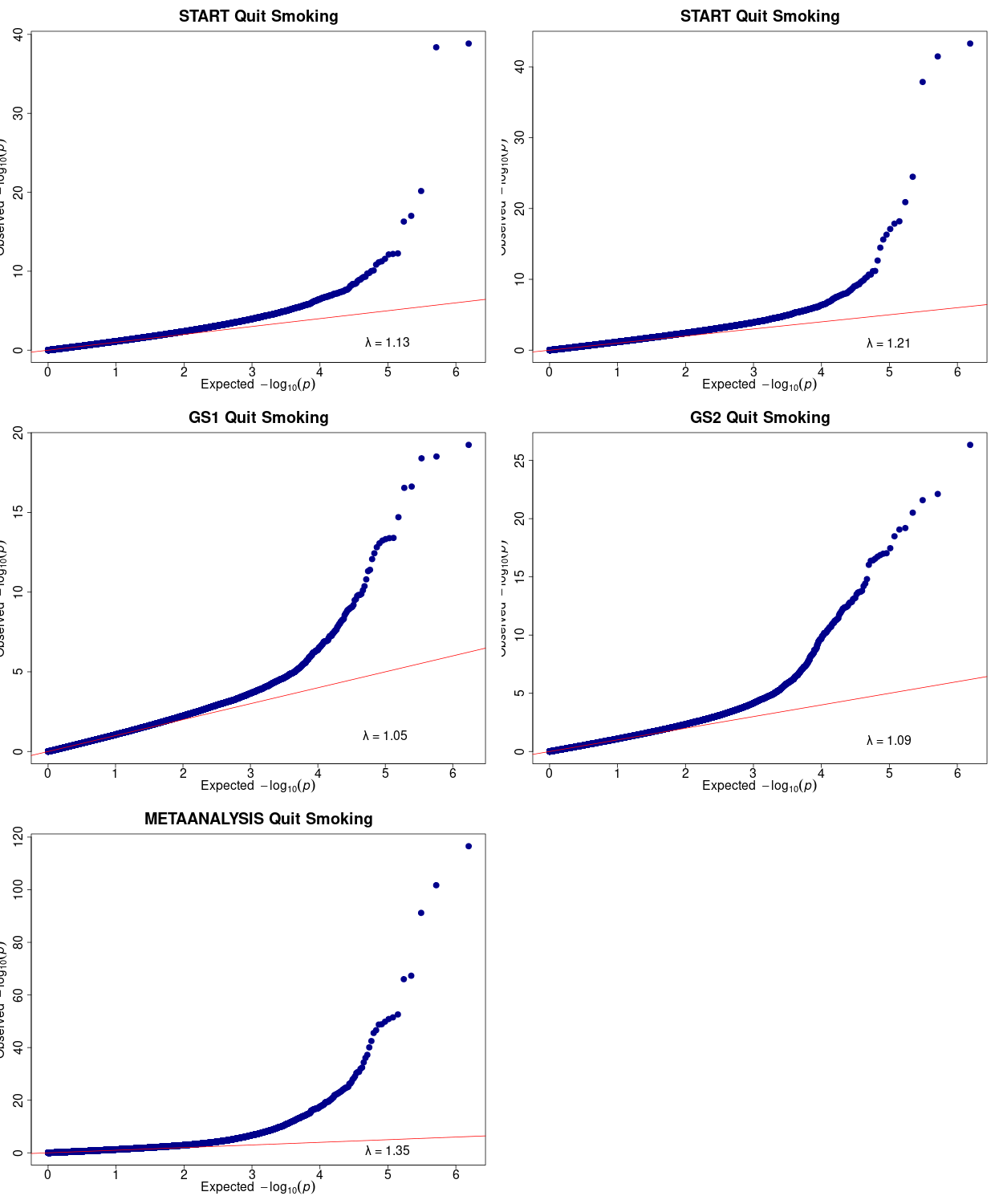
**

**Figure S5. Cohort specific and meta-analysis QQ plots and lambdas for *in utero* smoking exposure**

**
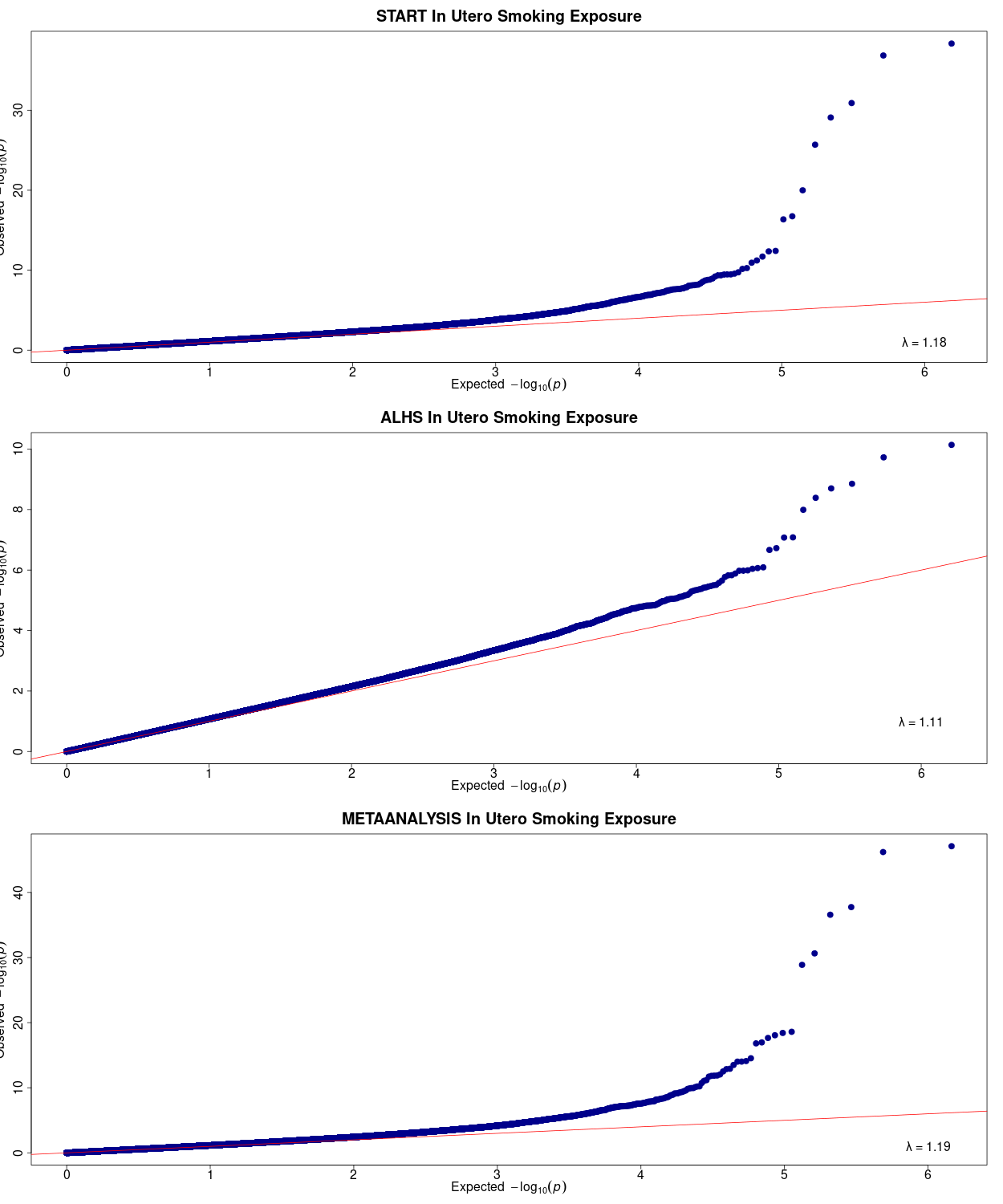
**

**Figure S6. Cohort specific and meta-analysis QQ plots and lambdas for ETS**

**
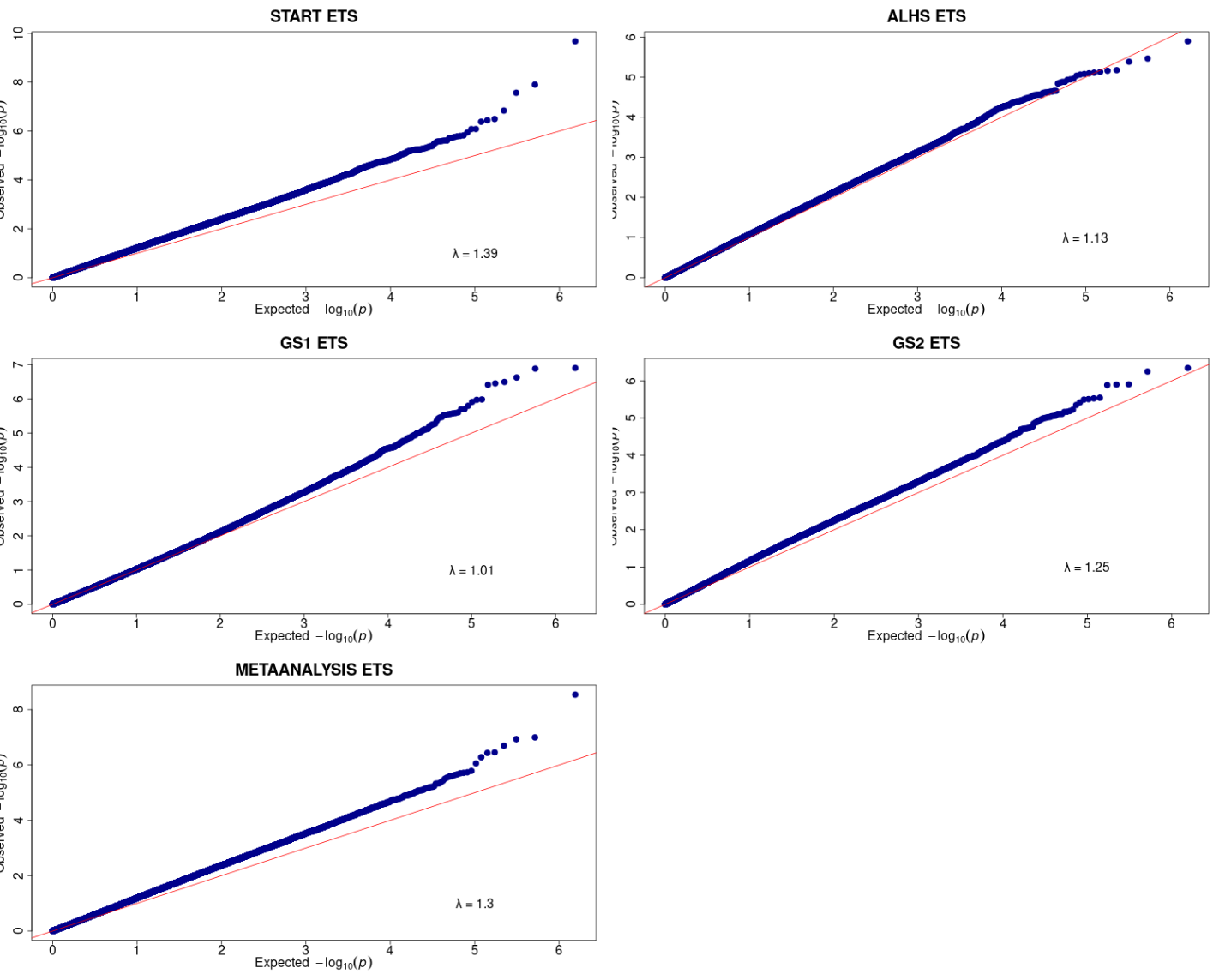
**

**Figure S7. Miami plot of Meta-analyzed CpGs in relation to Environmental Tobacco Smoke**


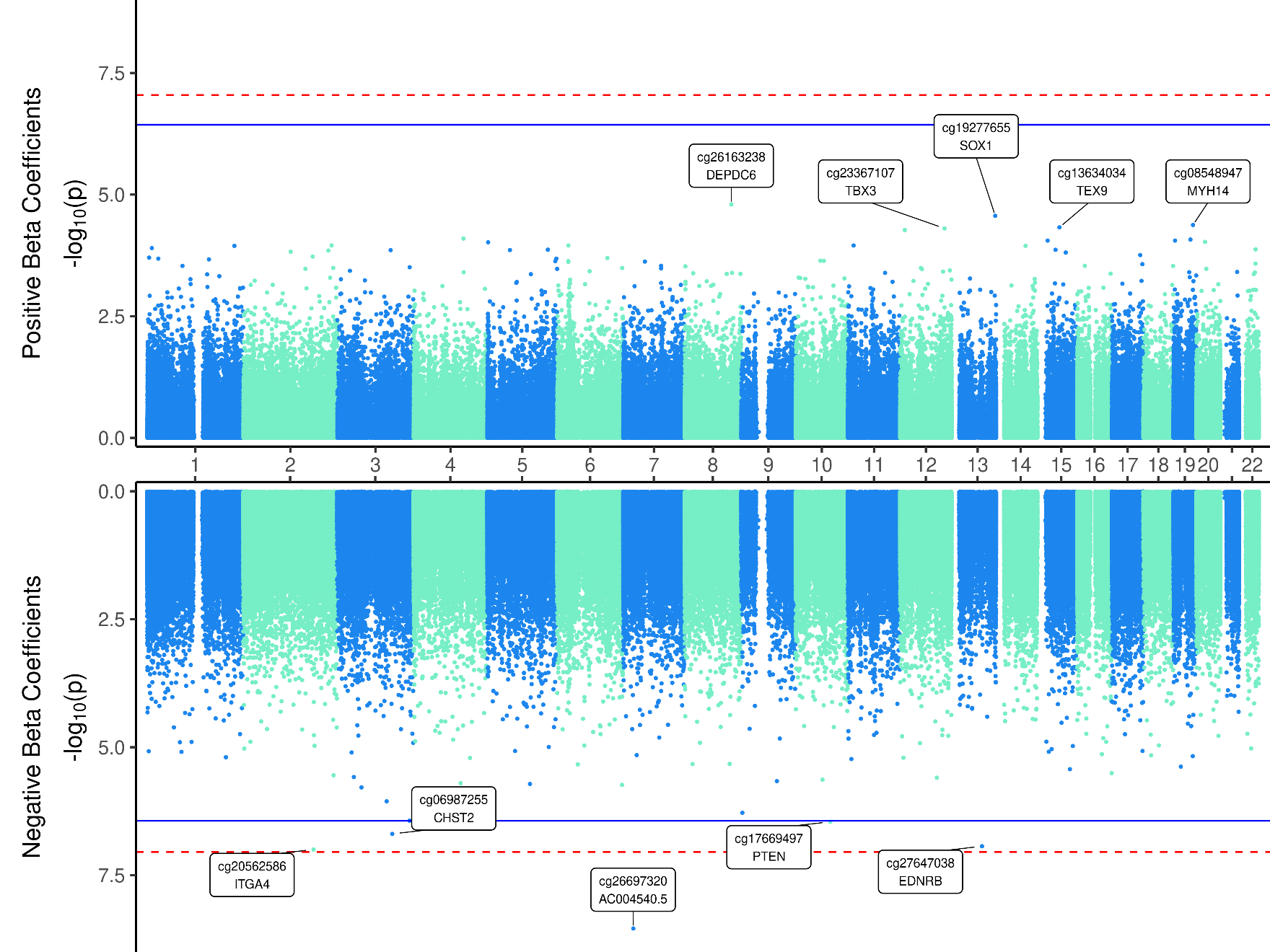


**Figure S8. Heatmap of enriched pathways in current smoking × diet interaction models**


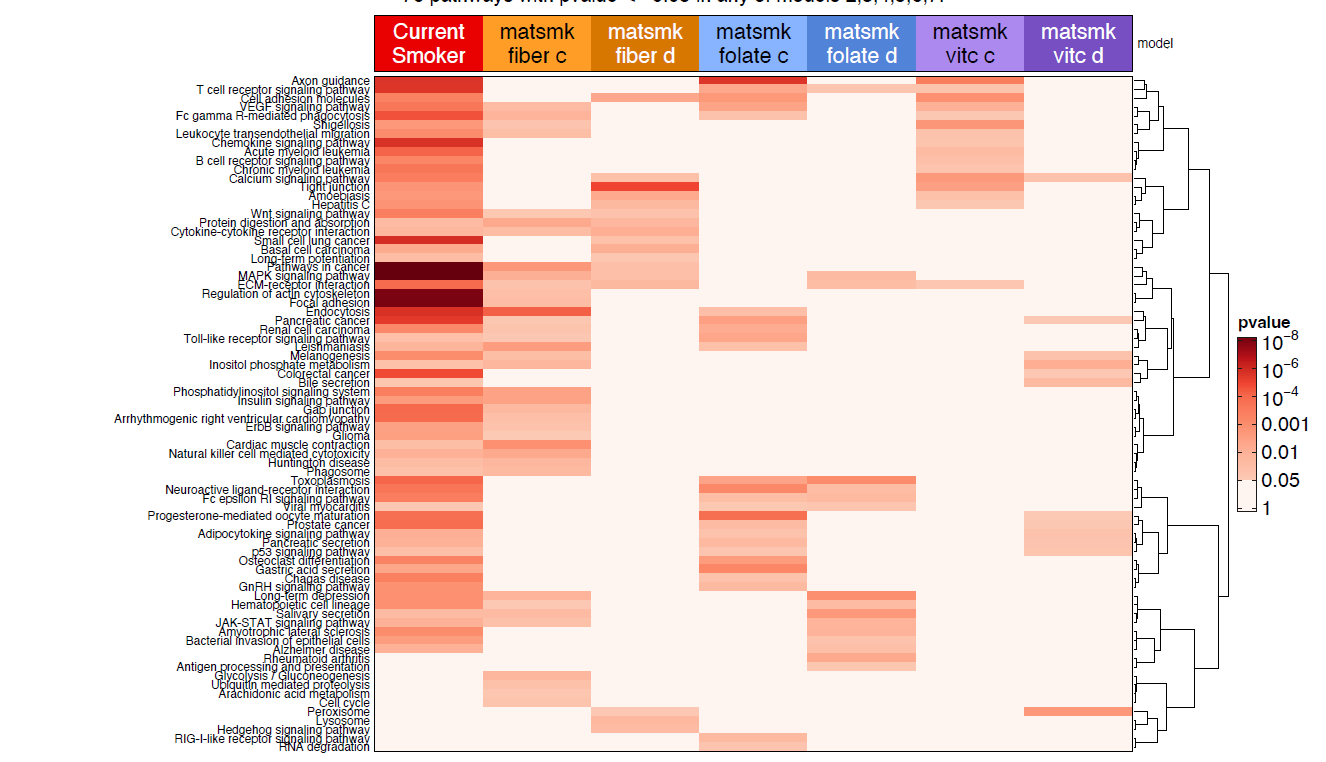

Supplement: Additional File S1 [file mmc1.docx]
