# Supplementary figures and images for: Comprehensive evaluation of smoking exposures and their interactions on DNA methylation
Source: eBioMedicine. 2024 Jan 9;100:104956. doi: 10.1016/j.ebiom.2023.104956 (PMC10825325; doi:10.1016/j.ebiom.2023.104956)

cg23256579

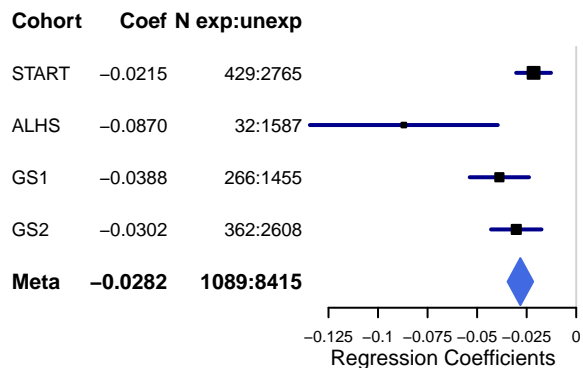

cg08035323

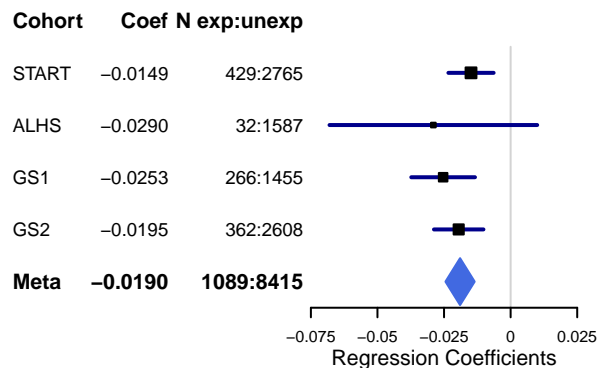

cg27615582

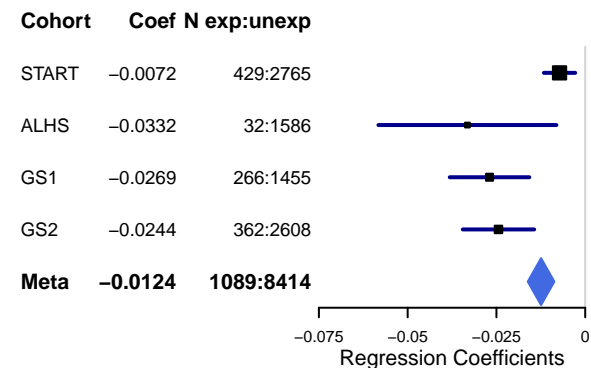

cg24035363

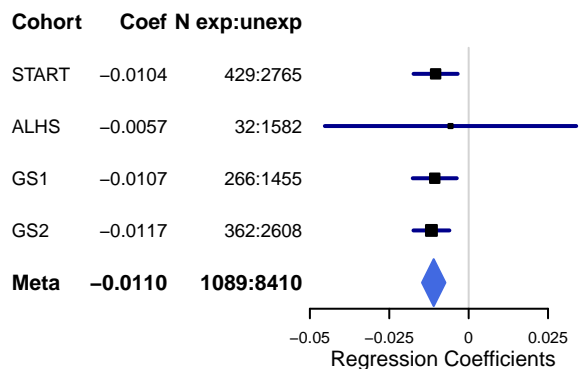

cg04513422

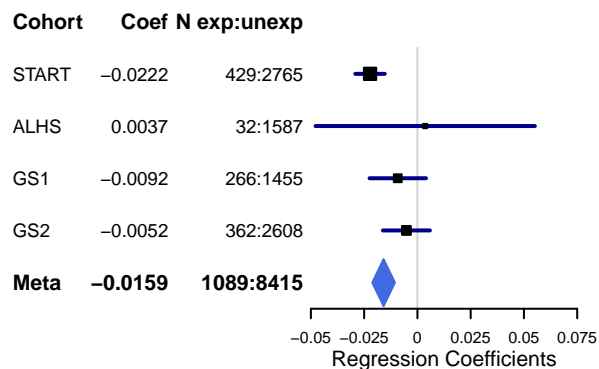

cg09932507

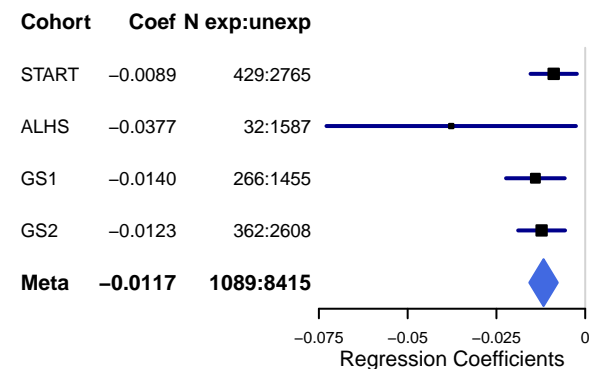

cg01212120

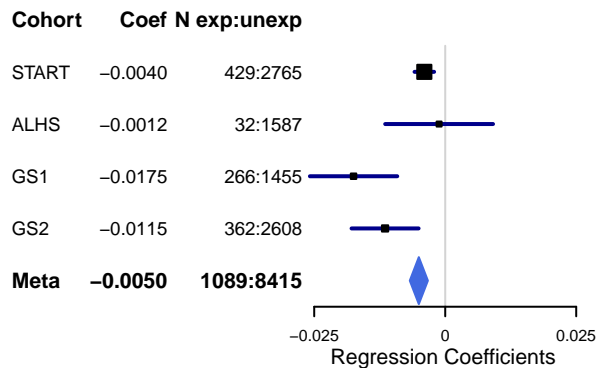

cg18842174

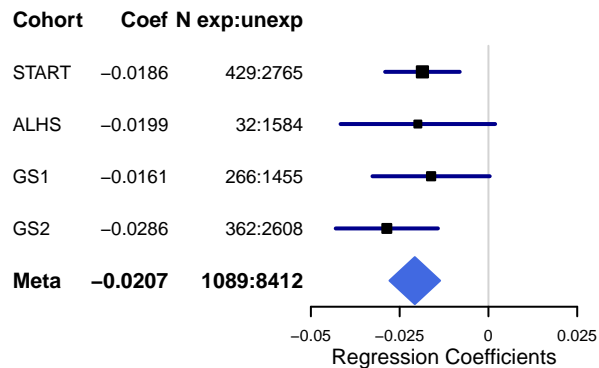

cg09653610

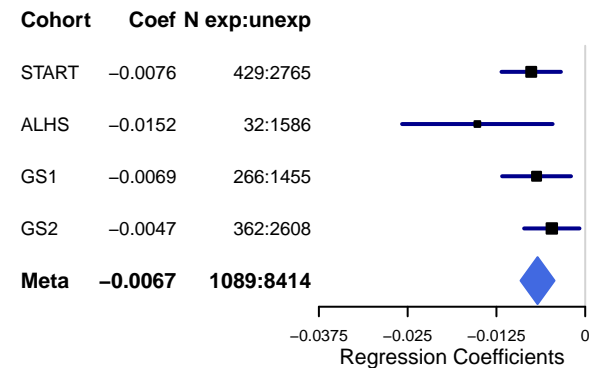

cg16032841

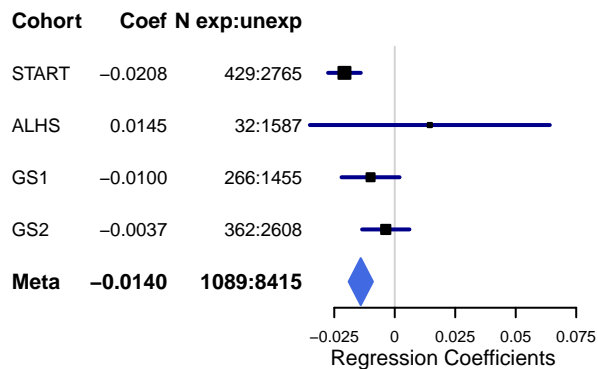

cg11603447

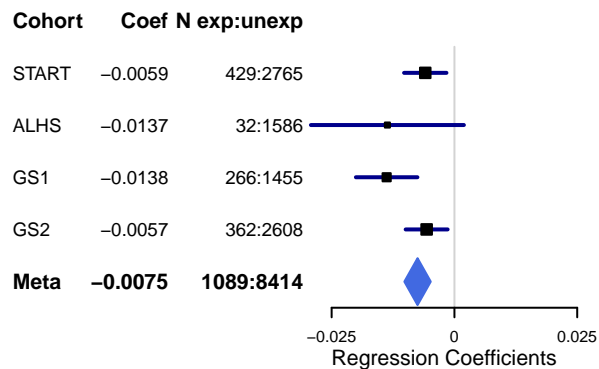

cg25057461

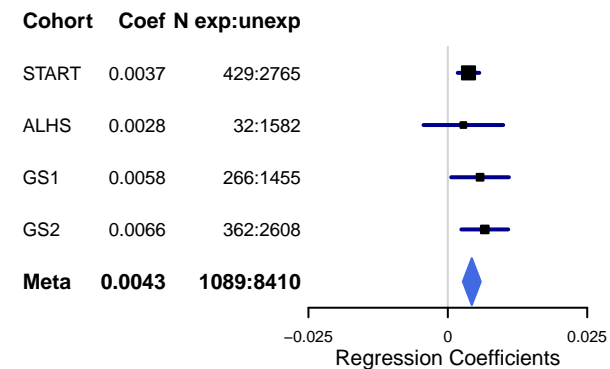

cg26582982

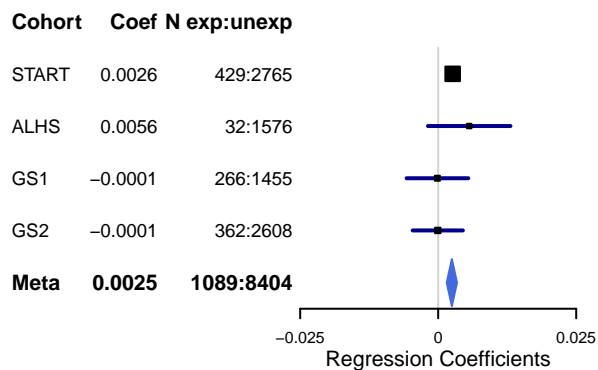

cg03482123

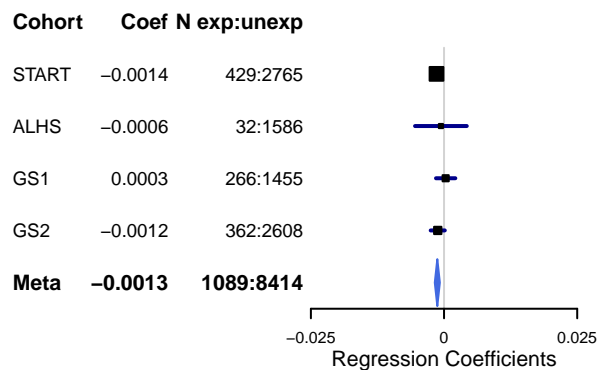

cg18560003

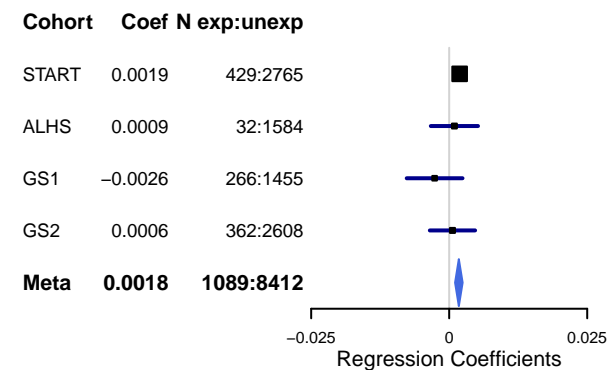

Supplement: Additional File S3 [file mmc3.pdf]

cg26697320

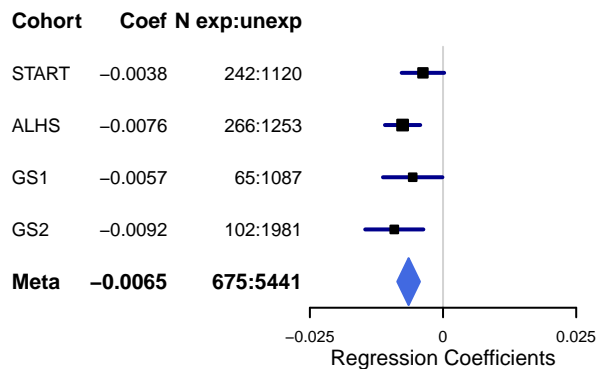

cg20562586

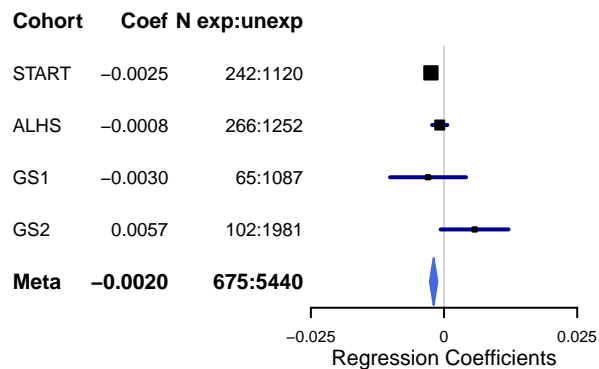

cg27647038

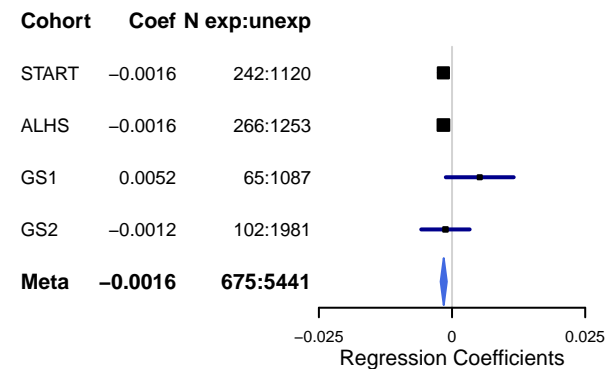

cg06987255

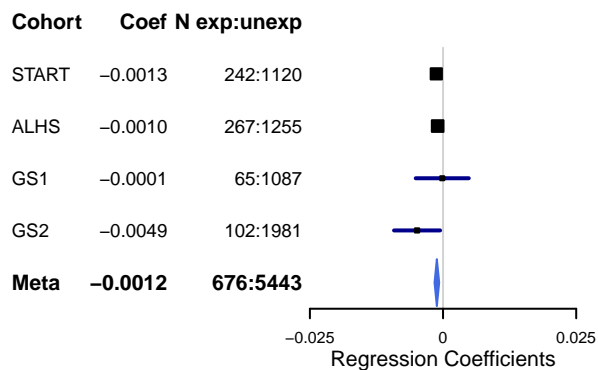

cg17669497

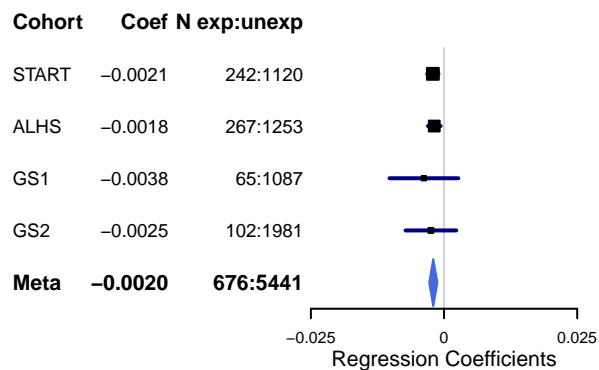

cg01678383

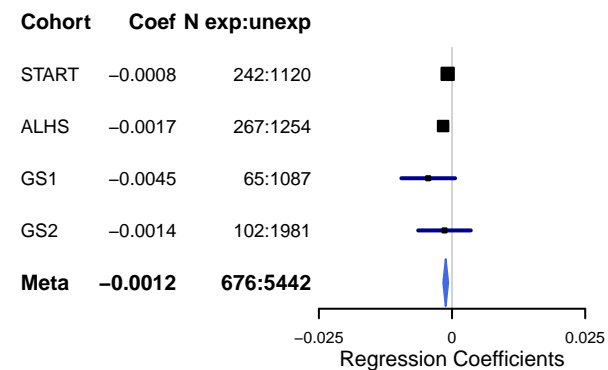

Supplement: Additional File S5 [file mmc5.pdf]

a)

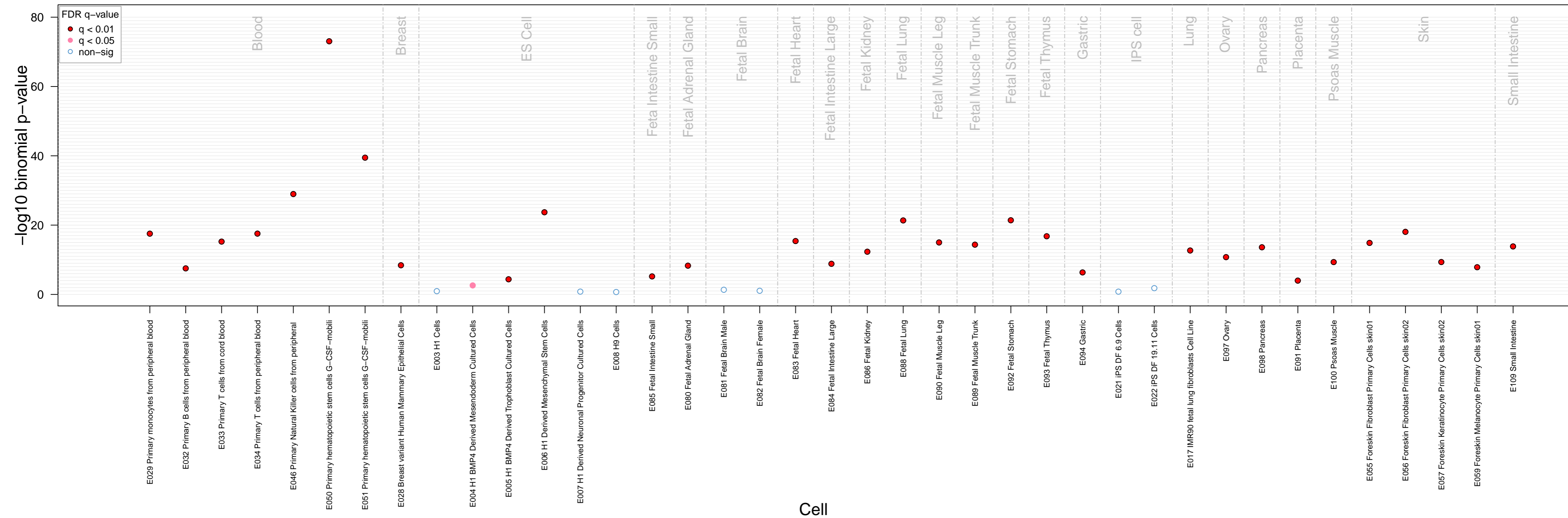

b)

$-\log_{10} \text{binomial } p\text{-value}$

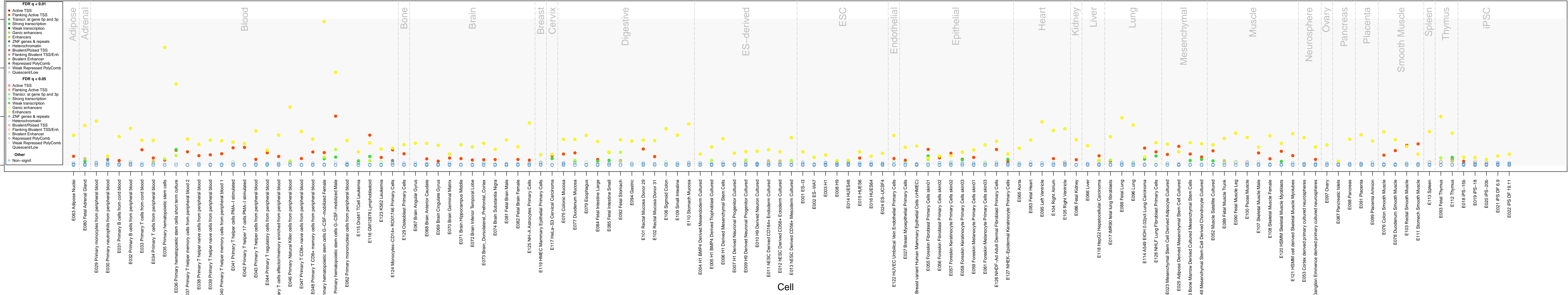

c)

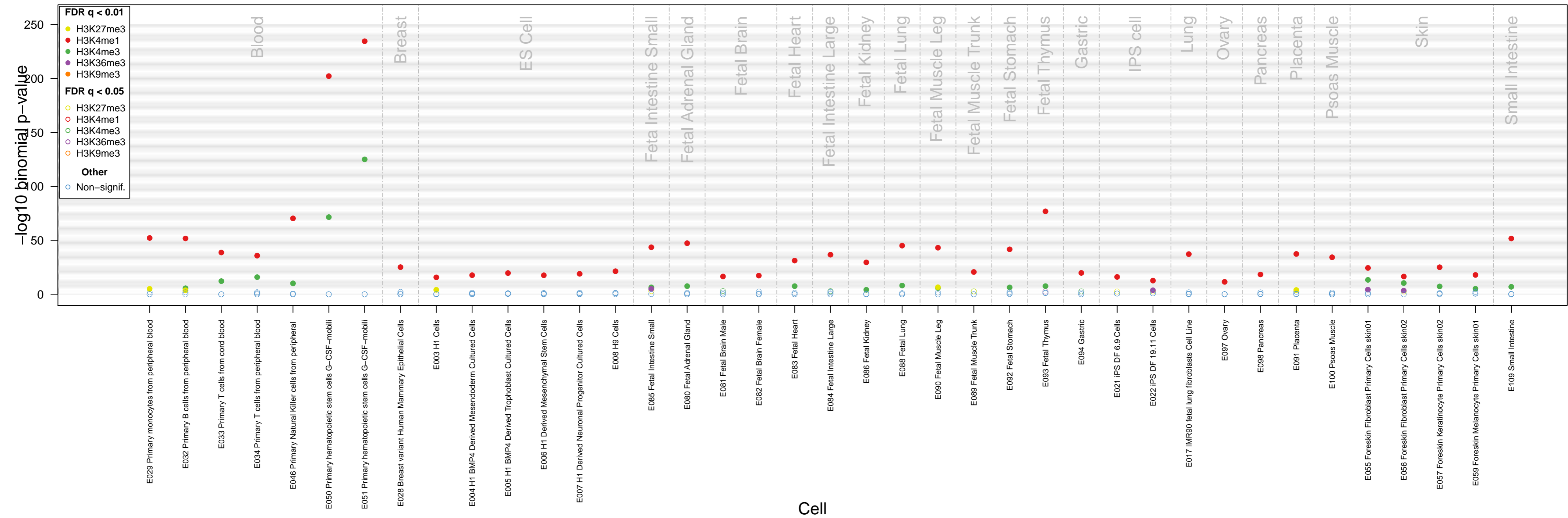

Supplement: Additional File S6 [file mmc6.pdf]

a)

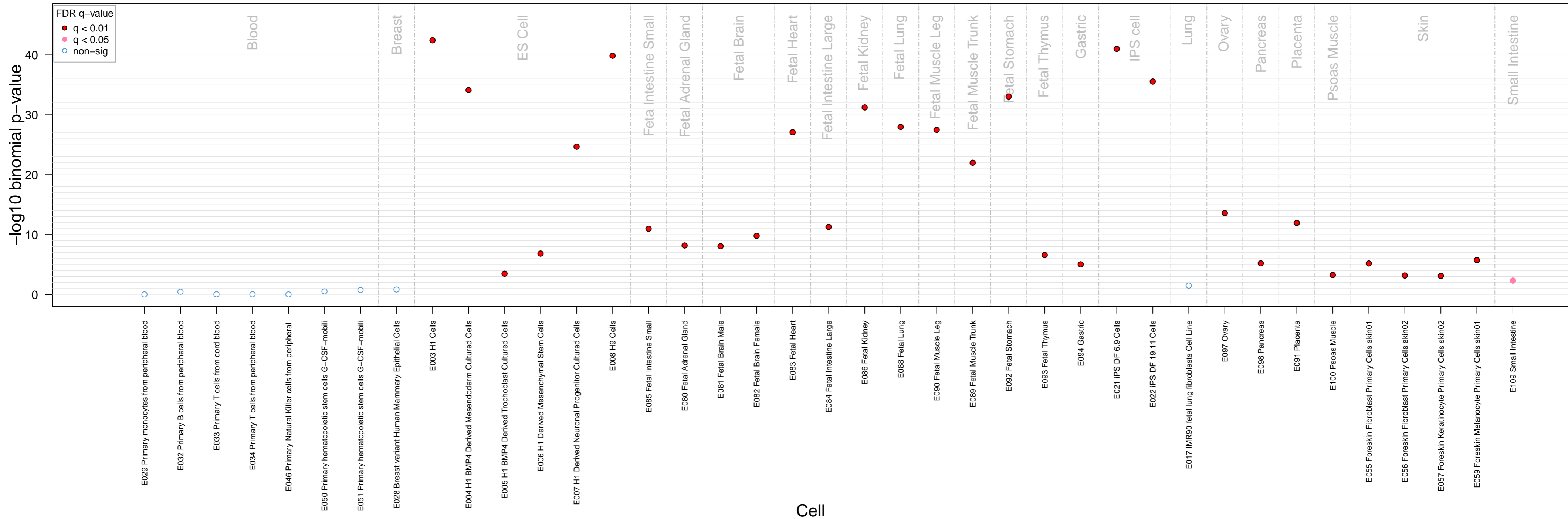

b)

$-\log_{10}$  binomial p-value

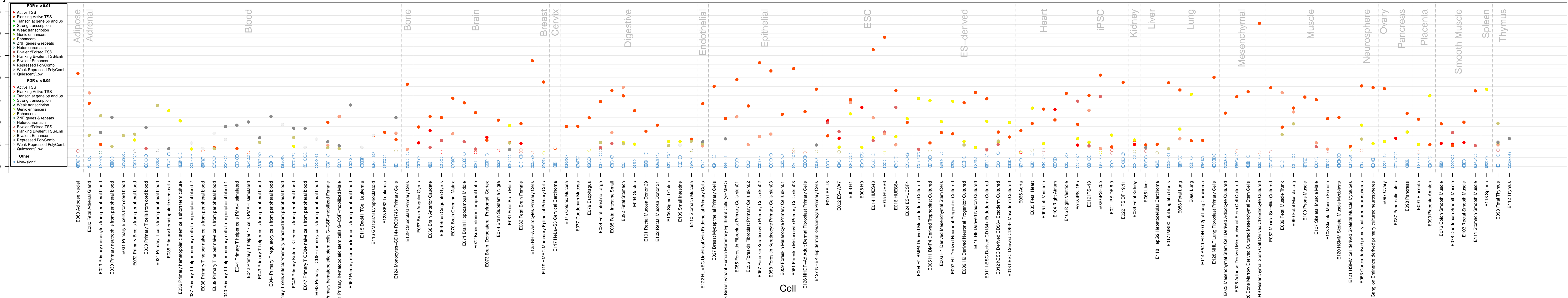

c)

$-\log_{10}$  binomial p-value

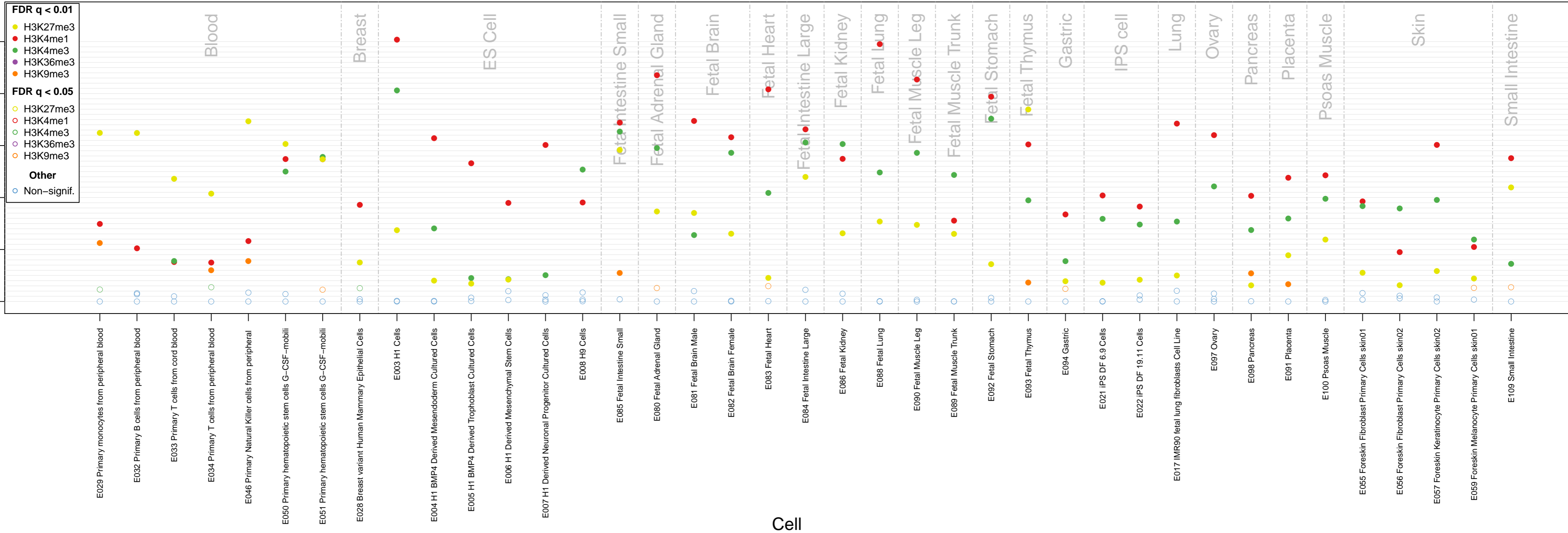

Supplement: Additional File S7 [file mmc7.pdf]
