# Supplementary material for: Comprehensive evaluation of smoking exposures and their interactions on DNA methylation
Source: eBioMedicine. 2024 Jan 9;100:104956. doi: 10.1016/j.ebiom.2023.104956 (PMC10825325; doi:10.1016/j.ebiom.2023.104956)
Supplement: Additional File S4 [file mmc4.pdf]

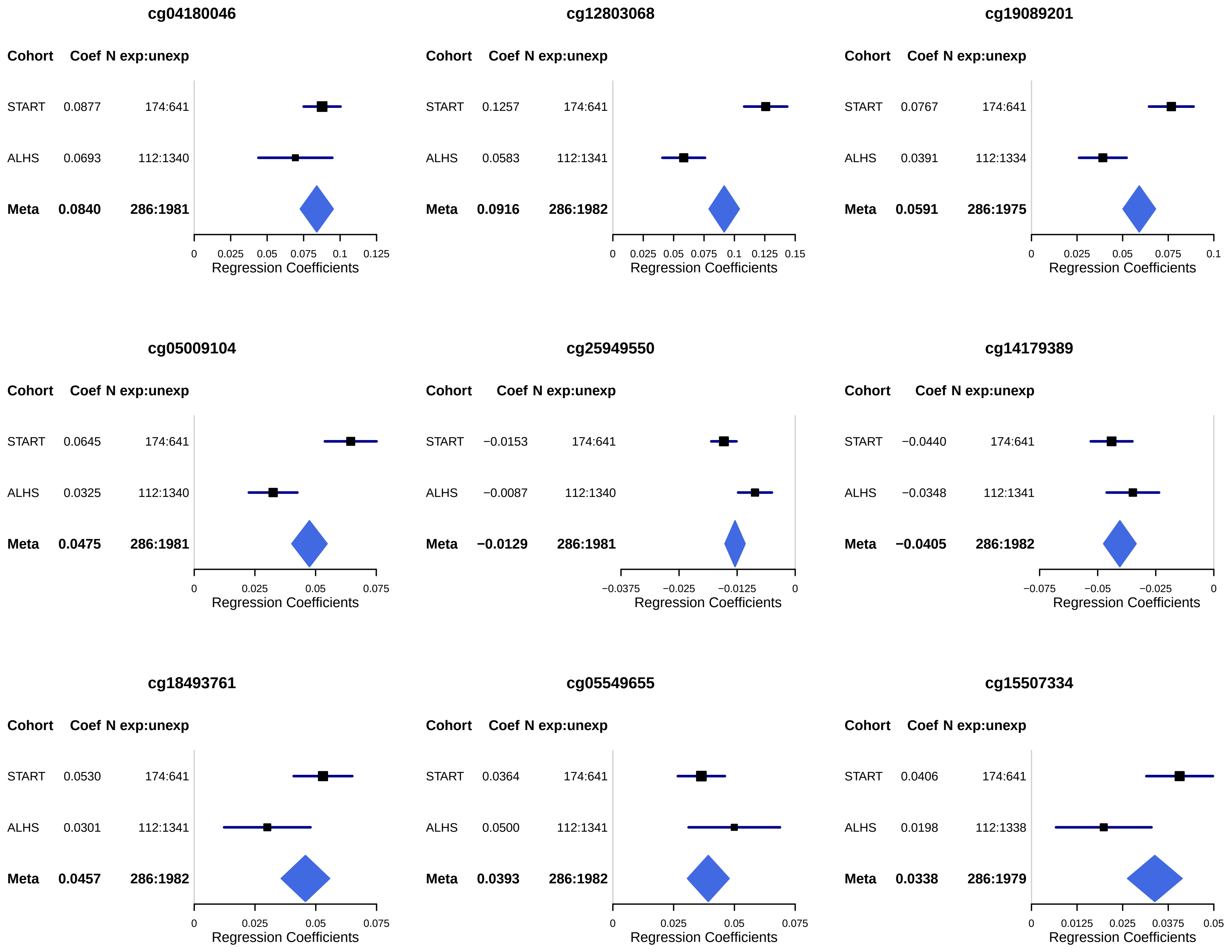
cg04180046
Cohort Coef N exp:unexp
START 0.0877 174:641
ALHS 0.0693 112:1340
Meta 0.0840 286:1981
0 0.025 0.05 0.075 0.1 0.125
Regression Coefficients
cg12803068
Cohort Coef N exp:unexp
START 0.1257 174:641
ALHS 0.0583 112:1341
Meta 0.0916 286:1982
0 0.025 0.05 0.075 0.1 0.125 0.15
Regression Coefficients
cg19089201
Cohort Coef N exp:unexp
START 0.0767 174:641
ALHS 0.0391 112:1334
Meta 0.0591 286:1975
0 0.025 0.05 0.075 0.1
Regression Coefficients
cg05009104
Cohort Coef N exp:unexp
START 0.0645 174:641
ALHS 0.0325 112:1340
Meta 0.0475 286:1981
0 0.025 0.05 0.075
Regression Coefficients
cg25949550
Cohort Coef N exp:unexp
START −0.0153 174:641
ALHS −0.0087 112:1340
Meta −0.0129 286:1981
−0.0375 −0.025 −0.0125 0
Regression Coefficients
cg14179389
Cohort Coef N exp:unexp
START −0.0440 174:641
ALHS −0.0348 112:1341
Meta −0.0405 286:1982
−0.075 −0.05 −0.025 0
Regression Coefficients
cg18493761
Cohort Coef N exp:unexp
START 0.0530 174:641
ALHS 0.0301 112:1341
Meta 0.0457 286:1982
0 0.025 0.05 0.075
Regression Coefficients
cg05549655
Cohort Coef N exp:unexp
START 0.0364 174:641
ALHS 0.0500 112:1341
Meta 0.0393 286:1982
0 0.025 0.05 0.075
Regression Coefficients
cg15507334
Cohort Coef N exp:unexp
START 0.0406 174:641
ALHS 0.0198 112:1338
Meta 0.0338 286:1979
0 0.0125 0.025 0.0375 0.05
Regression Coefficients

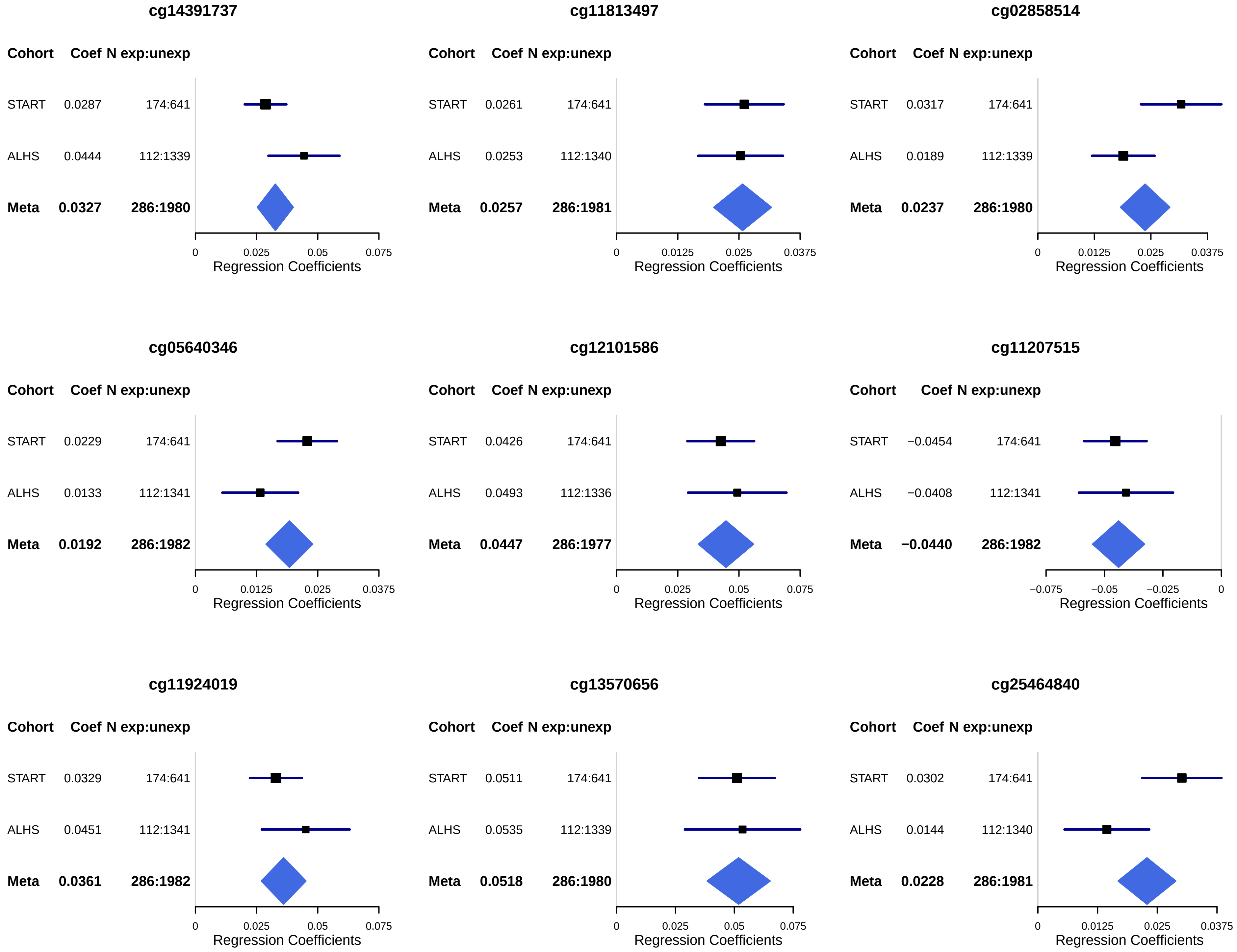
cg14391737
Cohort Coef N exp:unexp
START 0.0287 174:641
ALHS 0.0444 112:1339
Meta 0.0327 286:1980
0 0.025 0.05 0.075
Regression Coefficients
cg11813497
Cohort Coef N exp:unexp
START 0.0261 174:641
ALHS 0.0253 112:1340
Meta 0.0257 286:1981
0 0.0125 0.025 0.0375
Regression Coefficients
cg02858514
Cohort Coef N exp:unexp
START 0.0317 174:641
ALHS 0.0189 112:1339
Meta 0.0237 286:1980
0 0.0125 0.025 0.0375
Regression Coefficients
cg05640346
Cohort Coef N exp:unexp
START 0.0229 174:641
ALHS 0.0133 112:1341
Meta 0.0192 286:1982
0 0.0125 0.025 0.0375
Regression Coefficients
cg12101586
Cohort Coef N exp:unexp
START 0.0426 174:641
ALHS 0.0493 112:1336
Meta 0.0447 286:1977
0 0.025 0.05 0.075
Regression Coefficients
cg11207515
Cohort Coef N exp:unexp
START −0.0454 174:641
ALHS −0.0408 112:1341
Meta −0.0440 286:1982
−0.075 −0.05 −0.025 0
Regression Coefficients
cg11924019
Cohort Coef N exp:unexp
START 0.0329 174:641
ALHS 0.0451 112:1341
Meta 0.0361 286:1982
0 0.025 0.05 0.075
Regression Coefficients
cg13570656
Cohort Coef N exp:unexp
START 0.0511 174:641
ALHS 0.0535 112:1339
Meta 0.0518 286:1980
0 0.025 0.05 0.075
Regression Coefficients
cg25464840
Cohort Coef N exp:unexp
START 0.0302 174:641
ALHS 0.0144 112:1340
Meta 0.0228 286:1981
0 0.0125 0.025 0.0375
Regression Coefficients

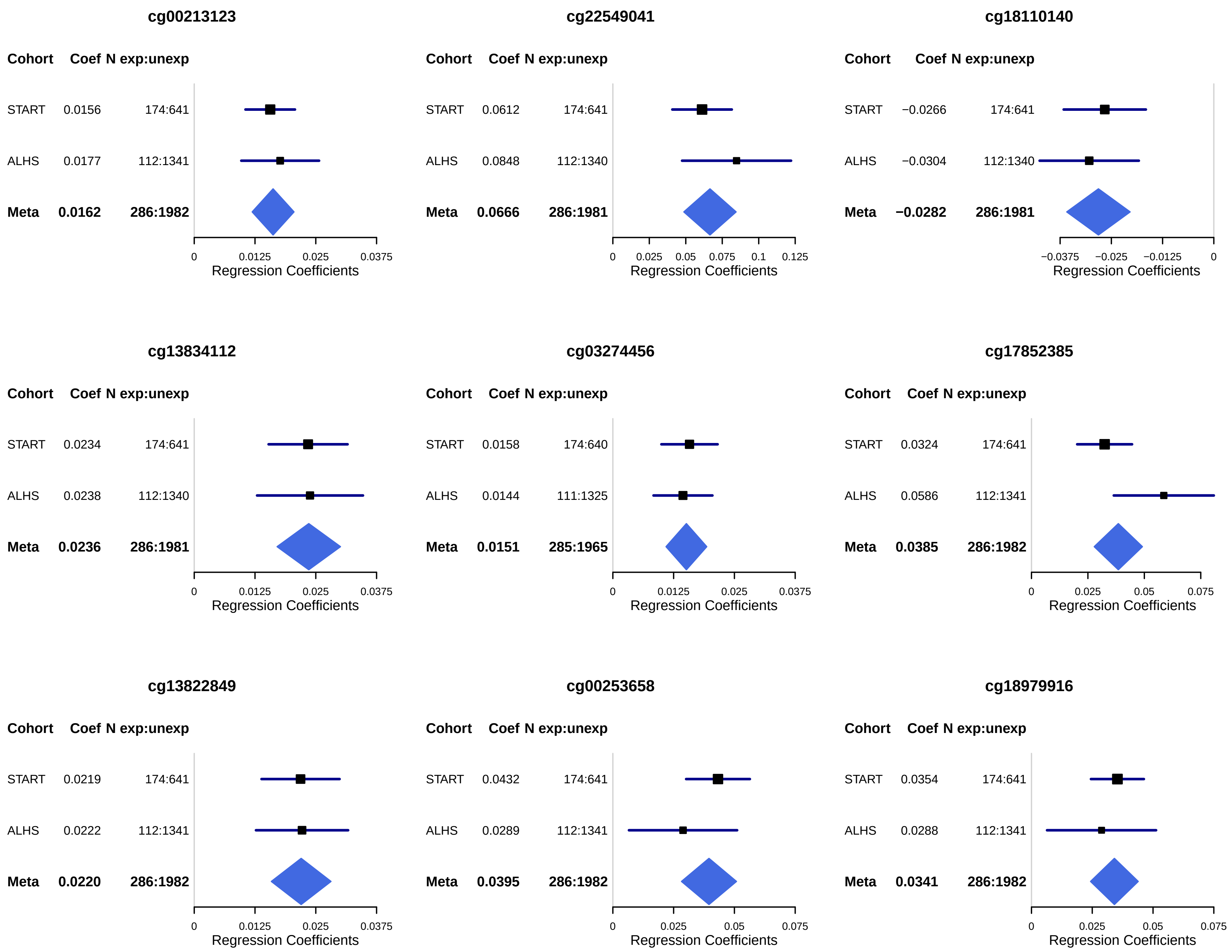
cg00213123
Cohort Coef N exp:unexp
START 0.0156 174:641
ALHS 0.0177 112:1341
Meta 0.0162 286:1982
0 0.0125 0.025 0.0375
Regression Coefficients
cg22549041
Cohort Coef N exp:unexp
START 0.0612 174:641
ALHS 0.0848 112:1340
Meta 0.0666 286:1981
0 0.025 0.05 0.075 0.1 0.125
Regression Coefficients
cg18110140
Cohort Coef N exp:unexp
START −0.0266 174:641
ALHS −0.0304 112:1340
Meta −0.0282 286:1981
−0.0375 −0.025 −0.0125 0
Regression Coefficients
cg13834112
Cohort Coef N exp:unexp
START 0.0234 174:641
ALHS 0.0238 112:1340
Meta 0.0236 286:1981
0 0.0125 0.025 0.0375
Regression Coefficients
cg03274456
Cohort Coef N exp:unexp
START 0.0158 174:640
ALHS 0.0144 111:1325
Meta 0.0151 285:1965
0 0.0125 0.025 0.0375
Regression Coefficients
cg17852385
Cohort Coef N exp:unexp
START 0.0324 174:641
ALHS 0.0586 112:1341
Meta 0.0385 286:1982
0 0.025 0.05 0.075
Regression Coefficients
cg13822849
Cohort Coef N exp:unexp
START 0.0219 174:641
ALHS 0.0222 112:1341
Meta 0.0220 286:1982
0 0.0125 0.025 0.0375
Regression Coefficients
cg00253658
Cohort Coef N exp:unexp
START 0.0432 174:641
ALHS 0.0289 112:1341
Meta 0.0395 286:1982
0 0.025 0.05 0.075
Regression Coefficients
cg18979916
Cohort Coef N exp:unexp
START 0.0354 174:641
ALHS 0.0288 112:1341
Meta 0.0341 286:1982
0 0.025 0.05 0.075
Regression Coefficients

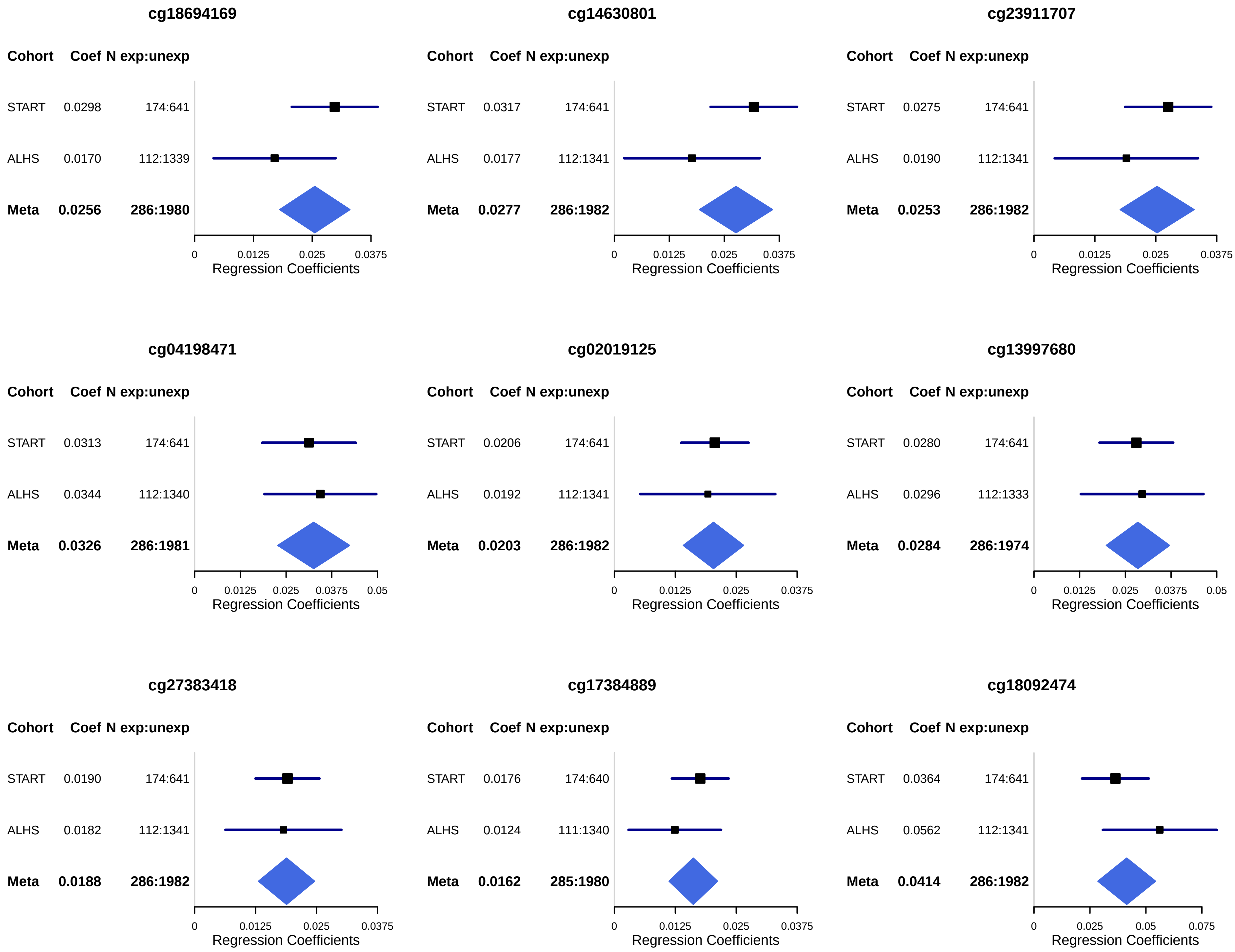
cg18694169
Cohort Coef N exp:unexp
START 0.0298 174:641
ALHS 0.0170 112:1339
Meta 0.0256 286:1980
0 0.0125 0.025 0.0375
Regression Coefficients
cg14630801
Cohort Coef N exp:unexp
START 0.0317 174:641
ALHS 0.0177 112:1341
Meta 0.0277 286:1982
0 0.0125 0.025 0.0375
Regression Coefficients
cg23911707
Cohort Coef N exp:unexp
START 0.0275 174:641
ALHS 0.0190 112:1341
Meta 0.0253 286:1982
0 0.0125 0.025 0.0375
Regression Coefficients
cg04198471
Cohort Coef N exp:unexp
START 0.0313 174:641
ALHS 0.0344 112:1340
Meta 0.0326 286:1981
0 0.0125 0.025 0.0375 0.05
Regression Coefficients
cg02019125
Cohort Coef N exp:unexp
START 0.0206 174:641
ALHS 0.0192 112:1341
Meta 0.0203 286:1982
0 0.0125 0.025 0.0375
Regression Coefficients
cg13997680
Cohort Coef N exp:unexp
START 0.0280 174:641
ALHS 0.0296 112:1333
Meta 0.0284 286:1974
0 0.0125 0.025 0.0375 0.05
Regression Coefficients
cg27383418
Cohort Coef N exp:unexp
START 0.0190 174:641
ALHS 0.0182 112:1341
Meta 0.0188 286:1982
0 0.0125 0.025 0.0375
Regression Coefficients
cg17384889
Cohort Coef N exp:unexp
START 0.0176 174:640
ALHS 0.0124 111:1340
Meta 0.0162 285:1980
0 0.0125 0.025 0.0375
Regression Coefficients
cg18092474
Cohort Coef N exp:unexp
START 0.0364 174:641
ALHS 0.0562 112:1341
Meta 0.0414 286:1982
0 0.025 0.05 0.075
Regression Coefficients

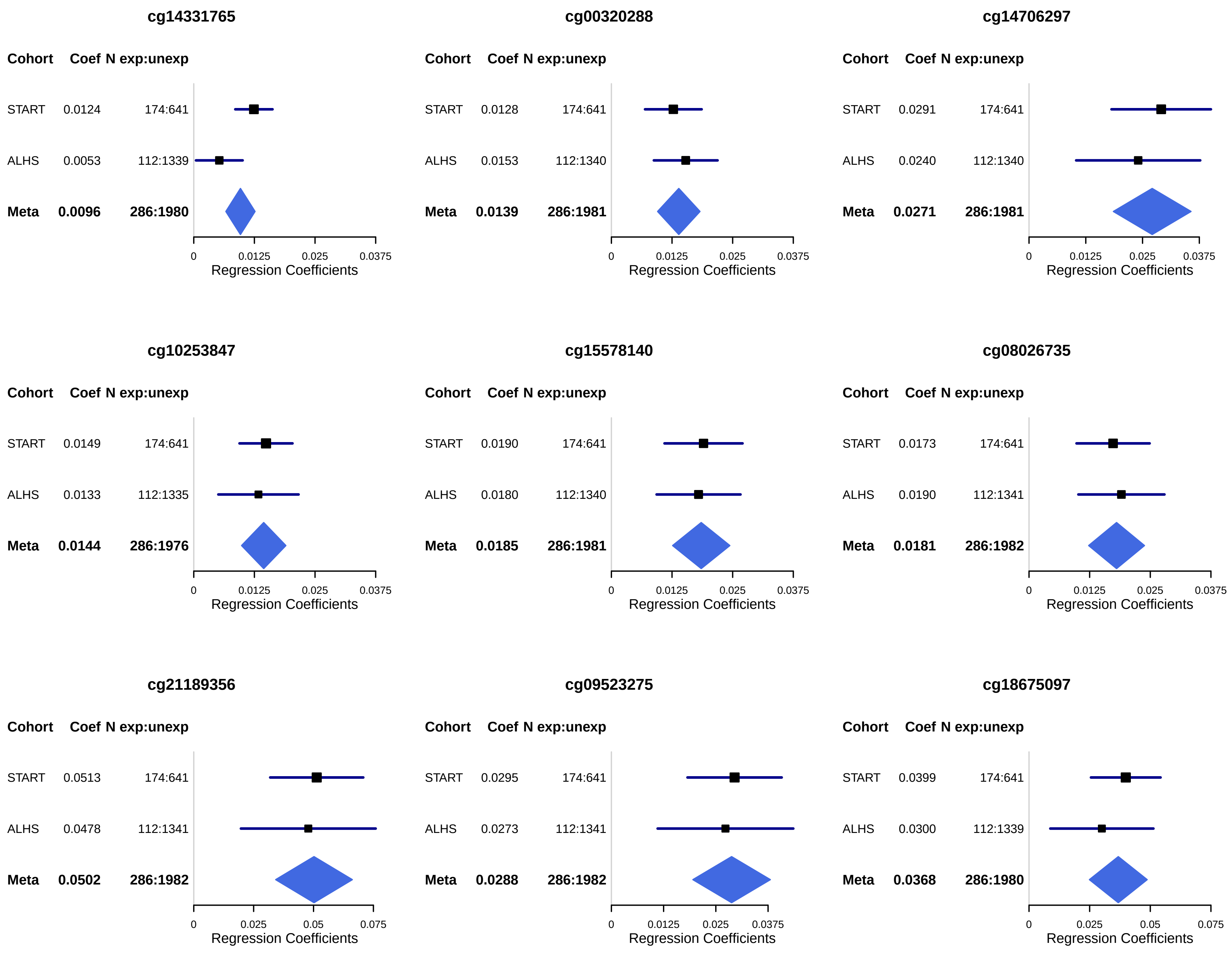
cg14331765
Cohort Coef N exp:unexp
START 0.0124 174:641
ALHS 0.0053 112:1339
Meta 0.0096 286:1980
0 0.0125 0.025 0.0375
Regression Coefficients
cg00320288
Cohort Coef N exp:unexp
START 0.0128 174:641
ALHS 0.0153 112:1340
Meta 0.0139 286:1981
0 0.0125 0.025 0.0375
Regression Coefficients
cg14706297
Cohort Coef N exp:unexp
START 0.0291 174:641
ALHS 0.0240 112:1340
Meta 0.0271 286:1981
0 0.0125 0.025 0.0375
Regression Coefficients
cg10253847
Cohort Coef N exp:unexp
START 0.0149 174:641
ALHS 0.0133 112:1335
Meta 0.0144 286:1976
0 0.0125 0.025 0.0375
Regression Coefficients
cg15578140
Cohort Coef N exp:unexp
START 0.0190 174:641
ALHS 0.0180 112:1340
Meta 0.0185 286:1981
0 0.0125 0.025 0.0375
Regression Coefficients
cg08026735
Cohort Coef N exp:unexp
START 0.0173 174:641
ALHS 0.0190 112:1341
Meta 0.0181 286:1982
0 0.0125 0.025 0.0375
Regression Coefficients
cg21189356
Cohort Coef N exp:unexp
START 0.0513 174:641
ALHS 0.0478 112:1341
Meta 0.0502 286:1982
0 0.025 0.05 0.075
Regression Coefficients
cg09523275
Cohort Coef N exp:unexp
START 0.0295 174:641
ALHS 0.0273 112:1341
Meta 0.0288 286:1982
0 0.0125 0.025 0.0375
Regression Coefficients
cg18675097
Cohort Coef N exp:unexp
START 0.0399 174:641
ALHS 0.0300 112:1339
Meta 0.0368 286:1980
0 0.025 0.05 0.075
Regression Coefficients

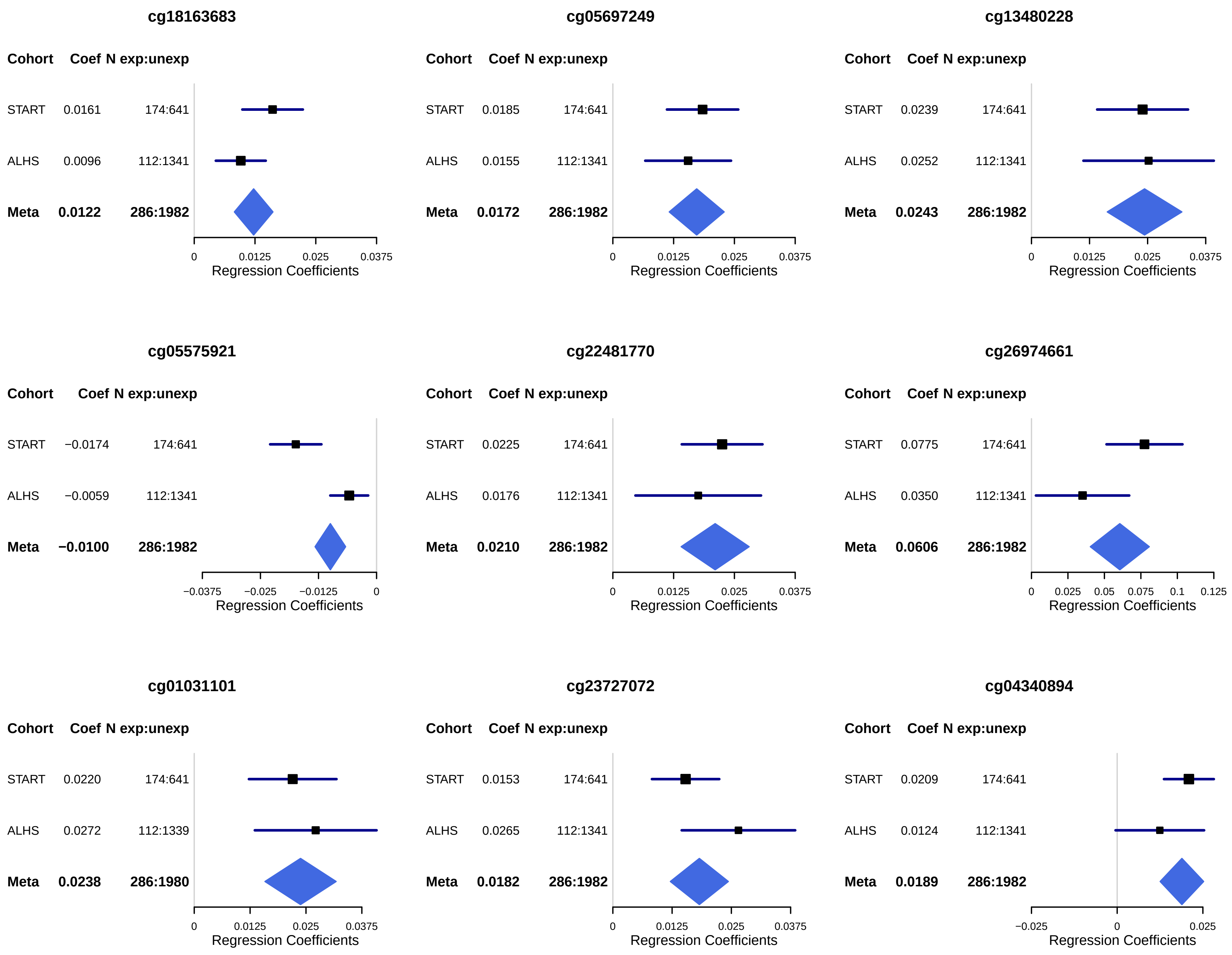
cg18163683
Cohort Coef N exp:unexp
START 0.0161 174:641
ALHS 0.0096 112:1341
Meta 0.0122 286:1982
0 0.0125 0.025 0.0375
Regression Coefficients
cg05697249
Cohort Coef N exp:unexp
START 0.0185 174:641
ALHS 0.0155 112:1341
Meta 0.0172 286:1982
0 0.0125 0.025 0.0375
Regression Coefficients
cg13480228
Cohort Coef N exp:unexp
START 0.0239 174:641
ALHS 0.0252 112:1341
Meta 0.0243 286:1982
0 0.0125 0.025 0.0375
Regression Coefficients
cg05575921
Cohort Coef N exp:unexp
START −0.0174 174:641
ALHS −0.0059 112:1341
Meta −0.0100 286:1982
−0.0375 −0.025 −0.0125 0
Regression Coefficients
cg22481770
Cohort Coef N exp:unexp
START 0.0225 174:641
ALHS 0.0176 112:1341
Meta 0.0210 286:1982
0 0.0125 0.025 0.0375
Regression Coefficients
cg26974661
Cohort Coef N exp:unexp
START 0.0775 174:641
ALHS 0.0350 112:1341
Meta 0.0606 286:1982
0 0.025 0.05 0.075 0.1 0.125
Regression Coefficients
cg01031101
Cohort Coef N exp:unexp
START 0.0220 174:641
ALHS 0.0272 112:1339
Meta 0.0238 286:1980
0 0.0125 0.025 0.0375
Regression Coefficients
cg23727072
Cohort Coef N exp:unexp
START 0.0153 174:641
ALHS 0.0265 112:1341
Meta 0.0182 286:1982
0 0.0125 0.025 0.0375
Regression Coefficients
cg04340894
Cohort Coef N exp:unexp
START 0.0209 174:641
ALHS 0.0124 112:1341
Meta 0.0189 286:1982
−0.025 0 0.025
Regression Coefficients

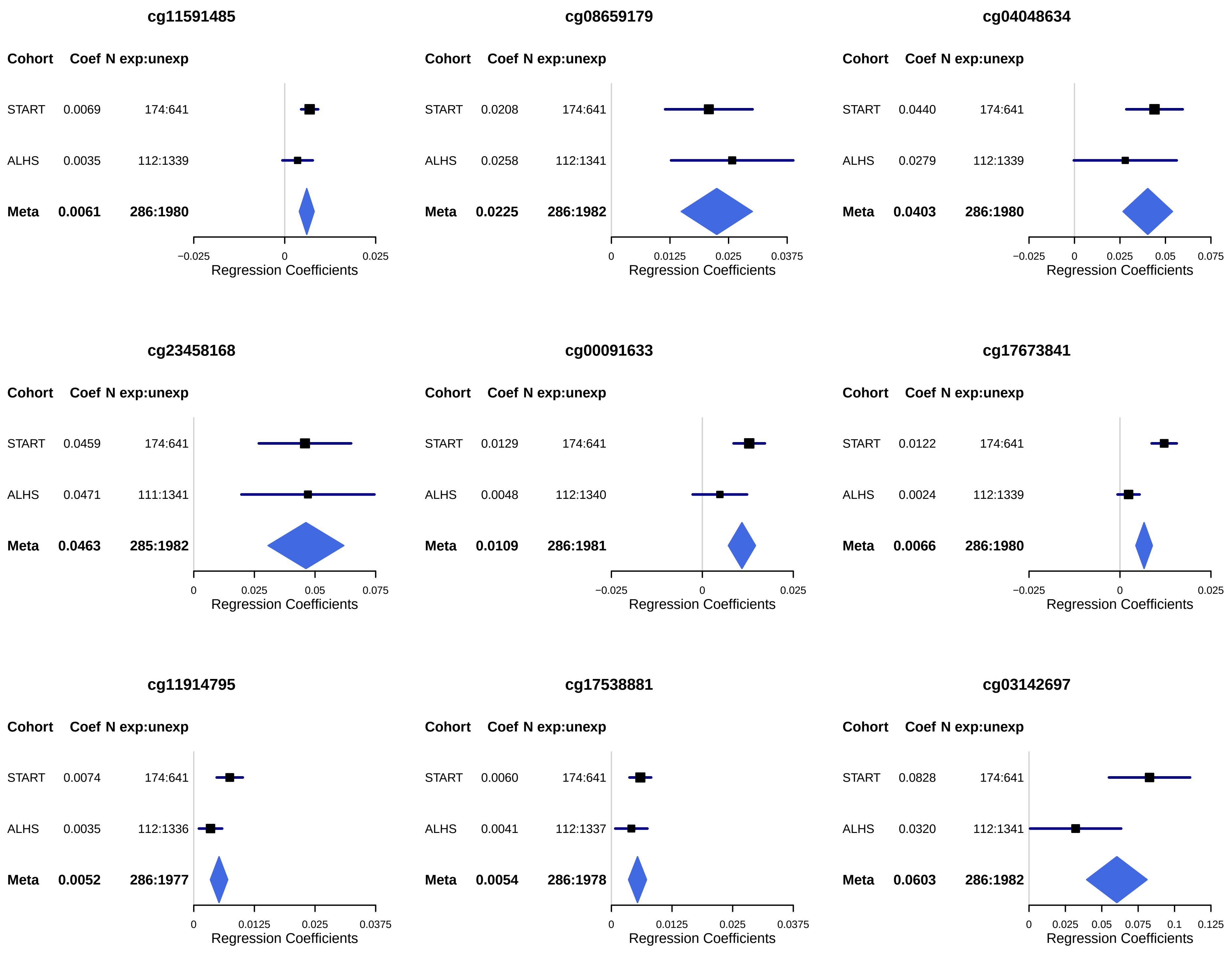
cg11591485
Cohort Coef N exp:unexp
START 0.0069 174:641
ALHS 0.0035 112:1339
Meta 0.0061 286:1980
−0.025 0 0.025
Regression Coefficients
cg08659179
Cohort Coef N exp:unexp
START 0.0208 174:641
ALHS 0.0258 112:1341
Meta 0.0225 286:1982
0 0.0125 0.025 0.0375
Regression Coefficients
cg04048634
Cohort Coef N exp:unexp
START 0.0440 174:641
ALHS 0.0279 112:1339
Meta 0.0403 286:1980
−0.025 0 0.025 0.05 0.075
Regression Coefficients
cg23458168
Cohort Coef N exp:unexp
START 0.0459 174:641
ALHS 0.0471 111:1341
Meta 0.0463 285:1982
0 0.025 0.05 0.075
Regression Coefficients
cg00091633
Cohort Coef N exp:unexp
START 0.0129 174:641
ALHS 0.0048 112:1340
Meta 0.0109 286:1981
−0.025 0 0.025
Regression Coefficients
cg17673841
Cohort Coef N exp:unexp
START 0.0122 174:641
ALHS 0.0024 112:1339
Meta 0.0066 286:1980
−0.025 0 0.025
Regression Coefficients
cg11914795
Cohort Coef N exp:unexp
START 0.0074 174:641
ALHS 0.0035 112:1336
Meta 0.0052 286:1977
0 0.0125 0.025 0.0375
Regression Coefficients
cg17538881
Cohort Coef N exp:unexp
START 0.0060 174:641
ALHS 0.0041 112:1337
Meta 0.0054 286:1978
0 0.0125 0.025 0.0375
Regression Coefficients
cg03142697
Cohort Coef N exp:unexp
START 0.0828 174:641
ALHS 0.0320 112:1341
Meta 0.0603 286:1982
0 0.025 0.05 0.075 0.1 0.125
Regression Coefficients

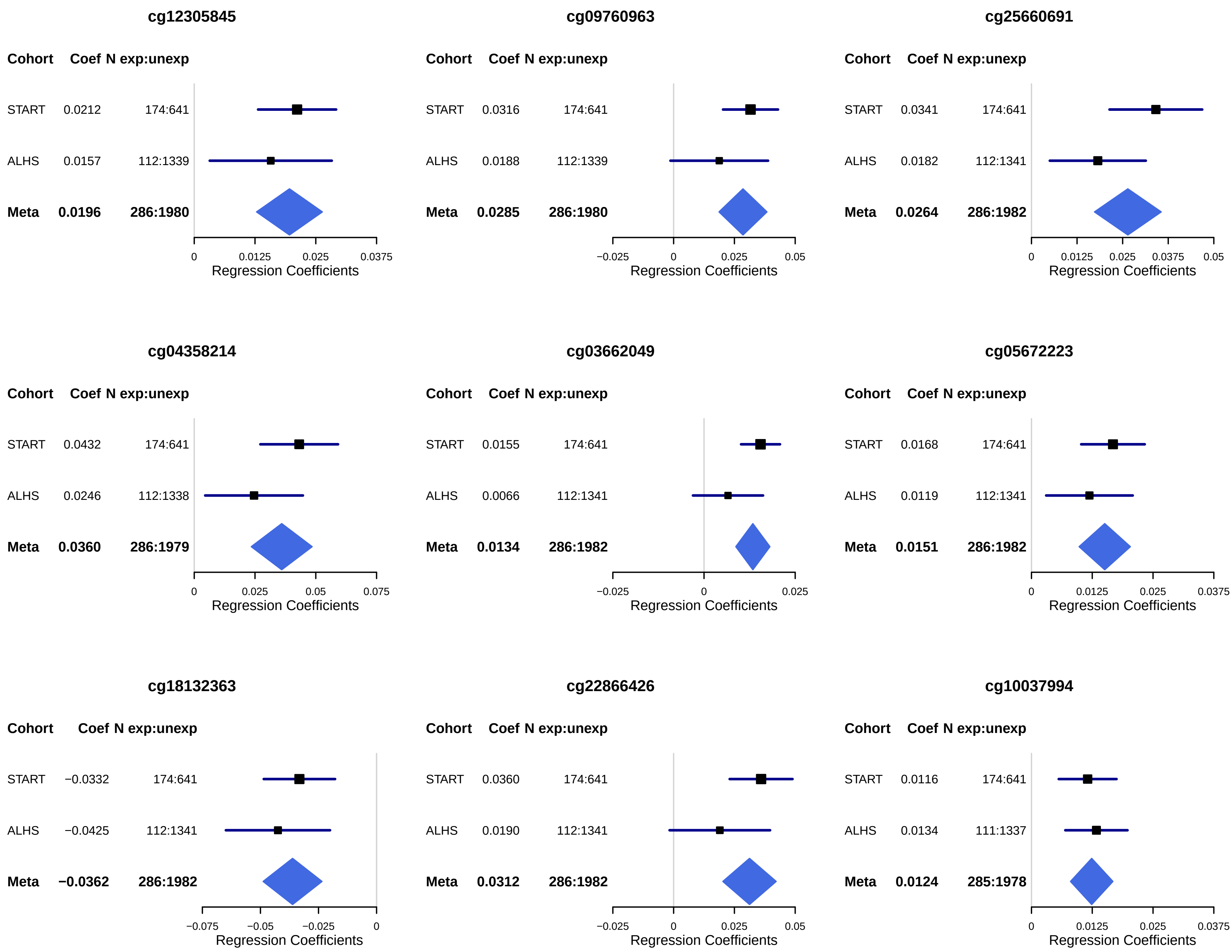
cg12305845
Cohort Coef N exp:unexp
START 0.0212 174:641
ALHS 0.0157 112:1339
Meta 0.0196 286:1980
0 0.0125 0.025 0.0375
Regression Coefficients
cg09760963
Cohort Coef N exp:unexp
START 0.0316 174:641
ALHS 0.0188 112:1339
Meta 0.0285 286:1980
−0.025 0 0.025 0.05
Regression Coefficients
cg25660691
Cohort Coef N exp:unexp
START 0.0341 174:641
ALHS 0.0182 112:1341
Meta 0.0264 286:1982
0 0.0125 0.025 0.0375 0.05
Regression Coefficients
cg04358214
Cohort Coef N exp:unexp
START 0.0432 174:641
ALHS 0.0246 112:1338
Meta 0.0360 286:1979
0 0.025 0.05 0.075
Regression Coefficients
cg03662049
Cohort Coef N exp:unexp
START 0.0155 174:641
ALHS 0.0066 112:1341
Meta 0.0134 286:1982
−0.025 0 0.025
Regression Coefficients
cg05672223
Cohort Coef N exp:unexp
START 0.0168 174:641
ALHS 0.0119 112:1341
Meta 0.0151 286:1982
0 0.0125 0.025 0.0375
Regression Coefficients
cg18132363
Cohort Coef N exp:unexp
START −0.0332 174:641
ALHS −0.0425 112:1341
Meta −0.0362 286:1982
−0.075 −0.05 −0.025 0
Regression Coefficients
cg22866426
Cohort Coef N exp:unexp
START 0.0360 174:641
ALHS 0.0190 112:1341
Meta 0.0312 286:1982
−0.025 0 0.025 0.05
Regression Coefficients
cg10037994
Cohort Coef N exp:unexp
START 0.0116 174:641
ALHS 0.0134 111:1337
Meta 0.0124 285:1978
0 0.0125 0.025 0.0375
Regression Coefficients

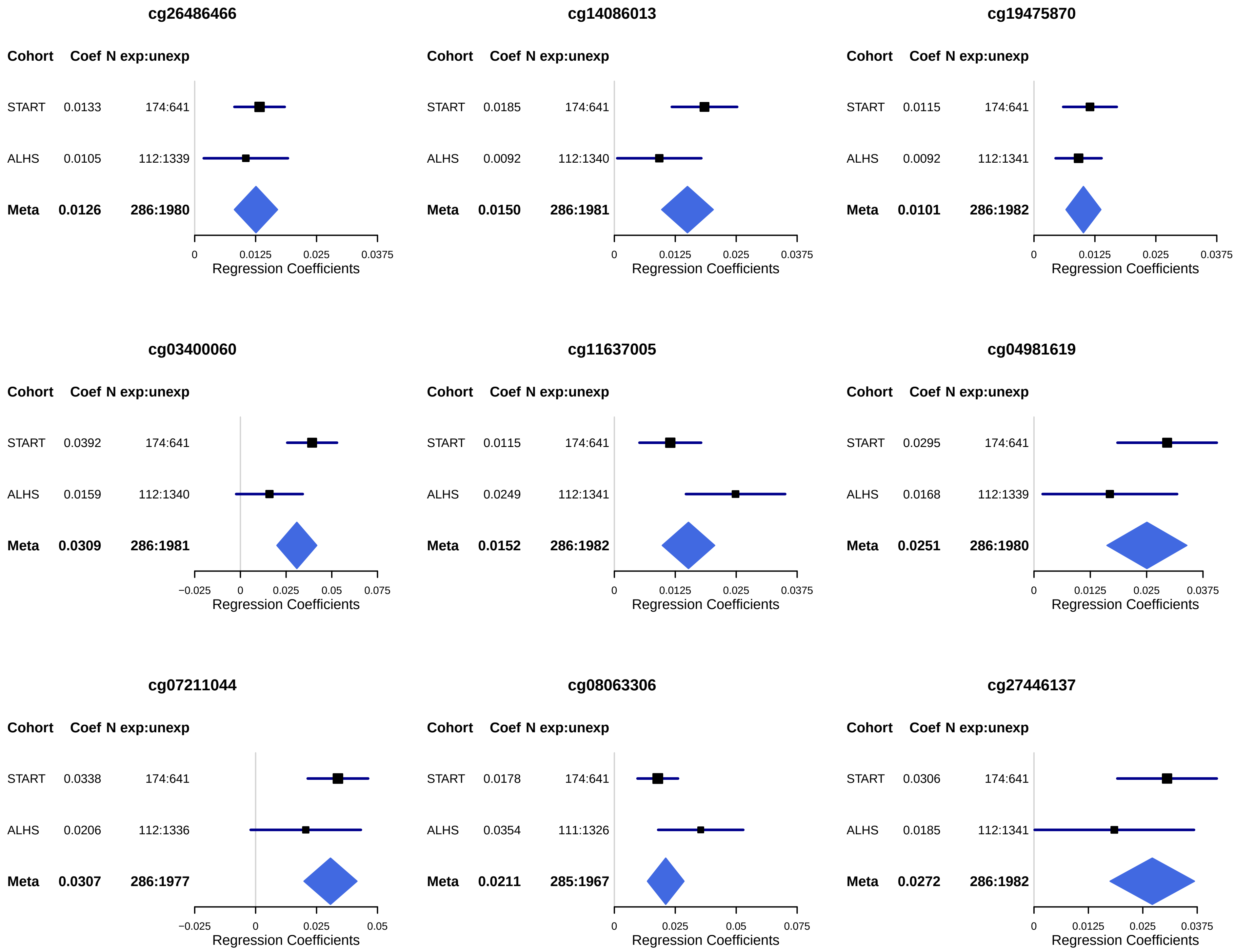
cg26486466
Cohort Coef N exp:unexp
START 0.0133 174:641
ALHS 0.0105 112:1339
Meta 0.0126 286:1980
0 0.0125 0.025 0.0375
Regression Coefficients
cg14086013
Cohort Coef N exp:unexp
START 0.0185 174:641
ALHS 0.0092 112:1340
Meta 0.0150 286:1981
0 0.0125 0.025 0.0375
Regression Coefficients
cg19475870
Cohort Coef N exp:unexp
START 0.0115 174:641
ALHS 0.0092 112:1341
Meta 0.0101 286:1982
0 0.0125 0.025 0.0375
Regression Coefficients
cg03400060
Cohort Coef N exp:unexp
START 0.0392 174:641
ALHS 0.0159 112:1340
Meta 0.0309 286:1981
−0.025 0 0.025 0.05 0.075
Regression Coefficients
cg11637005
Cohort Coef N exp:unexp
START 0.0115 174:641
ALHS 0.0249 112:1341
Meta 0.0152 286:1982
0 0.0125 0.025 0.0375
Regression Coefficients
cg04981619
Cohort Coef N exp:unexp
START 0.0295 174:641
ALHS 0.0168 112:1339
Meta 0.0251 286:1980
0 0.0125 0.025 0.0375
Regression Coefficients
cg07211044
Cohort Coef N exp:unexp
START 0.0338 174:641
ALHS 0.0206 112:1336
Meta 0.0307 286:1977
−0.025 0 0.025 0.05
Regression Coefficients
cg08063306
Cohort Coef N exp:unexp
START 0.0178 174:641
ALHS 0.0354 111:1326
Meta 0.0211 285:1967
0 0.025 0.05 0.075
Regression Coefficients
cg27446137
Cohort Coef N exp:unexp
START 0.0306 174:641
ALHS 0.0185 112:1341
Meta 0.0272 286:1982
0 0.0125 0.025 0.0375
Regression Coefficients

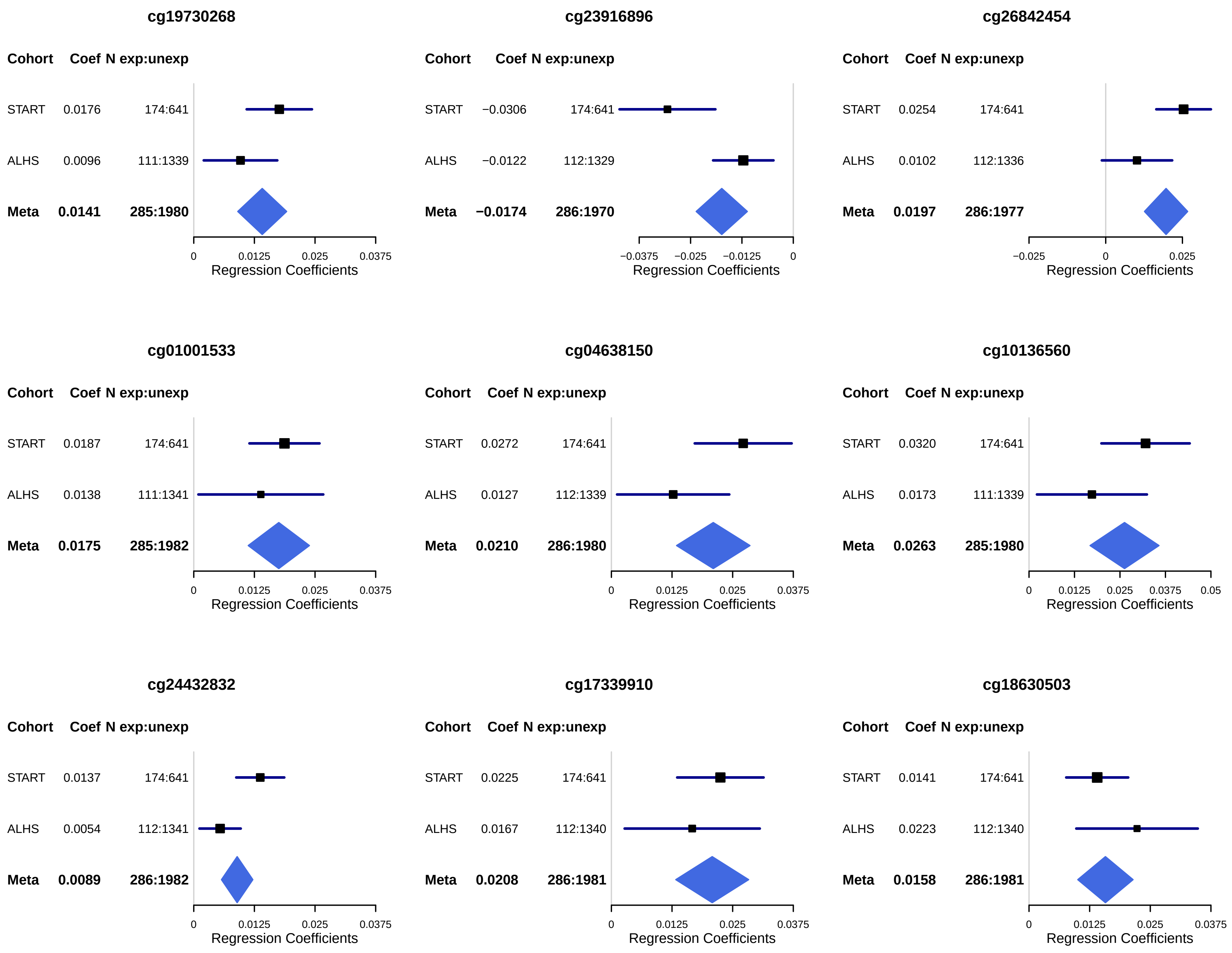
cg19730268
Cohort Coef N exp:unexp
START 0.0176 174:641
ALHS 0.0096 111:1339
Meta 0.0141 285:1980
0 0.0125 0.025 0.0375
Regression Coefficients
cg23916896
Cohort Coef N exp:unexp
START −0.0306 174:641
ALHS −0.0122 112:1329
Meta −0.0174 286:1970
−0.0375 −0.025 −0.0125 0
Regression Coefficients
cg26842454
Cohort Coef N exp:unexp
START 0.0254 174:641
ALHS 0.0102 112:1336
Meta 0.0197 286:1977
−0.025 0 0.025
Regression Coefficients
cg01001533
Cohort Coef N exp:unexp
START 0.0187 174:641
ALHS 0.0138 111:1341
Meta 0.0175 285:1982
0 0.0125 0.025 0.0375
Regression Coefficients
cg04638150
Cohort Coef N exp:unexp
START 0.0272 174:641
ALHS 0.0127 112:1339
Meta 0.0210 286:1980
0 0.0125 0.025 0.0375
Regression Coefficients
cg10136560
Cohort Coef N exp:unexp
START 0.0320 174:641
ALHS 0.0173 111:1339
Meta 0.0263 285:1980
0 0.0125 0.025 0.0375 0.05
Regression Coefficients
cg24432832
Cohort Coef N exp:unexp
START 0.0137 174:641
ALHS 0.0054 112:1341
Meta 0.0089 286:1982
0 0.0125 0.025 0.0375
Regression Coefficients
cg17339910
Cohort Coef N exp:unexp
START 0.0225 174:641
ALHS 0.0167 112:1340
Meta 0.0208 286:1981
0 0.0125 0.025 0.0375
Regression Coefficients
cg18630503
Cohort Coef N exp:unexp
START 0.0141 174:641
ALHS 0.0223 112:1340
Meta 0.0158 286:1981
0 0.0125 0.025 0.0375
Regression Coefficients

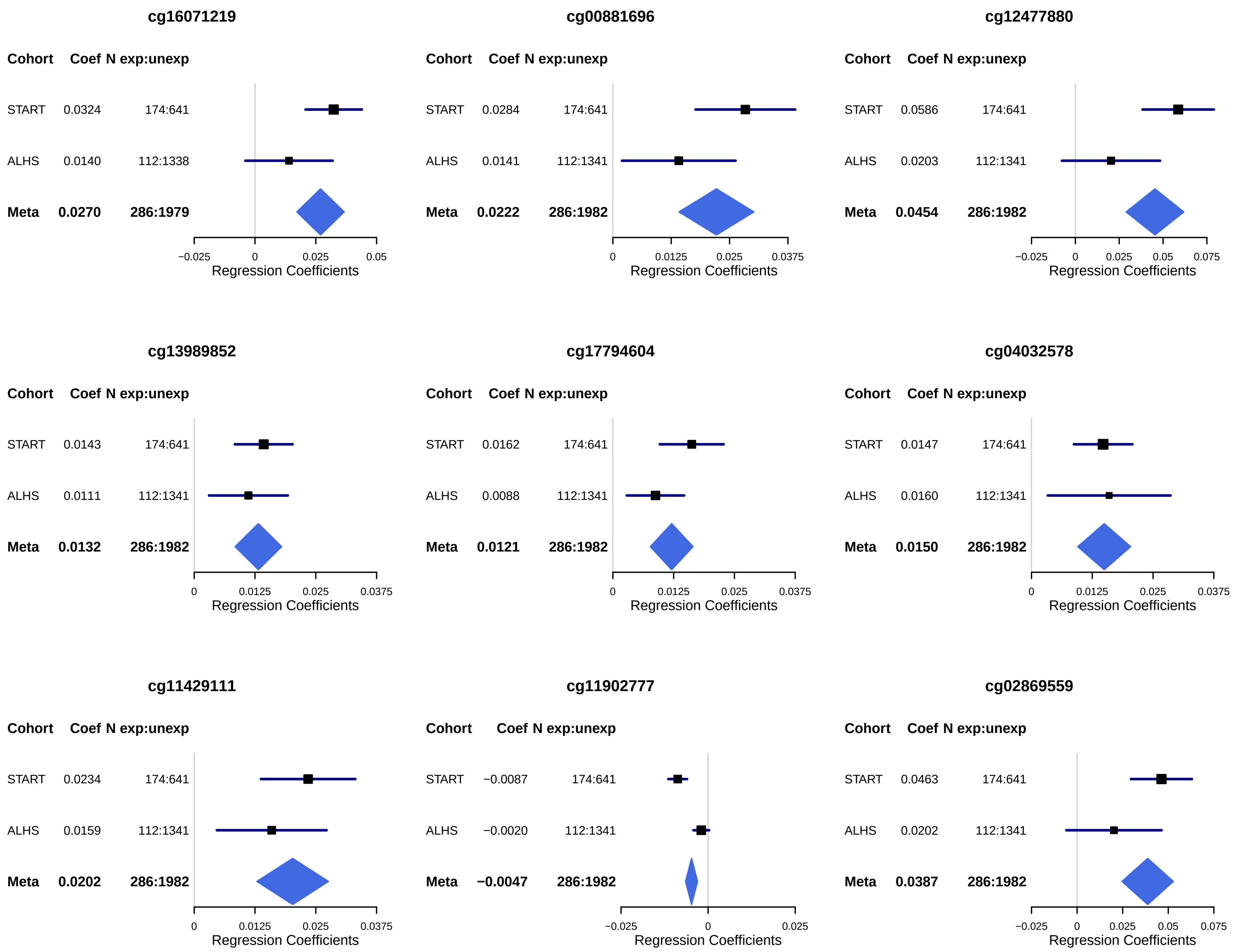
cg16071219
Cohort Coef N exp:unexp
START 0.0324 174:641
ALHS 0.0140 112:1338
Meta 0.0270 286:1979
−0.025 0 0.025 0.05
Regression Coefficients
cg00881696
Cohort Coef N exp:unexp
START 0.0284 174:641
ALHS 0.0141 112:1341
Meta 0.0222 286:1982
0 0.0125 0.025 0.0375
Regression Coefficients
cg12477880
Cohort Coef N exp:unexp
START 0.0586 174:641
ALHS 0.0203 112:1341
Meta 0.0454 286:1982
−0.025 0 0.025 0.05 0.075
Regression Coefficients
cg13989852
Cohort Coef N exp:unexp
START 0.0143 174:641
ALHS 0.0111 112:1341
Meta 0.0132 286:1982
0 0.0125 0.025 0.0375
Regression Coefficients
cg17794604
Cohort Coef N exp:unexp
START 0.0162 174:641
ALHS 0.0088 112:1341
Meta 0.0121 286:1982
0 0.0125 0.025 0.0375
Regression Coefficients
cg04032578
Cohort Coef N exp:unexp
START 0.0147 174:641
ALHS 0.0160 112:1341
Meta 0.0150 286:1982
0 0.0125 0.025 0.0375
Regression Coefficients
cg11429111
Cohort Coef N exp:unexp
START 0.0234 174:641
ALHS 0.0159 112:1341
Meta 0.0202 286:1982
0 0.0125 0.025 0.0375
Regression Coefficients
cg11902777
Cohort Coef N exp:unexp
START −0.0087 174:641
ALHS −0.0020 112:1341
Meta −0.0047 286:1982
−0.025 0 0.025
Regression Coefficients
cg02869559
Cohort Coef N exp:unexp
START 0.0463 174:641
ALHS 0.0202 112:1341
Meta 0.0387 286:1982
−0.025 0 0.025 0.05 0.075
Regression Coefficients

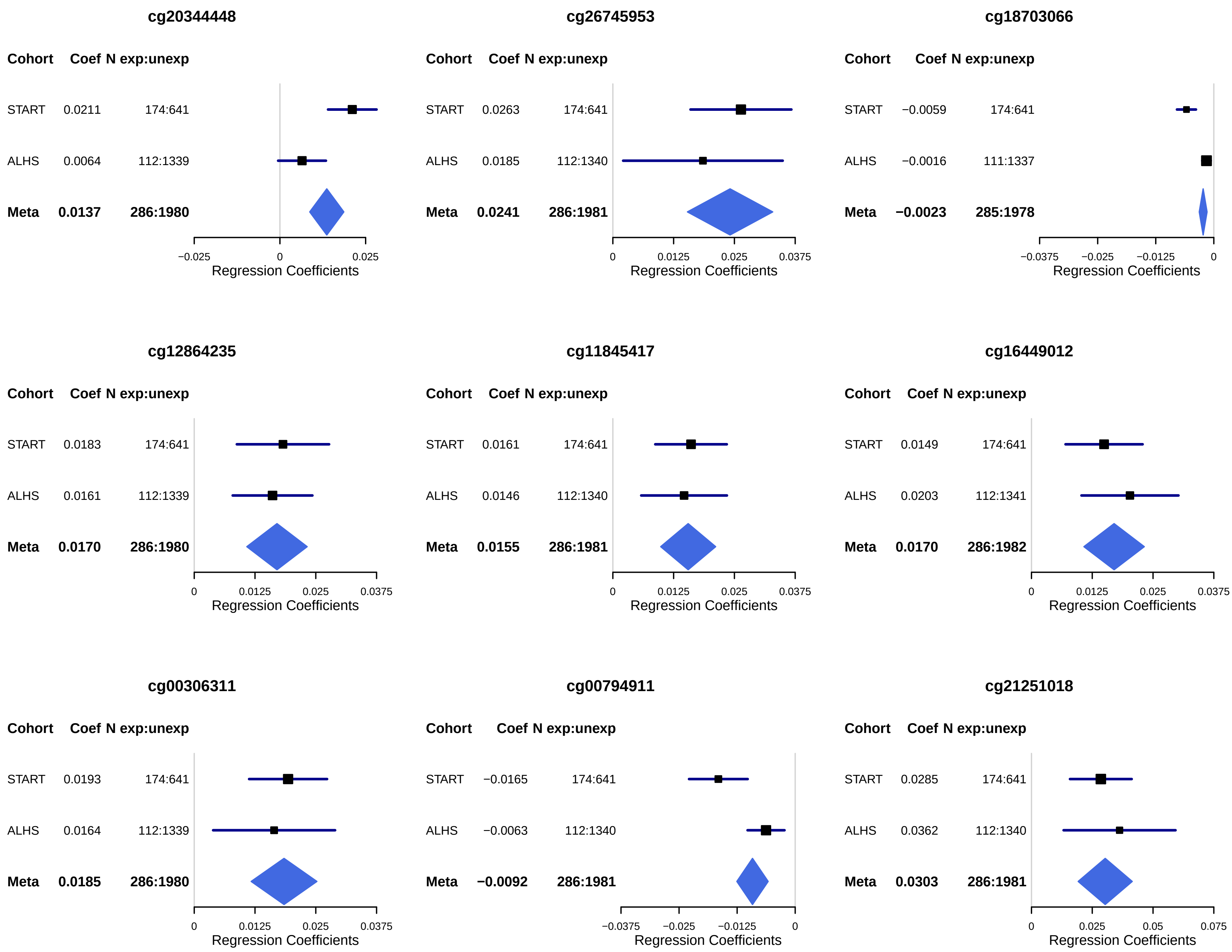
cg20344448
Cohort Coef N exp:unexp
START 0.0211 174:641
ALHS 0.0064 112:1339
Meta 0.0137 286:1980
−0.025 0 0.025
Regression Coefficients
cg26745953
Cohort Coef N exp:unexp
START 0.0263 174:641
ALHS 0.0185 112:1340
Meta 0.0241 286:1981
0 0.0125 0.025 0.0375
Regression Coefficients
cg18703066
Cohort Coef N exp:unexp
START −0.0059 174:641
ALHS −0.0016 111:1337
Meta −0.0023 285:1978
−0.0375 −0.025 −0.0125 0
Regression Coefficients
cg12864235
Cohort Coef N exp:unexp
START 0.0183 174:641
ALHS 0.0161 112:1339
Meta 0.0170 286:1980
0 0.0125 0.025 0.0375
Regression Coefficients
cg11845417
Cohort Coef N exp:unexp
START 0.0161 174:641
ALHS 0.0146 112:1340
Meta 0.0155 286:1981
0 0.0125 0.025 0.0375
Regression Coefficients
cg16449012
Cohort Coef N exp:unexp
START 0.0149 174:641
ALHS 0.0203 112:1341
Meta 0.0170 286:1982
0 0.0125 0.025 0.0375
Regression Coefficients
cg00306311
Cohort Coef N exp:unexp
START 0.0193 174:641
ALHS 0.0164 112:1339
Meta 0.0185 286:1980
0 0.0125 0.025 0.0375
Regression Coefficients
cg00794911
Cohort Coef N exp:unexp
START −0.0165 174:641
ALHS −0.0063 112:1340
Meta −0.0092 286:1981
−0.0375 −0.025 −0.0125 0
Regression Coefficients
cg21251018
Cohort Coef N exp:unexp
START 0.0285 174:641
ALHS 0.0362 112:1340
Meta 0.0303 286:1981
0 0.025 0.05 0.075
Regression Coefficients
