# Supplementary material for: Comprehensive evaluation of smoking exposures and their interactions on DNA methylation
Source: eBioMedicine. 2024 Jan 9;100:104956. doi: 10.1016/j.ebiom.2023.104956 (PMC10825325; doi:10.1016/j.ebiom.2023.104956)
Supplement: Supplemental Consortium Authors [file mmc8.docx]

| **First name** | **Surname** |
| --- | --- |
| Bastiaan | Heijmans |
| Peter | ’t Hoen |
| Joyce | van Meurs |
| Rick | Jansen |
| Lude | Franke |
| Dorret | Boomsma |
| René | Pool |
| Jenny | van Dongen |
| Jouke | Hottenga |
| Marleen | van Greevenbroek |
| Coen | Stehouwer |
| Carla | van der Kallen |
| Casper | Schalkwijk |
| Cisca | Wijmenga |
| Sasha | Zhernakova |
| Ettje | Tigchelaar |
| P. Eline | Slagboom |
| Marian | Beekman |
| Joris | Deelen |
| Diana | Van Heemst |
| Jan | Veldink |
| Leonard | van den Berg |
| Cornelia | van Duijn |
| Bert | Hofman |
| Aaron | Isaacs |
| André | Uitterlinden |
| P. Mila | Jhamai |
| Michael | Verbiest |
| H. Eka | Suchiman |
| Marijn | Verkerk |
| Ruud | van der Breggen |
| Jeroen | van Rooij |
| Nico | Lakenberg |
| Hailiang | Mei |
| Maarten | van Iterson |
| Michiel | van Galen |
| Jan | Bot |
| Dasha | Zhernakova |
| Peter | van ‘t Hof |
| Patrick | Deelen |
| Irene | Nooren |
| Matthijs | Moed |
| Martijn | Vermaat |
| René | Luijk |
| Marc | Bonder |
| Freerk | van Dijk |
| Wibowo | Arindrarto |
| Szymon | Kielbasa |
| Morris | Swertz |
| Erik | van Zwet |
